# Design, Synthesis, Molecular Docking, Pharmacokinetic Properties, and Molecular Dynamics Simulation of Sulfonyl Derivatives of Benzimidazole against Parkinson’s Disease

**DOI:** 10.2174/0109298673337912241007120510

**Published:** 2024-10-24

**Authors:** Subarna Roy, Subhankar Basak, Shristi Roy, Paromita Dey, Hema Barman, Bhagat Singh, Kaushik Sarkar, Subhadeep Sen, Rajesh Kumar Das, Sudhan Debnath, Goutam Biswas

**Affiliations:** 1 Department of Chemistry, Cooch Behar Panchanan Barma University, Cooch Behar, West Bengal, 736101, India;; 2 Department of Chemistry, Indian Institute of Technology Indore, Khandwa Road, Simrol, Madhya Pradesh, 453552, India;; 3 Department of Chemistry and Biochemistry, University of North Carolina at Greensboro, Greensboro, North Carolina 27402, United States;; 4 Department of Chemistry, University of North Bengal, Darjeeling, West Bengal, 734013, India;; 5 Department of Chemistry, Netaji Subhash Mahavidyalaya, Udaipur, Tripura, 799114, India

**Keywords:** Parkinson’s disease, benzimidazole, docking, MD simulation, drug-likeness, Huntington’s disease

## Abstract

**Introduction:**

The disability and mortality related to Parkinson's disease (PD), a neurodegenerative disease, are increasing globally at a faster rate than other neurological disorders. With no permanent cure for PD, there is an urgent need to develop novel and effective anti-PD drugs.

**Methods:**

Targeting monoamine oxidases (MAO), which catalyze the breakdown of neurotransmitters, is one way to treat neurodegenerative diseases. In this context, an initial molecular docking of twenty designed sulfonyl derivatives of benzimidazole against monoamine oxidase B (MAO-B) associated with PD was conducted using AutoDock Vina.

**Results:**

The results were compared with those of the conventional inhibitors, selegiline and rasagiline. Based on the docking score, the *in silico* pharmacokinetic properties (ADME), drug-likeness, and toxicity profiles of the newly synthesized molecules were examined using SwissADME, PreADMET, ProTox-3.0, vNN, and ADMETlab web tools. Then, twelve potential derivatives were synthesized and characterized by IR, ^1^H-NMR, ^13^C-NMR, ^19^F-NMR (for some compounds), and mass spectrometry. Derivatives **2cj** and **1bj** were the two molecules having the best binding affinity of -11.9 and -11.8 kcal/mol, respectively, against MAO-B, exhibiting a higher binding affinity compared to that of some commercially available drugs. A 50 ns MD simulation run was performed to observe the stability of the top two docked complexes, **MAO-B-2cj** and **MAO-B-1bj**, in order to further validate the efficacy of those two substances. Moreover, the MM-PBSA method was used to calculate the final, binding free energy of the simulated (**MAO-B-2cj**) complex.

**Conclusion:**

This study indicates that the binding affinity of most of the hits was superior to that of known MAO inhibitors; therefore, these newly synthesized benzimidazole derivatives may be developed into essential drug candidates for the treatment of PD.

## 1 INTRODUCTION

Population aging is undeniable and has undoubtedly emerged as the primary risk factor for most neurodegenerative diseases, including Alzheimer's disease (AD), Parkinson's disease (PD) [1], amyotrophic lateral sclerosis, spinal muscular atrophy, prion disease, motor neuron disease, Huntington’s disease, and spinocerebellar ataxia. Abnormal aggregation and accumulation of pathogenic amyloid proteins, such as amyloid-β (Aβ), Tau, and α-synuclein (α-syn), play important pathological roles and serve as histological markers [2]. Some studies have reported that low levels of cerebrospinal fluid (CSF) Aβ42 might indicate the beginning of cognitive impairment in PD [3]. It is mostly targeted at Lewy bodies and Lewy nerites-enriched 𝛼-syn amyloid aggregates [2]. In general, PD has depicted some serious dysfunctions among 1-2% of adults aged over 60 years [4]. With advancing age, the prevalence of PD increases almost exponentially, reaching its peak near 80 years of age [5]. The major neuropathological hallmark of PD is the degeneration of dopaminergic neurons in the substantia nigra (SN), which causes striatal dopamine deficiency owing to accumulation of reactive oxygen species (ROS) and aberrant aggregation of α-syn protein [6]. In addition to exhibiting a disorder of physical movement, Parkinson's disease is accompanied by a number of non-motor features, including cognitive impairment, autonomic malfunction, sleeping disorders, depression, and hyposmia (impaired smell) [7]. Monoamine oxidase B (MAO-B) inhibitors, like selegiline **(VI)** and rasagiline **(VII)**, are modestly effective as monotherapy or adjunct therapy in the early stages of PD [8]. However, the majority of medications, including dopaminergic [9], anti-muscarinic, and anti-glutamatergic medicines [10, 11], simply relieve dyskinesia [12] rather than curing PD. The long-term consumption of dopaminergic drugs in the treatment of PD may lead to some complications, including motor and non-motor fluctuations, dyskinesia, and psychosis [13]. Currently, some marketed drugs (Table **1**) are available in the market as a part of anti-PD drug therapy [14-24].

Organosulfur compounds (OSCs) are found within the biological systems of every living organism in the form of certain essential amino acids (cysteine, cystine, and methionine, which are actually protein-building blocks), tripeptide glutathione, several enzymes and coenzymes, vitamins, and hormones [25]. Numerous OSCs are asserted to exhibit strong antioxidant [26] activity as well as antiplatelet [27], immunomodulatory [28], anti-aging [29], anti-bacterial [30], and other pharmacological functions [25]. Methyl-substituted benzimidazole is a widely used pharmacophore in medicinal chemistry, known for its anti-*Hepatitis C virus* (HCV) activity.

Fluorine or fluorinated groups make up approximately 15-16% of all drugs introduced globally, impacting biochemical metabolism. The presence of fluorine can significantly alter the biological properties of a natural product [31]. The medicinal use of the trifluoromethyl (-CF_3_) group containing drugs often includes HIV reverse transcriptase inhibitors (efavirenz (Sustiva)) [32], antidepressants (fluoxetine (Prozac)) [33], and nonsteroidal anti-inflammatory drugs (celecoxib (Celebrex)) [34]. The nitro (-NO_2_) group is a versatile functional group in medicinal chemistry, producing electron-deficient regions in molecules that interact with biological nucleophiles like proteins, amino acids, and enzymes. On the contrary, the (-NO_2_) group poses toxicity issues like mutagenicity and genotoxicity and is often labeled as structural alert or toxicophoric [35]. Apart from these shortcomings, preclinical and preliminary screening assays have demonstrated the bioactive potential of a library of FDA-approved drugs and prodrugs containing nitro groups as antibacterial (metronidazole) [36], antitubercular (delamanid) [37], antiparasitic (tinidazole) [38], and other bioactive agents [39, 40].

Computational pharmacology is a rapidly growing field that utilizes computer-based software and databases to develop innovative techniques for creating and analyzing novel, sustainable drugs. This approach saves time and human and material resources compared to experimental and clinical methodologies [41]. *In-silico* ADMET prediction is the easiest and fastest technique for evaluating medications prior to initiating clinical trials on the basis of the data gathered using various freely-accessible web tools. It is crucial for screening new chemical species and their properties, determining if they can be used as potential medications. Web-based tools, like ADMETlab, ProTox-3.0, vNN, PreADMET, and SwissADME, have been constructed to predict ADMET attributes.

Nitrogenous heterocyclic compounds, particularly benzo-fused azoles, are crucial for the metabolism of all life forms, with their diverse characteristics and applications being significant in industrial and medicinal chemistry [42-46]. A large number of benzimidazole derivatives serve an active role in pharmaceuticals as anticancer [47], antiviral [48], antihistaminic [49], analgesic [50], and many other therapeutic agents [51]. Benzimidazole derivatives, like omeprazole, lansoprazole, and pantoprazole, are widely known commercially available proton-pump inhibitors, used mainly in the treatment of peptic ulcer and gastroesophageal reflux disease (GERD) [52]. Nowadays, benzimidazole derivatives are frequently employed in clinical practice as highly effective therapeutic agents for the treatment of neurodegenerative disorders, such as Parkinson's disease (PD) [53]. Recent research focuses on discovering novel anti-PD drugs that inhibit MAO-B activity and exhibit neuroprotective and antioxidant properties. *In vitro* evaluations of benzimidazole derivatives with hydroxy and methoxy arylhydrazone fragments on neuroblastoma SH-SY5Y cells and isolated rat synaptosomes found them safe and effective. In another study, the potential anti-PD activity of sulfonyl derivatives of fluoro-substituted benzimidazoles against PD-inducing protein MAO-B has been reported [54].

**Fig. (1):**
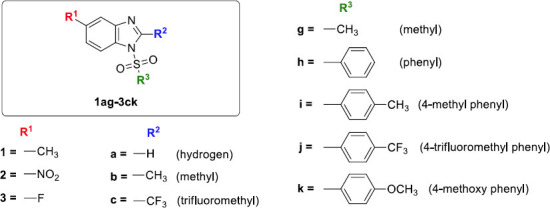
Twenty designed benzimidazole molecules.

In our previous study [54], it has been observed that the sulfonyl derivatives of 5-fluoro-substituted benzimidazole exhibit excellent anti-PD drug-likeness. Therefore, based on the results from our previous observations, we envisioned that suitable benzimidazole derivatives could be designed and synthesized to check their biological activity and therapeutic significance. Initially, twenty benzimidazole derivatives (Fig. **1**) were designed based on the reagent availability and our previous findings and then docked against MAO-B protein employing Autodock Vina. Based on the satisfactory results, web tools, like SwissADME, ProTox-3.0, vNN, PreADMET, and ADMETlab, were used to examine the *in-silico* pharmacokinetic (ADME) properties, drug-likeness, and toxicity profiles/toxicological endpoints of twelve derivatives exhibiting good docking scores. Incorporating structural changes like new functional group introduction in the aromatic rings, the present study produced superior outcomes to those of our prior report. Based on the data from theoretical methods, twelve compounds were synthesized possessing good docking scores and a notable quality of drug-likeness. In comparison with our previous study, we employed MD simulation for the best-docked molecules in this report. The dynamic behavior (stability) of the two best-docked complexes was observed through a 50 ns MD simulation run. Furthermore, MM-PBSA calculation was performed to estimate the binding free energy (kJ/mol) of the MD simulated complex, which actually validates the docking as well as simulation results.

## 2 MATERIALS AND METHODS

### 2.1 Molecular Docking

#### 2.1.1 Protein Preparation

The X-ray crystal structure of MAO-B with PDB ID: 2C65_A (1.70 Å) was retrieved from the Research Collaboratory for Structural Bioinformatics (RCSB) Protein Data Bank [55]. After importing the protein crystal structure into AutoDockTools 1.5.6, heteroatoms and water molecules were removed. The Kollman charge was then computed after adding polar hydrogen. The water molecules and hetero atoms were removed from the crystal structure after being imported into AutoDock Tools 1.5.6. [56], Polar hydrogen was then inserted, followed by computing the Kollman charge. Finally, the protein was saved in pdbqt format.

#### 2.1.2 Ligand Preparation

The ChemDraw Professional 15 was used to draw the structure of the compounds, which were then saved as sdf files. The OpenBabel software was used to translate ligands into PDB format [57]. Additionally, charges were allocated, torsional angles were modified, the ligands were optimized using the MGL tool, and finally, the ligands were converted into pdbqt format [54, 58].

#### 2.1.3 Docking Procedure

**Fig. (2):**
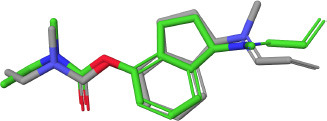
Superposition of docked 4CR on its originally bound native conformation in X-ray ligand–enzyme complex of 4CR: (4-(N-methyl-N-ethyl-carbamoyloxy)-N-methyl-N-propargyl-1(R)-aminoindan) and RMSD was calculated.

Using AutoDock Vina (ADV) [59] software, all chemical libraries underwent molecular testing. During docking, the protein was projected to be rigid, but the ligands were assumed to be flexible. The MAO B forms a complex with 4-(N-methyl-N-ethyl-carba-moyloxy)-Nmethyl-N-propargyl-1(R)-aminoindan in the crystal structure. The grid for 2C65 was made using the co-crystallized ligand, and the size of the grid could be altered depending on the residues in the active site (Fig. **2**). While docking exhaustiveness was set to 50, a grid box of size 32.0 × 34.0 × 32.0 Å3 with the coordinates center_x = 49.351, y = 155.638, and z = 25.685 was employed. The Discovery Studio 2021 Client [60] (BIOVIA 2016) and PyMOL [61] were used to help visualize the top docking pose of the ADV output file. The molecular docking study was carried out using a Core (TM) 2 Duo CPU computer running Windows 10 with 64-bit OS architecture.

#### 2.1.4 Validation of Docking

Validating a docking protocol is an important step in computational chemistry and drug development that ensures the reliability and accuracy of molecular docking studies. In the present study, the correctness of the docking was evaluated by computing the root mean square deviation (RMSD). Re-docking is the process of removing the co-ligand (4-(N-methyl-N-ethyl-carba-moyloxy)-Nmethyl-N-propargyl-1(R)-aminoindan) and then docking it into the binding site of the protein. Then, the docked conformations of the co-ligand were superimposed with its original crystallographic bound conformation, and the RMSD was calculated.

### 2.2 Physicochemical Property, Drug-likeness, and Pharmacokinetics Prediction

*In silico* absorption, distribution, metabolism, and excretion (ADME) predictions are one of the most crucial and pertinent criteria for determining the drug-likeliness of selected hits. In earlier days, the ADME characteristics of medicinal molecules were evaluated at the last stage of the drug discovery process [41]. An *in-silico* method can be used to predict ADME properties in the early stages of modern drug development. Due to weak ADME properties, 60% of drug candidates failed during the development period. Early identification of these characteristics would, therefore, lead to lesser drug development costs [54].

Twelve (**1ai-3bj**) of the twenty compounds were synthesized based on the docking study and demonstrated high docking scores. Pharmaceutically significant parameters and physically relevant characteristics of the target benzimidazoles (**1ai-3bj**) were predicted. The physicochemical properties (molar refractivity, topological polar surface area, number of hydrogen bond acceptors), water solubility, pharmacokinetic properties (gastrointestinal tract (GI) absorption, P-gp substrate, cytochrome-P enzyme inhibition, and skin permeation (log Kp)), which are essential components for predicting the absorption and distribution of drugs within the body, and drug-likeness (Lipinski’s rule of five) were predicted *via* SwissADME [62] and BBB permeation by PreADMET [63]. The stated formula % Abs = 109 - 0.345 TPSA was used to compute the ligands' percent absorptions (% Abs) [64]. The ADMETlab [65] web server was employed to predict physicochemical properties (logS, logD, logP). The results regarding these properties are as follows: ***Absorption***: Caco-2 cell permeability (Caco-2), P-gp inhibitor/substrate (P-gp), Human Intestinal Absorption (HIA), 20% bioavailability (F20%) and 30% bioavailability (F30%); ***Distribution***: Plasma Protein Binding (PPB), Blood-Brain Barrier (BBB) and Volume of Distribution (VD); and ***Excretion***: Half-Life (T1/2) and Clearance (CL). The properties of Caco-2, VD, PPB, CL, and T1/2 were expressed numerically, whereas the rest of the pharmacokinetic parameters were expressed categorically [65]. ***Metabolism***-related parameters were predicted by carrying out human liver microsomal (HLM) assay using variable nearest neighbor (vNN) method [66]. The twelve benzimidazole derivatives (**1ai-3bj**) were screened to obtain the best candidate considering the molecular descriptors and properties produced by all webtools/webservers.

### 2.3 Toxicity Prediction

In order to analyze the toxicity profile of the twelve benzimidazoles and the two reference drugs, a total of 19 parameters were predicted. The toxicological endpoints (hepatotoxicity, carcinogenicity, immuno-toxicity, and mutagenicity) and the level of toxicity (LD50, mg/Kg) of the examined benzimidazoles were assessed using ProTox-3.0 server [67]. Toxicity profiles, the hERG potassium channel inhibition (cardiotoxicity), H-HT (Human Hepatotoxicity), AMES (Ames Mutagenicity), and SkinSen (Skin sensitization), were predicted using ADMETlab [65]. The vNN method [66] was applied to predict drug-induced liver injury (DILI), mitochondrial membrane potential (MMP) toxicity, and cytotoxicity traits.

### 2.4 Materials and Instrumentations

All the chemicals and solvents were purchased from Sigma Aldrich, BLD Pharmatech, and other local vendors and were used as received without further purification. Analytical thin layer chromatography (TLC) was done using UV-active Merck 60 F254 pre-coated silica gel plates (0.2 mm thick). TLC plates were analyzed by exposure to ultraviolet (UV) light. Retention factors (*R*f) for the compounds **1ai-3bj** were determined using TLC. The rotary distillation apparatus, Superfit Rotavap Model: PBV-7D, was used for distillation. Moreover, the centrifuge machine, REMI Model: R-4C, was used during the procedure of decantation.

### 2.5 Chemical Synthesis

**Scheme 1:**
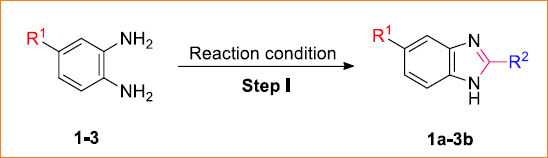
Synthetic route to intermediates **1a-3b**.

**Scheme 2:**
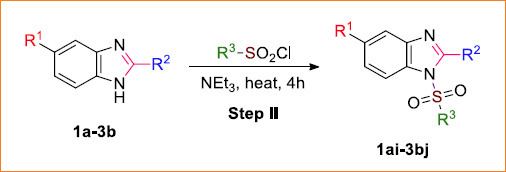
Synthetic route to products **1ai-3bj**.

A series of 1-alkyl or 1-aryl sulfonyl benzimidazole derivatives (**1ai-3bj**) were synthesized using the synthetic route inscribed in Schemes **1** and **2**, respectively. The first step involved the synthesis of intermediate benzimidazoles derived from the respective 4-substituted benzene-1,2-diamine (namely, 4-methylbenzene-1,2-dia-mine, 4-nitrobenzene-1,2-diamine and 4-fluorobenzene-1,2-diamine). Those benzene-1,2-diamines were refluxed with various organic acids (formic acid, acetic acid, and trifluoroacetic acid) to afford respective intermediate benzimidazoles **1a-3b**. The formation of those intermediates was verified by TLC, and then they were recrystallized in hot water. In the second step, those intermediate benzimidazoles were further treated with several sulfonyl chlorides (namely, methane sulfonyl chloride, benzene sulfonyl chloride, tosyl chloride, 4-trifluoromethylbenzene-1-sulfonyl chloride, and 4-methoxybenzene-1-sulfonyl chloride) in the presence of triethylamine (base) and chloroform (solvent) to generate the expected products, *i.e.*, respective, sulfonyl derivatives **1ai-3bj**. All the resultant products were further purified by the decantation method, and their formation was confirmed by NMR data analysis. Figures of 1H-NMR for the reported compounds **1a-3b**, **1bi**, **2ai,** and **2bi** and figures of all the spectral data for the rest (novel) of the compounds are provided in the supporting information (Fig. **S1-S59**).

### 2.6 General Procedure I: Synthesis of Intermediate 5-substituted Benzimidazoles

For this purpose, 20 mmol of 4-substituted benzene-1,2-diamine (**1**-**3**) dissolved in organic acid (163 mmol of 90% formic acid; 25 mmol of glacial acetic acid or 13 mmol of trifluoroacetic acid (TFA) in the presence of 4 (M) hydrochloric acid (15 mL: 5 mL conc. HCl + 10 mL water)) taken in a 250 mL one-necked round-bottomed flask was refluxed on silicon-oil bath under condenser at 110°C for several hours (4 h for formic acid; 12 h each for acetic acid and TFA). After the reaction was completed by TLC monitoring, the reaction mixture was cooled, and 10% sodium hydroxide (NaOH) solution was slowly added to it with constant stirring until the mixture became just alkaline to litmus. Then, the reaction mixture was quenched with water and extracted from ethyl acetate three times. The combined layers of organic portion were dried over Na_2_SO_4_, filtered, and concentrated under a rotary distillation apparatus, leaving the crude product. The crude product was dissolved in the minimum amount of boiling water (<100 mL), followed by the addition of ~2g decolorizing active charcoal, and was left to digest for 15 min. In hot conditions, the extract was filtered rapidly through a preheated Buchner funnel using a pump to collect the filtrate in a flask. The filtrate was then cooled to about 10°C, followed by washing with cold water and drying at 100°C to get fine crystals of the desired benzimidazole intermediate (**1a**-**3b**) [54].

#### 2.6.1 5-Methyl-1H-benzo[d]imidazole (1a)

Compound **1a** was synthesized following general procedure I from 4-methylbenzene-1,2-diamine (**1**) (2.44 g, 20 mmol) and 90% formic acid (163 mmol). The formation of **1a** was verified by TLC monitoring and was recrystallized in hot water. Off-white solid; Yield 1.96 g, 74%; m. p. 112-117°C; ^1^H NMR (400 MHz, CDCl_3_) δ (ppm): 8.04 (s, 1H), 7.55 (d, *J* = 8.2 Hz, 1H), 7.43 (s, 1H), 7.11 (d, *J* = 8.2 Hz, 1H), 2.47 (s, 3H) [68].

#### 2.6.2 2,5-Dimethyl-1H-benzo[d]imidazole (1b)

Compound **1b** was synthesized following the general procedure I from 4-methylbenzene-1,2-diamine (**1**) (2.44 g, 20 mmol) and glacial acetic acid (25 mmol) in the presence of 4 (M) hydrochloric acid. The formation of 1b was verified by TLC monitoring and was recrystallized from hot water. Tomato-red solid; Yield 2.46 g, 84%; m. p. 202-205°C; ^1^H NMR (400 MHz, CDCl_3_) δ (ppm): 7.41 (br s, 1H), 7.29 (s, 1H), 7.02 (d, *J* = 8.1 Hz, 1H), 2.59 (s, 3H), 2.44 (s, 3H) [68].

#### 2.6.3 5-Nitro-1H-benzo[d]imidazole (2a)

Compound 2a was synthesized following general procedure I from 4-nitrobenzene-1,2-diamine (2) (3.06 g, 20 mmol) and 90% formic acid (163 mmol). The formation of 2a was verified by TLC monitoring and was recrystallized in hot water. Deep brown solid; Yield 2.02 g, 62%; m. p. 202-205°C; ^1^H NMR (400 MHz, DMSO-D_6_) δ (ppm): 8.52 (s, 1H), 8.48 (s, 1H), 8.09 (dd, *J* = 8.9, 1.6 Hz, 1H), 7.74 (d, *J* = 8.9 Hz, 1H) [69].

#### 2.6.4 2-Methyl-5-nitro-1H-benzo[d]imidazole (2b)

Compound 2b was synthesized following the general procedure I from 4-nitrobenzene-1,2-diamine (2) (3.06 g, 20 mmol) and glacial acetic acid (25 mmol) in the presence of 4 (M) hydrochloric acid. The formation of 2b was verified by TLC monitoring and was recrystallized in hot water. Bright orange solid; Yield 2.52 g, 71%; m. p. 224°C; ^1^H NMR (600 MHz, DMSO-D_6_) δ (ppm): 8.36 (d, *J* = 2.1 Hz, 1H), 8.06 (dd, *J* = 8.7, 2.2 Hz, 1H), 7.64 (d, *J* = 8.9 Hz, 1H), 2.09 (s, 3H) [70].

#### 2.6.5 5-Nitro-2-(trifluoromethyl)-1H-benzo[d] imidazole (2c)

Compound **2c** was synthesized following the general procedure I from 4-nitrobenzene-1,2-diamine (**2**) (3.06 g, 20 mmol) and TFA (13 mmol) in the presence of 4 (M) hydrochloric acid. The formation of 2c was verified by TLC monitoring and was recrystallized from hot water. Brown solid; Yield 2.54 g, 55%; m. p. 148-151°C; ^1^H NMR (400 MHz, CD_3_COCD_3_) δ (ppm): 8.57 – 8.39 (m, 1H), 8.20 – 8.05 (m, 1H), 7.83 – 7.66 (m, 1H) [71].

#### 2.6.6 5-Fluoro-1H-benzo[d]imidazole (3a)

Compound **3a** was synthesized following general procedure I from 4-fluorobenzene-1,2-diamine (**3**) (2.52 g, 20 mmol) and 90% formic acid (163 mmol). The formation of **3a** was verified by TLC monitoring and was recrystallized in hot water. White solid; Yield 1.93 g, 71%; m. p. 131-133°C; ^1^H NMR (400 MHz, CDCl_3_) δ (ppm): 8.16 (s, 1H), 7.62 (dd, *J* = 8.8, 4.8 Hz, ^1^H), 7.35 (dd, *J* = 8.8, 2.4 Hz, 1H), 7.08 (td, *J* = 9.2, 2.4 Hz, 1H) [54].

#### 2.6.7 5-Fluoro-2-methyl-1H-benzo[d]imidazole (3b)

Compound **3b** was synthesized following the general procedure I from 4-fluorobenzene-1,2-diamine (**3**) (2.52 g, 20 mmol) and glacial acetic acid (25 mmol) in the presence of 4 (M) hydrochloric acid. The formation of 3b was verified by TLC monitoring and was recrystallized from hot water. White solid; Yield 2.13 g, 71%; m. p. 177-179°C; ^1^H NMR (400 MHz, CDCl_3_) δ (ppm): 7.46 (dd, *J* = 8.8, 4.8 Hz, 1H), 7.24 (d, *J* = 8.8 Hz, 1H), 6.99 (t, *J* = 9.2 Hz, 1H), 2.64 (s, 3H) [54].

### 2.7 General procedure II: Alkyl- or aryl-sulfonation of intermediate benzimidazoles

A mixture of 0.1 g intermediate 5-substituted benzimidazole (**1a**-**3b**) dissolved in chloroform (3 mL) (less dissolving 5-nitrobenzimidazoles (**2a**-**2c**) were dissolved in the solvent mixture (1:1) of chloroform and tetrahydrofuran (THF)), sulfonyl chloride (tosyl chloride (1.20 mmol) for **1ai**, **1bi** and **2ai**-**2ci**; 4-(trifluoromethyl)benzene-1-sulfonyl chloride (1.09 mmol) for **1aj**-**1bj**, **2aj**-**2cj** and **3aj**-**3bj**), and triethylamine (0.1 mL, 0.75 mmol) was heated in water bath for 4 h. In the case of **2cj**, after heating, the mixture was stirred with a magnetic stirrer for an extra 12 h. After completion of the reaction by TLC monitoring, the reaction mixture was evaporated under vacuum, leaving the desired product. Then, the product was purified using the decantation method. In this method, 3-4 mL of hexane was added to the product, and the solution was sonicated. Such-wise, the extracted hexane portion (containing the unreacted sulfonyl chloride) and the solid part were collected separately, followed by the repetition of this process for 2-3 times more. Then, 3-4 mL of ethyl acetate (chloroform in the case of 1ai and 1bi) was introduced to the separated solid portion, followed by sonication. The ethyl acetate extract was then centrifuged to collect the precipitated solid part as the purified product (**1ai**-**3bj**). The complete decantation process was monitored by employing TLC [54].

#### 2.7.1 5-Methyl-1-tosyl-1H-benzo[d]imidazole (1ai)

Compound **1ai** was synthesized following general procedure II from 5-methyl-1H-benzo[d]imidazole (**1a**) (0.1 g) and tosyl chloride (1.20 mmol). The formation of **1ai** was verified by TLC monitoring and purified *via* the decantation process. Muddy grey solid; Yield 0.14 g, 66%; R_f_: 0.66 (CH_3_OH/CHCl_3_ 1:19, UV). IR (ν, cm^-1^): 3110-3026 (aromatic C-H stretch), 2980-2915 (sp3 C-H stretch), 1594-1422 (aromatic C=C stretch), 1367 (asymmetric S=O stretch), 1153 (symmetric S=O stretch), 1034 (C-N stretch); ^1^H NMR (400 MHz, CDCl_3_) δ (ppm): 8.31 (s, 1H), 7.85 (d, *J* = 4.7 Hz, 2H), 7.70 (d, *J* = 8.3 Hz, 1H), 7.52 (s, 1H), 7.29 (d, *J* = 7.3 Hz, 2H), 7.18 (d, *J* = 8.3 Hz, 1H), 2.42 (s, 3H), 2.35 (s, 3H); ^13^C NMR (100 MHz, CDCl_3_) δ (ppm): 146.2 (Ar-C), 144.4 (Ar-C), 141.3 (Ar-C), 136.0 (Ar-C), 134.8 (Ar-C), 130.4 (Ar-C), 128.8 (Ar-C), 127.3 (Ar-C), 126.9 (Ar-C), 120.9 (Ar-C), 112.1 (Ar-C), 21.8 (CH_3_), 8.7 (CH_3_). MS (m/z): 287.08 [M+H]^+^. Anal. Calcd. for C_15_H_14_N_2_O_2_S: C, 62.92; H, 4.93; N, 9.78; Found: C, 62.79; H, 4.94; N, 9.71.

#### 2.7.2 5-Methyl-1-((4-(trifluoromethyl) phenyl) sulfonyl)-1H-benzo[d]imidazole (1aj)

Compound **1aj** was synthesized following general procedure II from 5-methyl-1H-benzo[d]imidazole (**1a**) (0.1 g) and 4-(trifluoromethyl)benzene-1-sulfonyl chloride (1.09 mmol). The formation of **1aj** was verified by TLC monitoring and purified *via* the decantation process. Grey solid; Yield 0.14 g, 53%; R_f_: 0.86 (CH_3_OH/CHCl_3_ 1:19, UV). IR (ν, cm^-1^): 3120-3010 (aromatic C-H stretch), 2940-2850 (sp^3^ C-H stretch), 1620-1450 (aromatic C=C stretch), 1320 (asymmetric S=O stretch), 1230 (symmetric S=O stretch), 1180-1060 (C-F stretch), 1010 (C-N stretch); ^1^H NMR (400 MHz, CD_3_COCD_3_) δ (ppm): 9.36 (s, 1H), 8.02 (dd, *J* = 12.4, 7.9 Hz, 2H), 7.81 (dd, *J* = 12.9, 8.5 Hz, 1H), 7.75 – 7.62 (m, 2H), 7.38 (dd, *J* = 12.5, 8.8 Hz, 1H), 6.88 (ddd, *J* = 11.8, 8.0, 5.8 Hz, 1H), 2.00 (s, 3H); ^13^C NMR (100 MHz, CD_3_COCD_3_) δ (ppm): 151.2 (Ar-C), 139.8 (Ar-C), 136.9 (Ar-C), 131.3 (Ar-C), 129.1 (Ar-C), 127.9 (Ar-C), 126.8 (Ar-C), 125.1 (Ar-C), 123.0 (Ar-C), 114.3 (Ar-C), 114.1 (Ar-C), 20.7 (CF3), 8.2 (CH3); ^19^F NMR (376 MHz, CD_3_COCD_3_) δ (ppm): -63.00 (s). Anal. Calcd. for C_15_H_11_F_3_N_2_O_2_S: C, 52.94; H, 3.26; N, 8.23; Found: C, 52.81; H, 3.22; N, 8.27.

#### 2.7.3 2,5-Dimethyl-1-tosyl-1H-benzo[d]imidazole (1bi)

Compound **1bi** was synthesized following general procedure II from 2,5-dimethyl-1H-benzo[d]imidazole (**1b**) (0.1 g) and tosyl chloride (1.20 mmol). The formation of 1bi was verified by TLC monitoring and purified *via* the decantation process. Black solid; Yield 0.12 g, 61%; R_f_: 0.83 (CH_3_OH/CHCl_3_ 1:19, UV). IR (ν, cm^-1^): 3150-3050 (aromatic C-H stretch), 2920-2860 (sp^3^ C-H stretch), 1600-1430 (aromatic C=C stretch), 1370 (asymmetric S=O stretch), 1170 (symmetric S=O stretch), 1010 (C-N stretch); ^1^H NMR (400 MHz, CDCl_3_) δ (ppm): 7.86 (dd, *J* = 8.1, 5.3 Hz, 2H), 7.79 (d, *J* = 3.6 Hz, 1H), 7.48 (d, *J* = 8.1 Hz, 1H), 7.28 (d, *J* = 6.5 Hz, 2H), 7.13 (t, *J* = 8.8 Hz, 1H), 2.77 (s, 3H), 2.42 (s, 3H), 2.36 (s, 3H); ^13^C NMR (100 MHz, CDCl_3_) δ (ppm): 151.1 (Ar-C), 146.0 (Ar-C), 142.2 (Ar-C), 139.9 (Ar-C), 136.5 (Ar-C), 135.3 (Ar-C), 133.5 (Ar-C), 130.3 (Ar-C), 126.8 (Ar-C), 119.7 (Ar-C), 113.3 (Ar-C), 29.8 (CH_3_), 21.6 (CH_3_), 17.0 (CH_3_). MS (m/z): 301.10 [M+H]^+^. Anal. Calcd. for C_16_H_16_N_2_O_2_S: C, 63.98; H, 5.37; N, 9.33; Found: C, 63.88; H, 5.39; N, 9.29 [72].

#### 2.7.4 2,5-Dimethyl-1-((4-(trifluoromethyl) phenyl) sulfonyl)-1H-benzo[d]imidazole (1bj)

Compound **1bj** was synthesized following general procedure II from 2,5-dimethyl-1H-benzo[d]imidazole (**1b**) (0.1 g) and 4-(trifluoromethyl)benzene-1-sulfonyl chloride (1.09 mmol). The formation of **1bj** was verified by TLC monitoring and purified *via* the decantation process. Greyish white solid; Yield 0.11 g, 47%; R_f_: 0.58 (CH_3_OH/CHCl_3_ 1:19, UV). IR (ν, cm^-1^): 3110-2990 (aromatic C-H stretch), 2960-2860 (sp^3^ C-H stretch), 1610-1550 (aromatic C=C stretch), 1320 (asymmetric S=O stretch), 1290-1230 (C-F stretch), 1170 (symmetric S=O stretch), 1010 (C-N stretch); ^1^H NMR (400 MHz, CDCl_3_) δ (ppm): 8.00 (d, *J* = 3.5 Hz, 2H), 7.84 (d, *J* = 8.4 Hz, 1H), 7.74 (d, *J* = 7.7 Hz, 2H), 7.51 (t, *J* = 8.4 Hz, 1H), 7.16 (t, *J* = 7.1 Hz, 1H), 2.78 (s, 3H), 2.43 (s, 3H); ^13^C NMR (100 MHz, CDCl_3_) δ (ppm): 151.2 (Ar-C), 142.3 (Ar-C), 140.0 (Ar-C), 136.3 (Ar-C), 135.5 (Ar-C), 133.2 (Ar-C), 130.9 (Ar-C), 127.3 (Ar-C), 126.5 (Ar-C), 120.0 (Ar-C), 113.4 (Ar-C), 22.1 (CF_3_), 21.4 (CH_3_), 17.1 (CH_3_); ^19^F NMR (376 MHz, CDCl_3_) δ (ppm): -63.34 (s). MS (m/z): 355.07 [M+H]^+^. Anal. Calcd. for C_16_H_13_F_3_N_2_O_2_S: C, 54.23; H, 3.70; N, 7.91; Found: C, 54.27; H, 3.68; N, 7.94.

#### 2.7.5 5-Nitro-1-tosyl-1H-benzo[d]imidazole (2ai)

Compound **2ai** was synthesized following general procedure II from 5-nitro-1H-benzo[d]imidazole (**2a**) (0.1 g) and tosyl chloride (1.20 mmol). The formation of 2ai was verified by TLC monitoring and purified *via* the decantation process. Yellow solid; Yield 0.11 g, 55%; R_f_: 0.80 (CH_3_OH/CHCl_3_ 1:19, UV). IR (ν, cm^-1^): 3109 (aromatic C-H stretch), 2978-2925 (sp^3^ C-H stretch), 1593 and 1488-1431 (aromatic C=C stretch), 1520 and 1340 (N=O stretch), 1383 (asymmetric S=O stretch), 1170 (symmetric S=O stretch), 1027-1012 (C-N stretch); ^1^H NMR (400 MHz, CDCl_3_) δ (ppm): 8.74 (d, *J* = 2.0 Hz, 1H), 8.57 (s, 1H), 8.26 (dd, *J* = 8.9, 2.1 Hz, 1H), 7.92 (d, *J* = 8.3 Hz, 2H), 7.85 (d, *J* = 8.9 Hz, 2H), 7.37 (s, 1H), 2.39 (s, 3H); ^13^C NMR (100 MHz, CDCl_3_) δ (ppm): 148.2 (Ar-C), 147.3 (Ar-C), 145.5 (Ar-C), 133.9 (Ar-C), 130.9 (Ar-C), 127.5 (Ar-C), 125.9 (Ar-C), 121.5 (Ar-C), 120.5 (Ar-C), 112.8 (Ar-C), 109.3 (Ar-C), 21.9 (CH_3_). MS (m/z): 318.05 [M+H]^+^. Anal. Calcd. for C_14_H_11_N_3_O_4_S: C, 52.99; H, 3.49; N, 13.24; Found: C, 52.92; H, 3.43; N, 13.28 [73].

#### 2.7.6 5-Nitro-1-((4-(trifluoromethyl)phenyl)sulfonyl)-1H-benzo[d]imidazole (2aj)

Compound **2aj** was synthesized following general procedure II from 5-nitro-1H-benzo[d]imidazole (**2a**) (0.1 g) and 4-(trifluoromethyl)benzene-1-sulfonyl chloride (1.09 mmol). The formation of **2aj** was verified by TLC monitoring and purified *via* the decantation process. White solid; Yield 0.10 g, 44%; R_f_: 0.84 (CH_3_OH/CHCl_3_ 1:19, UV). IR (ν, cm^-1^): 3100 (aromatic C-H stretch), 1620-1590 (aromatic C=C stretch), 1520 and 1320 (N=O stretch), 1390 (asymmetric S=O stretch), 1260-1160 (C-F stretch), 1140 (symmetric S=O stretch), 1060-1010 (C-N stretch); ^1^H NMR (600 MHz, CDCl_3_) δ (ppm): 8.68 (s, 1H), 8.55 (s, 1H), 8.36 (d, *J* = 9.0 Hz, 1H), 8.18 (d, *J* = 8.2 Hz, 2H), 8.01 (d, *J* = 9.0 Hz, 1H), 7.87 (t, *J* = 7.9 Hz, 2H); ^13^C NMR (150 MHz, CDCl_3_) δ (ppm): 148.2 (Ar-C), 146.9 (Ar-C), 143.9 (Ar-C), 140.4 (Ar-C), 137.0 (Ar-C), 134.6 (Ar-C), 128.0 (Ar-C), 127.4 (Ar-C), 121.5 (Ar-C), 117.8 (Ar-C), 112.7 (Ar-C), 29.8 (CF_3_); ^19^F NMR (376 MHz, CD_3_OD) δ (ppm): -64.96 (s). MS (m/z): 372.02 [M+H]^+^. Anal. Calcd. for C_14_H_8_F_3_N_3_O_4_S: C, 45.29; H, 2.17; N, 11.32; Found: C, 45.25; H, 2.20; N, 11.28.

#### 2.7.7 2-Methyl-5-nitro-1-tosyl-1H-benzo[d]imidazole (2bi)

Compound **2bi** was synthesized following general procedure II from 2-methyl-5-nitro-1H-benzo[d] imidazole (**2b**) (0.1 g) and tosyl chloride (1.20 mmol). The formation of **2bi** was verified by TLC monitoring and purified *via* the decantation process. Dark brown solid; Yield 0.10 g, 54%; R_f_: 0.77 (CH_3_OH/CHCl_3_ 1:19, UV). IR (ν, cm^-1^): 3120-3090 (aromatic C-H stretch), 2970-2840 (sp^3^ C-H stretch), 1600 and 1430 (aromatic C=C stretch), 1520 and 1340 (N=O stretch), 1370 (asymmetric S=O stretch), 1170 (symmetric S=O stretch), 1090-1040 (C-N stretch); ^1^H NMR (400 MHz, CDCl_3_) δ (ppm): 8.92 (d, *J* = 1.8 Hz, 1H), 8.47 (d, *J* = 1.7 Hz, 1H), 8.24 (td, *J* = 9.0, 1.9 Hz, 2H), 7.82 (dd, *J* = 14.9, 8.3 Hz, 2H), 7.34 (d, *J* = 4.4 Hz, 1H), 2.82 (s, 3H), 2.40 (s, 3H); ^13^C NMR (100 MHz, CDCl_3_) δ (ppm): 154.8 (Ar-C), 147.1 (Ar-C), 145.2 (Ar-C), 141.8 (Ar-C), 134.6 (Ar-C), 130.7 (Ar-C), 127.1 (Ar-C), 120.2 (Ar-C), 115.9 (Ar-C), 113.6 (Ar-C), 110.1 (Ar-C), 21.9 (CH_3_), 17.2 (CH_3_). MS (m/z): 332.07 [M+H]^+^. Anal. Calcd. for C_15_H_13_N_3_O_4_S: C, 54.37; H, 3.95; N, 12.68; Found: C, 54.41; H, 3.91; N, 12.63 [74].

#### 2.7.8 2-Methyl-5-nitro-1-((4-(trifluoromethyl)phenyl) sulfonyl)-1H-benzo[d]imidazole (2bj)

Compound **2bj** was synthesized following general procedure II from 2-methyl-5-nitro-1H-benzo[d] imidazole (**2b**) (0.1 g) and 4-(trifluoromethyl)benzene-1-sulfonyl chloride (1.09 mmol). The formation of **2bj** was verified by TLC monitoring and purified *via* the decantation process. Deep yellow solid; Yield 0.10 g, 45%; R_f_: 0.70 (CH_3_OH/CHCl_3_ 1:19, UV). IR (ν, cm^-1^): 3120-2960 (aromatic C-H stretch), 2920-2850 (sp^3^ C-H stretch), 1620-1550 and 1460-1370 (aromatic C=C stretch), 1520 and 1320 (N=O stretch), 1340 (asymmetric S=O stretch), 1260 and 1150-1090 (C-F stretch), 1170 (symmetric S=O stretch), 1060-1000 (C-N stretch); ^1^H NMR (600 MHz, CDCl_3_) δ (ppm): 8.94 (d, *J* = 2.1 Hz, 1H), 8.53 (d, *J* = 2.1 Hz, 1H), 8.34 – 8.27 (m, 1H), 8.13 (dd, *J* = 19.8, 8.7 Hz, 2H), 7.85 (t, *J* = 7.8 Hz, 2H), 2.86 (s, 3H); ^13^C NMR (100 MHz, CDCl_3_) δ (ppm): 156.0 (Ar-C), 146.3 (Ar-C), 141.9 (Ar-C), 137.1 (Ar-C), 132.6 (Ar-C), 127.6 (Ar-C), 124.0 (Ar-C), 120.6 (Ar-C), 116.2 (Ar-C), 113.5 (Ar-C), 109.9 (Ar-C), 29.8 (CF_3_), 17.3 (CH_3_); ^19^F NMR (376 MHz, CDCl_3_) δ (ppm): -63.42 (s). MS (m/z): 386.04 [M+H]^+^. Anal. Calcd. for C_15_H_10_F_3_N_3_O_4_S: C, 46.76; H, 2.62; N, 10.91; Found: C, 46.72; H, 2.67; N, 10.86.

#### 2.7.9 5-Nitro-1-tosyl-2-(trifluoromethyl)-1H-benzo[d] imidazole (2ci)

Compound **2ci** was synthesized following general procedure II from 5-nitro-2-(trifluoromethyl)-1H-benzo[d]imidazole (**2c**) (0.1 g) and tosyl chloride (1.20 mmol). The formation of **2ci** was verified by TLC monitoring and purified *via* the decantation process. Yellow solid; Yield 0.08 g, 51%; R_f_: 0.82 (CH_3_OH/CHCl_3_ 1:19, UV). IR (ν, cm^-1^): 3110-3050 (aromatic C-H stretch), 2930-2860 (sp^3^ C-H stretch), 1630-1590 and 1480-1390 (aromatic C=C stretch), 1530 and 1320 (N=O stretch), 1350 (asymmetric S=O stretch), 1230-1140 (C-F stretch), 1120 (symmetric S=O stretch), 1030-1000 (C-N stretch); ^1^H NMR (400 MHz, CD_3_COCD_3_) δ (ppm): 8.66 (s, 1H), 8.28 (d, *J* = 9.0 Hz, 1H), 7.93 (d, *J* = 8.9 Hz, 1H), 7.74 (d, *J* = 7.9 Hz, 2H), 7.33 (d, *J* = 7.9 Hz, 2H), 2.37 (s, 3H); ^13^C NMR (100 MHz, CD_3_COCD_3_) δ (ppm): 144.9 (Ar-C), 144.2 (Ar-C), 142.6 (Ar-C), 140.9 (Ar-C), 138.7 (Ar-C), 129.4 (Ar-C), 126.5 (Ar-C), 119.8 (Ar-C), 117.4 (Ar-C), 116.3 (Ar-C), 114.6 (Ar-C), 20.5 (CF_3_), 8.3 (CH_3_); ^19^F NMR (376 MHz, CD_3_COCD_3_) δ (ppm): -65.25 (s). Anal. Calcd. for C_15_H_10_F_3_N_3_O_4_S: C, 46.76; H, 2.62; N, 10.91; Found: C, 46.71; H, 2.65; N, 10.89.

#### 2.7.10 5-Nitro-2-(trifluoromethyl)-1-((4-(trifluorome-thyl) phenyl)sulfonyl)-1H-benzo[d]imidazole (2cj)

Compound **2cj** was synthesized following general procedure II from 5-nitro-2-(trifluoromethyl)-1H-benzo[d] imidazole (**2c**) (0.1 g) and 4-(trifluoromethyl) benzene-1-sulfonyl chloride (1.09 mmol). The formation of **2cj** was verified by TLC monitoring and purified *via* the decantation process. Light yellow solid; Yield 0.08 g, 43%; R_f_: 0.62 (CH_3_OH/CHCl_3_ 1:19, UV). IR (ν, cm^-1^): 3100 (aromatic C-H stretch), 1600 and 1500-1430 (aromatic C=C stretch), 1520 and 1320 (N=O stretch), 1390 (asymmetric S=O stretch), 1260-1160 (C-F stretch), 1130 (symmetric S=O stretch), 1060-1010 (C-N stretch); ^1^H NMR (600 MHz, CD_3_COCD_3_) δ (ppm): 8.68 (d, *J* = 2.0 Hz, 1H), 8.34 (dd, *J* = 8.9, 2.1 Hz, 1H), 8.01 (d, *J* = 9.0 Hz, 1H), 7.81 (d, *J* = 8.0 Hz, 2H), 7.71 (d, *J* = 8.1 Hz, 2H); ^13^C NMR (100 MHz, CDCl_3_) δ (ppm): 148.2 (Ar-C), 146.1 (Ar-C), 143.9 (Ar-C), 140.4 (Ar-C), 137.3 (Ar-C), 128.0 (Ar-C), 127.5 (Ar-C), 121.6 (Ar-C), 117.9 (Ar-C), 112.8 (Ar-C), 109.1 (Ar-C), 29.8 (CF_3_), 29.5 (CF_3_); ^19^F NMR (376 MHz, CDCl_3_) δ (ppm): -63.29 (s), -63.43 (s). Anal. Calcd. for C_15_H_7_F_6_N_3_O_4_S: C, 41.01; H, 1.61; N, 9.57; Found: C, 41.04; H, 1.65; N, 9.53.

#### 2.7.11 5-Fluoro-1-((4-(trifluoromethyl)phenyl) sulfonyl)-1H-benzo[d]imidazole (3aj)

Compound **3aj** was synthesized following general procedure II from 5-fluoro-1H-benzo[d]imidazole (**3a**) (0.1 g) and 4-(trifluoromethyl)benzene-1-sulfonyl chloride (1.09 mmol). The formation of **3aj** was verified by TLC monitoring and purified *via* the decantation process. Greyish white solid; Yield 0.13 g, 53%; R_f_: 0.86 (CH_3_OH/CHCl_3_ 1:19, UV). IR (ν, cm^-1^): 3120-3050 (aromatic C-H stretch), 1600 and 1500-1430 (aromatic C=C stretch), 1390 (asymmetric S=O stretch), 1320-1140 (C-F stretch), 1120 (symmetric S=O stretch), 1060-1010 (C-N stretch); ^1^H NMR (400 MHz, CD_3_OD) δ (ppm): 9.35 (s, 1H), 8.74 (s, 1H), 8.30 (d, *J* = 7.8 Hz, 2H), 7.89 (dd, *J* = 7.6 Hz, 2H), 7.67 (d, *J* = 7.6 Hz, 1H), 7.37 (d, *J* = 7.7 Hz, 1H); ^13^C NMR (100 MHz, CD_3_OD) δ (ppm): 161.9 (Ar-C), 159.5 (Ar-C), 143.8 (Ar-C), 140.7 (Ar-C), 136.8 (Ar-C), 128.2 (Ar-C), 127.9 (Ar-C), 126.4 (Ar-C), 113.3 (Ar-C), 106.5 (Ar-C), 100.4 (Ar-C), 7.9 (CF_3_); ^19^F NMR (376 MHz, CD_3_OD) δ (ppm): -64.87 (s), -118.30 (s). MS (m/z): 345.0304 [M+H]^+^. Anal. Calcd. for C_14_H_8_F_4_N_2_O_2_S: C, 48.84; H, 2.34; N, 8.14; Found: C, 48.79; H, 2.30; N, 8.17.

#### 2.7.12 5-Fluoro-2-methyl-1-((4-(trifluoromethyl) phenyl) sulfonyl)-1H-benzo[d]imidazole (3bj)

Compound **3bj** was synthesized following general procedure II from 5-fluoro-2-methyl-1H-benzo[d] imidazole (**3b**) (0.1 g) and 4-(trifluoromethyl)benzene-1-sulfonyl chloride (1.09 mmol). The formation of **3bj** was verified by TLC monitoring and purified *via* the decantation process. White solid; Yield 0.12 g, 50%; R_f_: 0.77 (CH_3_OH/CHCl_3_ 1:19, UV). IR (ν, cm^-1^): 3120-2930 (aromatic C-H stretch), 2850-2650 (sp^3^ C-H stretch), 1640-1580 and 1460 (aromatic C=C stretch), 1320 (asymmetric S=O stretch), 1220 and 1160 (C-F stretch), 1120 (symmetric S=O stretch), 1060-1010 (C-N stretch); ^1^H NMR (400 MHz, CD_3_OD) δ (ppm): 7.94 (d, *J* = 8.1 Hz, 2H), 7.69 (t, *J* = 4.4 Hz, 2H), 7.67 (s, 1H), 7.45 (dd, *J* = 8.2, 1.9 Hz, 1H), 7.30 (td, *J* = 9.3, 2.0 Hz, 1H), 2.81 (s, 3H); ^13^C NMR (100 MHz, CD_3_OD) δ (ppm): 162.1 (Ar-C), 159.7 (Ar-C), 152.5 (Ar-C), 148.8 (Ar-C), 131.4 (Ar-C), 127.6 (Ar-C), 126.4 (Ar-C), 125.1 (Ar-C), 122.6 (Ar-C), 114.9 (Ar-C), 100.2 (Ar-C), 11.2 (CH_3_), 7.9 (CF_3_); ^19^F NMR (376 MHz, CD_3_OD) δ (ppm): -64.15 (s), -116.16 (s). Anal. Calcd. for C_15_H_10_F_4_N_2_O_2_S: C, 50.28; H, 2.81; N, 7.82; Found: C, 50.31; H, 2.78; N, 7.86.

### 2.8 MD Simulation Study

After successful docking, the GROMACS software [75] was used to perform the MD simulation for the best protein-ligand complex (**MAO-B-2cj**). The protein (**MAO-B**) was prepared in the TIP3P solvated water model using the CHARMM36 force field. Similarly, the CHARMM General Force Field (CGenFF) [76] web server was used to extract the topology information of the ligand (**2cj**) [77, 78]. The protein was set at the center-position of the simulation box within a minimum distance of 1 nm. The steepest descent method with 5000 steps was carried out for the minimization process. The system was equilibrated at NVT and NPT ensembles for 1000 ps using 0.2-time steps and 2 fs at 300 K. Finally, a 50 ns MD simulation run was executed for data collection and analysis. After the complete simulation, GROMACS modules of *gmx rms, gmx rmsf, gmx gyrate, gmx sasa,* and *gmx hbond* were used to analyze the RMSD, RMSF, Rg, SASA, and hydrogen bonds, respectively.

### 2.9 MM-PBSA Calculation

The MM-PBSA method was used to calculate the binding free energy (kJ/mol) of the **MAO-B-2cj** complex from MD simulated trajectories. The g_mmpbsa script [79] was used to calculate the binding free energy, and the method follows the general equation of:

Δ*G_Binding_* = *G_Complex_* – (*G_Protein_* + *G_Ligand_*)

Here, *G_Complex_* represents the free energy of the **MAO-B-2cj** complex, whereas, *G_Protein_* and *G_Ligand_* represent the free energies of **MAO-B** and **2cj**, respectively. In this calculation, overall binding free energy was dependent on the combined effects of SASA, polar solvation, Van der Waals, and electrostatic energies.

## 3 RESULTS AND DISCUSSION

### 3.1 Rationality for Designing Molecules

The main feature of Parkinson's disease is reduced dopamine levels in the brain caused by the death of dopaminergic neurons in the substantia nigra [80]. Monoamine oxidase B (MAO-B) is an enzyme that breaks down dopamine, and blocking it will increase dopamine levels [81]. The compound benzimidazole, which is made up of an imidazole ring fused to benzene, is recognized for its therapeutic activities, which include the inhibition of MAO-B, antioxidant activity, and anti-inflammatory activity [82]. The present study targeted a benzimidazole derivative as a potential Parkinson's disease target. Three crucial chemical structure-related characteristics, namely the ring systems, functional groups, and heavy atoms percentage, are directly linked to physicochemical characteristics and drug-like qualities. The aromatic, non-aromatic rings and the -F functional group are beneficial to CNS drugs [83, 84]. Fluorine or fluorine-containing functional groups exhibit numerous pharmacological activities [31]. Fluorine and methoxy groups are examples of substitutes that could boost MAO-B inhibitory activity [85, 86]. In medicinal chemistry, the nitro group is regarded as a special and adaptable functional group. However, the nitro group poses toxicity problems even though it has been used for treatments for a long time [35]. A methyl group can have a variety of therapeutic effects based on the drug molecules and the position of the methyl group in the molecule. It may increase the selectivity and efficacy of the drug molecules [87]. Based on the aforementioned information, the positions 1, 2, and 5 of benzimidazole substituted by nitro, fluoro, fluromethyl, methyl, phenyl, 4-methyl phenyl, 4- trifluoromethyl phenyl, and 4-methoxy phenyl group are essential for better efficacy and drug-likeness.

### 3.2 Molecular Docking

#### 3.2.1 Docking Analysis

The docking accuracy has been evaluated by calculating the RMSD. The lower the value of RMSD, the higher the accuracy of docking. A commonly accepted threshold of RMSD for successful docking is less than 2.0 Å, although it depends on ligand size. The calculated RMSD value of co-ligand 4CR, of 2C65, was 1.534 Å, which was found within the acceptable range. The RMSD values were calculated by superimposing the best-docked poses (ash color) of 4CR on the crystallographic bound conformation (green color), which is shown in Fig. (**2**). The docking protocol has been verified as it can replicate the docking posture of the co-ligand in accordance with its biological configuration in the protein-ligand complex crystal structure [88, 89].

From the ADV study, the predicted binding affinity of known MAO-B inhibitors was calculated (ranges from -6.8 to -9.6 kcal/mol), with inhibitor **VIII** having the highest value at -9.6 kcal/mol. However, the binding affinities of all hits (except **1ag**) were significantly superior to those of known inhibitors. The binding affinity of the superior hits (>10 kcal/mol) **1ai**, **1aj**, **1bh**, **1bi**, **1bj**, **1bk**, **2ai**, **2aj**, **2bi**, **2bj**, **2ci**, **2cj**, **3aj**, **3bj,** and **3bk** were -10.3, -11.0, -10.3, -11.0, -11.8, -10.6, -10.2, -11.0, -11.0, -11.7, -11.3, -11.9, -10.6, -11.5, and -10.3 kcal/mol, respectively. Table **2** displays the binding affinities of known inhibitors and newly designed compounds (**1ag-3bk**), with **2cj** having the highest affinity at -11.9 kcal/mol and **1ag** having the lowest at -7.7 kcal/mol.

With no exception, all the compounds bound in the active site in a similar fashion are shown in Fig. (**3A**). Except for known inhibitors, such as Pramipexole **(III)** and Tolcapone (**VIII)**, the inhibitors Benztropine **(II)**, Levodopa (**V**), Selegiline **(VI)**, and Rasagiline **(VII)** were bound in the active site in the comparable alignment, as shown in Fig. (**3B**). The binding poses of **VIII** (blue color) and **III** (yellow color) were slightly deviated from the other known inhibitors. The binding postures of all the selected hits were similar to known inhibitors **II**, **V**, **VI**, and **VII**. All of the hits demonstrated hydrogen bonding interactions with SER-59 (1.80 Å), TYR-60 (2.00Å), and GLY-434 (2.40Å) from close distances. The known inhibitor **V** also showed similar hydrogen bonding interaction with TYR-60 (2.40Å), SER-59 (2.20Å), and GLY-434 (2.30Å).

With the highest docking score (binding affinity -11.9 kcal/mol), compound **2cj** turned out to be the most efficient drug-candidate among the twenty molecules (Table **2**). It showed H-bonding interactions with SER-59 (2.10Å), TYR-60 (2.00Å), and TRP-388 (2.80Å). In addition to the common hydrogen bonding contacts like known inhibitors, **2cj** displayed an additional H-bonding interaction with TRP-388, as shown in Fig. (**4**). This compound also showed hydrogen-fluorine interactions with GLY-434 (2.80 and 3.10 Å) and π-π stacked interactions with TYR-398 and TYR-435. It showed π-sulfur interaction with CYS-397. Figs. (**S60** and **S61**) in supporting information, respectively, demonstrate the 3D binding poses and 2D interactions of twenty of ADV's finest hits (**1ag-3bk**), as well as the standard inhibitors **II**, **III**, **V**, **VI**, **VII,** and **VIII**. Since all of the designed MAO-B inhibitors had comparable 3D binding postures like **2cj**, 3D binding interactions of other compounds are not included here in detail.

It is evident from Table **S1** that there are interactions between the ligands and the amino acids of the protein within the active site (interacting residues of the co-ligand). All the studied benzimidazoles demonstrated interactions with the SER-59 and TYR-60 *via* conventional hydrogen bonding (H-bond). In addition, **2ak** showed H-bond interactions with TRP-388 and MET-436, and **2aj** and **2cj** with TRP-388 within the active site. On the other hand, two traditional drugs, selegiline **(VI)** and rasagiline **(VII),** demonstrated no prominent H-bond interaction within the active site (Table **S1**).

With no major exception, most of the molecules showed van der Waals, π-Sulfur, π-π T-shaped, π-π stacked and amide-π stacked interactions with interacting residues GLY-58, CYS-397, TYR-398, TYR-435, and GLY-57, respectively. For most of the compounds, alkyl interactions were displayed with VAL-294, LYS-296, and CYS-397, and π-alkyl interactions with TYR-398 within the active site (Table **S1**). These findings suggest that the studied derivatives can be utilized to treat Parkinson's disease and will be just as effective as or even more so than the reported drugs currently available in the market.

### 3.3 Analysis of Physicochemical Properties and Drug-likeness

Based on the docking analysis, twelve (**1ai-3bj**) of the twenty compounds were synthesized and possessed good docking scores, which were further analyzed for physicochemical properties, drug-likeness, and ADMET studies.

According to Lipinski's rule of five, the number of hydrogen bond donors should not exceed 5, the number of hydrogen bond acceptors should not exceed 10, the compound's molecular mass should be less than 500 Dalton, and the octanol-water partition coefficient (log P) should not exceed five. Lipinski's rule of five [90] for oral availability is not violated by any of the twelve newly synthesized compounds (**1ai-3bj**), including the two reference-drugs (**VI** and **VII**), proving that the compounds have drug-like molecular nature (Table **S2**). It is important to note that Lipinski's rule of five is crucial for rational drug design, and it has been speculated that when one of Lipinski's rule of five is violated, a given molecule will have low permeability or poor absorption [91]. Molecular weights of the benzimidazoles were under 500, and the number of hydrogen bond acceptors (NHBAs) was under 10 except for **2cj** (NHBA 11). The range of the octanol-water partition coefficient value of 12 hits was 2.49‒4.219, which is within the permitted range. The compound’s high log P value indicates its preference for octanol, a non-polar organic solvent, over polar solvent water, while a low partition coefficient suggests a stronger affinity for water. Although the molecules demonstrated no notable similarity in their molecular mass, the prediction showed similarities in TPSA and %Abs, while MR values showed diversity (Table **S2**). The molecules with a TPSA of 60 Å2 would result in good absorption (> 90% fractional absorption), whereas those with a TPSA of 140 Å2 or higher would result in poor absorption (< 10% fractional absorption) [92]. The TPSA of the benzimidazole derivatives showed greater polar surface area (in the range of 60.34 to 115.39 Å2) compared to that of the two references, selegiline (3.24 Å2) and rasagiline (12.03 Å2).

The results also showed an inverse relationship between TPSA and %Abs but no direct relationship between either TPSA and MR or % Abs and MR. The analysis of the %Abs of the twelve studied benzimidazoles and the two reference drugs (Table **S2**) showed that all the benzimidazoles possessed moderate percent absorption (ranges from 72.37 to 88.18%) in comparison with the two references, whereas selegiline displayed better percent absorption (107.88%) than rasagiline (104.85%).

Among the twelve benzimidazoles under study, the result of log S (Optimal: > -4.0 log mol/L) showed that the log S value of **2cj** and **2bj** was slightly lower than the optimal value, suggesting their solubility to be slightly lower than the others. The presence of less hydrocarbon component than the other derivatives tested in this work accounts for the comparatively high solubility of **2ai**. On the other hand, with increasing the number of electronegative fluorine atoms, solubility decreases in the case of **3aj** > **3bj** > **2cj**. The partition coefficients (log P) of the derivatives (Table **S2**) range from 2.49 to 4.219. The lipophilicity (log P) analysis revealed that practically all of the examined compounds are highly lipophilic when compared to the two reference drugs. The results of the logP values showed that among all the studied benzimidazoles, **1ai**, **2ai,** and **2bi** have optimal lipophilicity (Optimal: 0 < logP < 3), and the logP values for other benzimidazoles were marginally higher than 3. Selegiline and rasagiline, the two commercially available drugs, showed logP results of 2.183 and 1.897, respectively, indicating that they had a respectable level of membrane permeability. While logP describes lipophilicity for neutral compounds only, logD takes into account the pH dependence of ionizable molecules in aqueous solution. The computed results (Table **S3**) for the distribution coefficient (at physiological pH 7.4, *i.e.*, logD 7.4) of the benzimidazole derivatives showed that they would be more susceptible to moderate aqueous solubility with moderate lipophilicity and low metabolism in the body owing to good membrane permeability [93]. Although further clinical research is needed, the anticipated outcomes imply that oral and intestinal absorptions are feasible.

The water solubility (mg/mL) of the twelve studied derivatives was predicted using the SwissADME web tool and is tabulated in Table **S2**. The prediction depicts **2cj** is the least soluble (0.00208 mg/mL), suggesting that **2cj** plays an absorption enhancer or high absorption role albeit having low water solubility.

### 3.4 Pharmacokinetic Properties Analysis

Over 40% of identified prospective medications fail in clinical trials due to poor ADME properties [QikProp. *Schrödinger* (LLC, 2021)]. Prediction of ADME is crucial for optimizing drug development, assuring drug safety, and ultimately bringing effective and safe remedies to market.

#### 3.4.1 Absorption

We predicted Caco-2-permeability (Caco-2), P-glycoprotein (P-gp) substrate or inhibitor (P-gp sub or P-gp inh), human intestinal absorption (HIA), 20% bioavailability (F20%), and 30% bioavailability (F30%) in order to assess the absorption property of the twelve benzimidazole derivatives. These predictions are displayed in Table **S3**.

The predicted values of Caco-2-permeability of all the studied molecules are in the range from -4.459 to -4.32 cm/s, suggesting they might have excellent permeability (> -5.15 cm/s). The appreciable Caco-2-permeability (cm/s) of all the studied benzimidazoles is mainly due to the presence of a lesser number of NHBDs and NHBAs in their structures. The two reference drugs, selegiline and rasagiline, are predicted to have Caco-2-permeability values of -4.306 cm/s and -4.228 cm/s, respectively.

The ADMETlab prediction data shows that none of the studied molecules, including two references, acts as a P-gp inhibitor; rather, only selegiline, from the list (Table **S3**), acts more likely to be a P-gp substrate. On the contrary, SwissADME prediction data displays no molecule in the list to be a P-gp substrate (Table **S4**).

The predicted Human Intestinal Absorption (HIA) data of the twelve benzimidazoles and the two reference drugs are represented categorically as 0 and 1 (Table **S3**), which showed that all twelve studied molecules along with the two reference drugs have high HIA (≥ 30%). All twelve benzimidazole derivates and the reference, rasagiline, were predicted to show bioavailability greater than 20% and even greater than 30%. The reference drug, selegiline, was predicted to show low bioavailability (< 20%, Table **S3**).

#### 3.4.2 Distribution

To analyze the distribution of drug candidates, parameters, such as Plasma Protein Binding (PPB), Blood-Brain Barrier (BBB) permeability, and Volume of Distribution (VD), were also taken into consideration (Table **S3**).

The PPB of **2aj**, **2bj**, **2ci**, and **2cj** is predicted to be above 90% to bind to common blood proteins significantly, indicating that the pharmacological action of these compounds is minor (Table **S3**). On the other hand, the two references, selegiline (PPB 69.483%) and rasagiline (PPB 70.222%), moderately bind to common blood proteins and show a relatively high therapeutic index.

Using ADMETlab, it was predicted that all twelve benzimidazole derivatives and the two reference drugs would penetrate BBB (Table **S3**). However, our analysis using PreADMET webtool revealed that the BBB (≥ 1.0) can be penetrated by all (including the two references) but **2ai**, **2aj**, **2bi,** and **2bj** (Table **S4**), acknowledging these to be CNS (central nervous system)-active compounds. PreADMET prediction pointed out that the molecules, namely **2ai**, **2aj**, **2bi,** and **2bj**, containing the -NO_2_ group might not be able to penetrate BBB (< 1.0) may be due to their charged nature.

The volume of distribution (VD) of the benzimidazole derivatives was predicted to be all negative, indicating their confinement to blood and higher distribution in plasma than in tissues. Low values of VD were influenced by the hydrophilic nature of the benzimidazoles. Table **S3** also shows the most negative value of VD for **2bj** (-0.987) and the least negative value for **1bi** (-0.099), followed by **1ai** (-0.199), among all twelve benzimidazoles. The substantial tissue binding of two references, selegiline (1.08) and rasagiline (1.125), is indicated by their high VD values.

#### 3.4.3 Metabolism and Excretion

The skin permeability, log Kp, and other values predicted for all of the benzimidazole derivatives taken into consideration in the present study are in the range of -6.21 to -5.39 cm/s (Table **S4**), exhibiting low skin permeability. Except for **2bj**, **2ci,** and **2cj**, the rest of the benzimidazole derivatives, as well as the two reference drugs, are predicted to have high absorption into the gastrointestinal tract (Table **S4**).

None of the studied molecules, including two reference drugs, is predicted to interact with CYP3A4. Only the two references are predicted as CYP2D6 inhibitors. In this study, except for the two references, all the studied molecules are predicted to interact with CYP2C9 (Table **S4**). According to earlier studies, the inhibition and induction of the CYP enzymes are likely the common causes of the majority of drug interactions. Table **S4** shows that except for **2cj**, the rest of the investigated molecules, excluding the two references, exhibited good interaction with CYP2C19. On the other hand, except for **2ai** and **2bi**, the rest of the benzimidazole derivatives are predicted to be CYP1A2 inhibitors (Table **S4**). In general, the outcomes of our predictions could serve as significant inputs for future experimental research.

The human liver microsomal (HLM) stability and maximum recommended therapeutic dose (MRTD) parameters of the benzimidazole derivatives were predicted using the vNN method (Table **S4**), which revealed that all of the benzimidazole derivatives displayed prominent HLM stability. In an HLM assay, stable compounds should have a half-life (T_1/2_) of at least 30 minutes; otherwise, they are regarded as unstable. With the exception of rasagiline, all other investigated compounds, including selegiline, are active for HLM prediction (Table **S4**) and may be rapidly metabolized by the liver, reducing their effective therapeutic concentrations in the body [66]. Maximum single-day oral dosages of the twelve benzimidazoles under study range from 178 to 233 mg/day for an average adult weighing 60 kg, which is relatively high compared to the reference drugs, selegiline and rasagiline (Table **S4**).

All the studied molecules, including the two references, are predicted to possess a half-life (T_1/2_) of more than 1.5 hours (Table **S3**). All twelve benzimidazole derivatives, as well as two reference drugs, are estimated to show lower drug-clearance values (Table **S3**). Although they all show low clearance values, the benzimidazoles possess relatively lower clearance (< 1 mL/min/kg), even negative values, compared to the two reference drugs.

### 3.5 Toxicity Profile/Toxicological Endpoints Analysis

Very low or the absence of toxicity is one of the most important criteria in selecting inhibitors as a drug candidate. Herein, the organ toxicity (hepatotoxicity) and toxicological endpoints (carcinogenicity, immunotoxicity, mutagenicity, and cytotoxicity) of the twelve benzimidazole derivatives, together with two drug-references, were predicted. Table **S5** displays the anticipated descriptors. None of the compounds are immunotoxin or cytotoxic, according to the findings. Of the considered benzimidazole derivatives, the nitro (-NO_2_) group comprising benzimidazole derivatives (*i.e.*, **2ai-2cj**) demonstrated two toxicological endpoints: carcinogenicity and mutagenicity, whereas the remaining derivatives were inactive to both endpoints. However, active hepatotoxicity was present in **2aj**, **2bj**, **2ci** and **2cj**. In Table **S5**, the probability of activity is presented alongside the predicted toxicological endpoints. Although the benzimidazole derivatives under consideration possess acute toxicity as compared to the conventional drugs, they can be categorized into harmful toxic classes.

The median lethal dosage (LD_50_) values were between 17 and 1600 mg/kg (Table **S5**). Selegiline and **2ai** are classified as dangerous (Class IV) toxicity classes under the worldwide standardized method of categorization of labelling of chemicals (as specified in ProTox-3.0). The remaining compounds (Class II and III) were anticipated to be somewhat hazardous. The findings demonstrated that few of the drug-like molecules are likely to exhibit carcinogenic activities and other toxicological endpoints.

In addition to the ProTox-3.0 descriptors, the toxicity of the benzimidazole derivatives and the two reference drugs was predicted using ADMETlab descriptors, such as hERG Blockers (hERG), Human Hepatotoxicity (H-HT), AMES Mutagenicity (AMES), and Skin Sensitization (SkinSen). Table **S6** displays the findings. The numbers 1 and 0 in the category section of Table **S6** represent positive and negative, respectively, for the predicted toxicities. Except for **1bi** and **1bj**, none of the other benzimidazole derivatives or the two reference drugs were projected to be hERG blockers. However, all of them, with the exception of **3aj**, were anticipated to exhibit H-HT, and none displayed AMES mutagenicity (Table **S6**). Hepatocellular carcinoma (HCC) cells, characterized by genomic instability and intricate morphological features, often demonstrate inherent resistance to standard chemotherapeutic treatments or quickly develop resistance after an initial period of responsiveness [94]. These benzimidazole derivatives may induce hepato-carcinogenesis in the HepG2 cell line, which might lead to HCC. Furthermore, except for the two references, none of the investigated compounds were predicted to trigger skin sensitization (Table **S6**).

None of the compounds, including the two reference drugs, were found to be cytotoxic, according to the vNN predicted cytotoxicity results (Table **S6**), which is consistent with ProTox-3.0 prediction results. All of the benzimidazole derivatives were expected to be active for inducing liver injury (DILI), with the exception of the two drug-references. The mitochondrial membrane potential (MMP) parameter of the examined molecules predicted *via* the vNN method (Table **S6**) displayed that all of them were inactive for mitochondrial toxicity.

The results of the pharmacokinetic, drug-likeness, and ADMET analyses may suggest that, with few exceptions, the majority of the synthesized derivatives have potential drug-activity and biological acceptability. The best docking score held by **2cj**, which has favorable drug-like properties, may hold great promise for the treatment of Parkinson's disease. After *in vitro* and *in vivo* activity studies, these molecules might be developed into potential lead molecules for the treatment of Parkinson's disease.

### 3.6 Synthesis and Characterization of Intermediate 1a-3b and Compounds 1ai-3bj

Based on the above docking study and the physicochemical properties and drug-likeness of the twenty benzimidazole derivatives (**1ag-3bk**), we synthesized twelve of them, possessing the best docking scores and efficient drug-likeness. All the desired products **1ai-3bj** were synthesized by employing conventional methods with low to moderate yields. The percentage yields of the respective intermediates and final products vary with their overall electron donor-ability as well as nucleophilicity.

The yields of the intermediates (**1a–3b**) in Table **3** indicate that the percentage yield increases as the starting material's electron donor-ability increases. As a result, the percentage yields of the intermediates generated from starting materials **2** (having a -NO_2_ group) and **3** (having a -F group) are significantly lower than those derived from **1** (having a -CH_3_ group). This may be due to the fact that the electron-withdrawing -NO_2_ group in the aromatic system lowers the electron donating-ability of the N-atom of amine (-NH_2_) group *via* −M (negative mesomeric) effect both in the starting material and in the case of intermediate. Similarly, the -F group in the ring lowers the electron-donating ability or basicity as well as nucleophilicity of the donor N-atom through −I (negative inductive) effect. On the other hand, the -CH_3_ group in the ring provides much higher yields of the intermediates because its +I (positive inductive) effect induces greater electron-donating power on the donor N-atom.

The relative yields of the products **1ai-3bj** may be explained by the ease of chloride (Cl^-^) ion removal from the respective sulfonyl chlorides as well as the overall stability of sulfonyl derivatives varying R^3^ groups. Phenyl ring adjoint to sulfonyl chloride somewhat stabilizes the S_N_1 intermediate, *i.e.*, cation on S-atom formed after the removal of Cl^−^ ion through resonance. Depending upon the varying functional groups on phenyl (R^3^) moiety, the yield of the respective sulfonyl derivatives varies with their potential resonance stabilizing ability. Stabilizing the intermediate through the effective inductive (+I) effect of the -CH_3_ group promotes a product with good yield. In contrast, -the CF_3_ group possesses the −I effect to destabilize the intermediate, resulting in a low yield of the corresponding sulfonyl derivatives.

Compared to the other compounds listed in Table **4**, compound **1ai** exhibits a moderately high yield (66%). Changing the R^2^ group from -H to -CH_3_ reduces the percentage yield of the final product. Variation in the R^3^ group reflects the percentage yield elevation with the introduction of an electron-donating group -CH_3_ at the para-position of the phenyl ring, whilst the introduction of electron-withdrawing -CF_3_ groups in the ring lowers the final yield.

Melting point (m.p.) and ^1^H NMR data for all the intermediates (**1a–3b**) corresponded well with the published literature [54, 68-71]. Following the analysis of the mass spectra data, IR, ^1^H NMR, ^13^C NMR, and ^19^F NMR (in certain cases), the formation of new molecules, **1ai-3bj**, was established. The ^1^H NMR and ^13^C NMR of compounds **1bi**, **2ai**, and **2bi** were consistent with the findings outlined in the literature [72-74]. The characteristic S=O bond stretching frequency for all sulfonyl derivatives of benzimidazole came within the range of 1390 to 1310 cm^-1^ for asymmetric stretching and 1170-1100 cm^-1^ for symmetric stretching in the IR region. Apart from that, the -NO_2_ group containing moieties (**2ai-2cj**) displayed asymmetric N=O stretching in the range of 1550 to 1490 cm^-1^ and symmetric stretching at 1355-1315 cm^-1^ IR frequency. The ^1^H NMR data of sulfonyl derivatives of 5-methyl-substituted benzimidazole, **1ai** and **1aj**, showed six sets, while **1bi** and **1bj** displayed five sets of peaks with different multiplicities, in the aromatic proton region, *i.e.*, δ 9.50-7.00 ppm, and - CH_3_ singlet peaks in the aliphatic region, *i.e.*, δ 2.80-2.20 ppm, confirming their synthesis. Moreover, ^1^H NMR spectra of sulfonyl derivatives of 5-nitro-substituted benzimidazole suggest that **2ai** and **2aj** displayed six sets, while **2bi**, **2bj**, **2ci** and **2cj** showed five sets of peaks with different multiplicities in the aromatic proton region, *i.e.*, δ 9.00-7.00 ppm, and - CH_3_ singlet peaks in the aliphatic region, *i.e.*, δ 2.90-2.20 ppm with exceptions of **2aj** and **2cj** showing no singlet peak (-CH_3_) in the aliphatic region. Similarly, sulfonyl derivatives of 5-fluoro-substituted benzimidazole, **3aj** and **3bj**, showed corresponding aromatic peaks in the aforementioned region and aliphatic -CH_3_ singlet peak with an exception of **3aj** displaying no such signal in 1H NMR spectra. The ^13^C NMR data of compounds **1ai-3bj** were also in accordance with their corresponding structure. The presence of three peaks for **1bi**, **1bj**; two peaks for **1ai**, **1aj**, **2bi**, **2bj**, **2ci**, **2cj**, **3bj**; and one peak for **2ai**, **2aj**, **3aj** in the aliphatic region, *i.e.*, δ 30-8 ppm, of ^13^C NMR confirms the synthesis of the compounds. The occurrence of singlet peak(s) in ^19^F NMR data within the range of δ -63.00 to -65.00 verified the presence of -CF3 group(s) in the benzimidazole moieties (**1aj**, **1bj**, **2aj**, **2bj**, **2ci**, **2cj**, **3aj**, **3bj**). Moreover, the presence of fluorine (-F) group in **3aj** and **3bj** was established by the occurrence of singlet peak at δ -118.30 and -116.16 ppm, respectively. The formation of compounds (**1ai**, **1bi**, **1bj**, **2ai**, **2aj**, **2bi**, **2bj**, and **3aj**) was also validated from their corresponding molecular ion peaks [M+H]^+^ in the provided mass spectral data (Supplemental Materials).

### 3.7 Molecular Dynamics (MD) Simulation Analysis

**Fig. (5):**
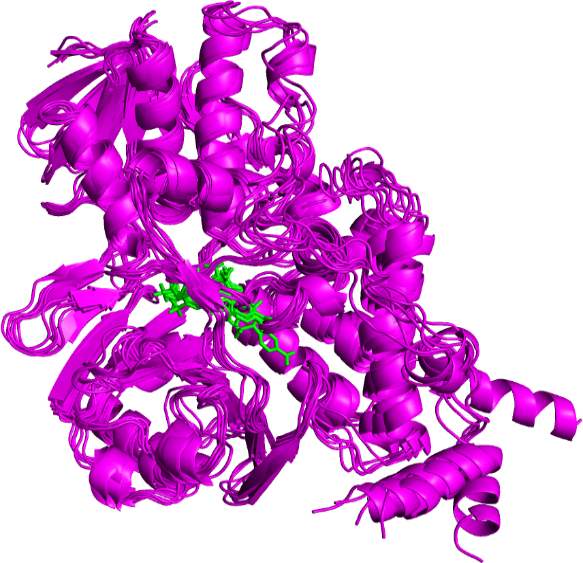
Alignment of different simulated structures (MAO-B-2cj) during 50 ns simulation (snapshots duration 10 ns).

Molecular docking fails to account for conformational changes in receptors and ligands, treating them as rigid. However, MD simulation overcomes this limitation by considering the dynamic nature of the protein-ligand complex. This method allows the exploration of structural changes in this complex over a certain period of time, revealing valuable information about their stability and flexibility during interaction [95]. Here, one best-docked complex (**MAO-B-2cj**) from docking output was subjected to a 50 ns MD simulation run, and several MD simulation analyses were performed (Fig. **5**).

**Fig. (6):**
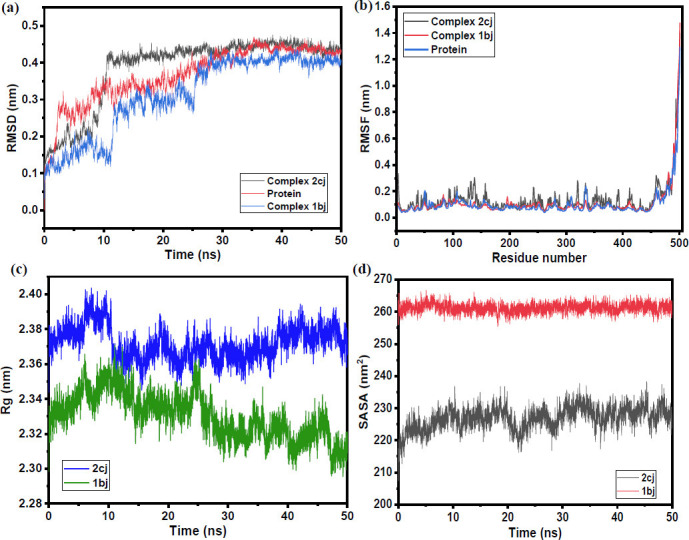
MD simulation plots including (**a**) RMSD, (**b**) RMSF of **MAO-B-2cj**, **MAO-B-1bj**, complex and MAO-B alone; (**c**) Rg, and (**d**) SASA of **MAO-B-2cj** and **MAO-B-1bj** complex.

First, the root mean square deviation (RMSD) plot is shown in Fig. (**6a**), which is an important parameter to evaluate the structural stability of the **MAO-B-2cj** complex. Generally, a lower RMSD value indicates a stable structure throughout the simulation [96]. It has been observed that the RMSD of the **MAO-B-2cj** complex was stabilized and equilibrated after 15 ns (Fig. **6a**). After that, the RMSD line of the complex remained almost consistent for the entire 50 ns. Up to 10 ns, the RMSD of **2cj**-bound protein was lower than the apo-protein, but there were no changes in the RMSD of **2cj**-bound protein after 30 ns in comparison to apo-protein. The average RMSD value of this complex was found to be 0.39 nm. Second, root mean square fluctuation (RMSF) was carried out to gain insight into the local dynamics and flexibility of the amino acid residues [97] of the **MAO-B** target (Fig. **6b**). The RMSF of **2cj**-bound amino acid residues changed to some extent in comparison to apo-protein, but RMSF values were lower than 3.0Å. The RMSF variations following binding were insignificant. As shown in the figure, the important amino acid residues at the binding site showed lower fluctuations upon binding of **2cj**. Overall, the complex has lower amino acid fluctuations, which actually supports the previous RMSD analysis.

Third, the radius of gyration (Rg) is another parameter to validate the binding-induced conformational changes of the simulated complex. It is actually used to monitor the overall compactness of the docked complex. So, a lower Rg value indicates a more compact structure by considering better protein-ligand interactions [98]. In this study, the average Rg between **MAO-B** and **2cj** was found to be 2.37 nm (Fig. **6c**). Fourth, the surface area of the protein target for the binding of the ligand is measured through another important parameter called solvent-accessible surface area (SASA). An increased value of this parameter may indicate easier binding affinity of the ligands towards the protein's active surface [99]. In this study, the average SASA value of the **MAO-B** receptor with **2cj** ligand was found to be 226.91 nm2 (Fig. **6d**). On the other hand, hydrogen bonding analysis was also carried out (Fig. **S62**) to validate the simulated results with docking results (protein-ligand interaction). In molecular docking, four hydrogen bonding interactions of **2cj** with **MAO-B** were validated with almost three hydrogen bonds of the simulated complex during 50 ns.

Additionally, at 50 ns, MD simulation investigations were performed for the second-best docked complex (**MAO-B-1bj**). Fig. (**6a**) displays the RMSD plots against simulation time. The modest fluctuations signify the achievement of a stable conformation and vice versa. However, the RMSD plots of the **1bj** complex showed a higher deviation up to 30 ns than the **2cj** complex's; two large deviations were observed in the RMSD plot, one around 12 ns and the other around 25 ns, indicating a greater stability of **MAO-B-2cj** complex than **MAO-B-1bj** complex during simulation time. Therefore, from the above molecular dynamics (MD) simulation of both protein-ligand complexes and apo-protein, there was a sharp increase in RMSD after 10 ns, indicating that a protein may undergo a major conformational change after 10 ns, resulting in a noticeable structural change. However, the RMSD change in both complexes was less than 3.0 Å, even though for globular protein, the permissible average RMSD value was ≤ 3.0Å [100].

The individual residue flexibility, or the amount that a specific residue fluctuates, is calculated by using the Root-Mean-Square-Fluctuations (RMSF). To evaluate the flexibility of the complexes, (Fig. **6b**) shows the fluctuations of each amino acid residue as a function of residue number, as supplied by the RMSF plots. Upon comparison, the RMSF values of the two complexes were found to be comparable, suggesting a crucial balance between stability and flexibility for their respective operations. Higher Rg values for the MAO-B-2cj complex (Fig. **6c**), compared to that of **1bj**, indicate better protein-ligand interactions in the 2cj complex than in the **1bj**. The higher SASA value of the **1bj** complex indicates easier binding affinity of the ligands towards the protein active site surface (Fig. **6d**). Based on molecular docking and MD simulation results, the binding free energy of the **2cj** within the receptor was calculated.

### 3.8 Molecular Mechanic/Poisson-Boltzmann Surface (MM-PBSA) Analysis

It is a useful method to predict the binding free energy of the ligand within the receptor in a dynamic environment. The method involves the calculation of various contributing energy parameters, such as van der Waals, electrostatic, polar solvation, and SASA, to the final binding free energy (kJ/mol) [101]. In this study, the binding free energy from 50 ns MD simulation trajectories of the **MAO-B-2cj** complex was calculated, as mentioned in Table **5**.

**Fig. (7):**
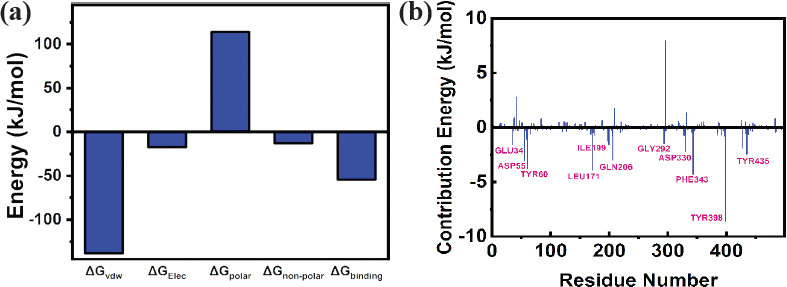
(**a**) MM-PBSA calculated energies and (**b**) Contribution of each amino acid residues of selected complex (**MAO-B-2cj**).

In this study, the complex of **2cj** with the receptor demonstrated a binding free energy of -54.266 ± 0.985 kJ/mol. In addition, the contribution of each individual amino acid residue to the binding free energy of inhibitors and selected hit complex was also examined. It provides an in-depth analysis of the contribution of significant amino acids to the binding free energy of inhibitors (Fig. **7a**). The amino acid residues GLU34 (-1.5725 kJ/mol), ASP55 (-3.0733 kJ/mol), TYR60 (-3.7774 kJ/mol), LEU171 (-3.9265 kJ/mol), ILE199 (-1.5896 kJ/mol), GLN206 (-3.0214 kJ/mol), GLY292 (-1.484 kJ/mol), ASP330 (-2.2581 kJ/mol), PHE343 (-4.3272 kJ/mol), TYR398 (-8.6136 kJ/mol), and TYR435 (-2.4542 kJ/mol) were found to have more contribution in the **MAO-B-2cj** complex (Fig. **7b**).

## CONCLUSION

The 5-substituted 1-alkyl or 1-aryl sulfonyl derivatives of benzimidazole (**1ai-3bj**) have a wide range of pharmacological and medicinal applications. Due to their numerous bioactivities, benzimidazole derivatives are significant in medicinal chemistry. In the present study, the synthesized benzimidazole derivatives demonstrated strong binding affinity to Parkinson's disease (PD)-causing MAO-B protein strain 2C65 in comparison to known inhibitors. Except for 5-methyl-1-(methylsulfonyl)-1H-benzo[d]imidazole (**1ag**), the newly designed molecules showed significantly superior binding scores to those of known inhibitors. When compared to other derivatives and widely available anti-PD drugs, 5-nitro-2-(trifluoromethyl)-1-((4-(trifluoromethyl) phenyl) sulfonyl)-1H-benzo[d]imidazole (**2cj**) demonstrated the superior docking results in ADV. The BBB-permeable (as anticipated by ADMETlab and PreADMET) CNS-active benzimidazole derivative **2cj** demonstrated substantially greater packing efficiency in interacting with the targeted protein than selegiline and rasagiline. The ADMET analysis predicted that all the 5-substituted 1-alkyl or 1-aryl sulfonyl derivatives of benzimidazoles would also gain sufficient drug-like properties with few exceptions. The MD simulation analysis confirmed the stable dynamic behavior of the best-docked complex. The docked result of the best complex was also validated through good MM-PBSA binding free energy (kJ/mol). However, before serving their intended function, these compounds must undergo *in vivo* and *in vitro* application assessments as well as numerous clinical trials. This raises interest in further research since it indicates that the synthesized compounds have a variety of characteristics and possible biological applications. These compounds may be further developed into more potential drugs after *in vitro* and *in vivo* studies.

## Figures and Tables

**Fig. (3):**
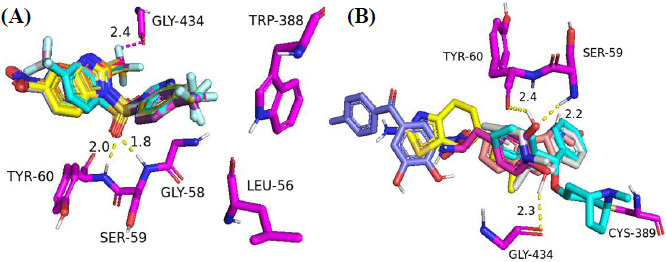
Hydrogen bonding interactions of hits (**A**) and known inhibitors (**B**) with the active site residues. Binding poses of all selected hits superimposed (**A**) in the active site and binding poses of known inhibitors superimposed in the same active site (**B**).

**Fig. (4):**
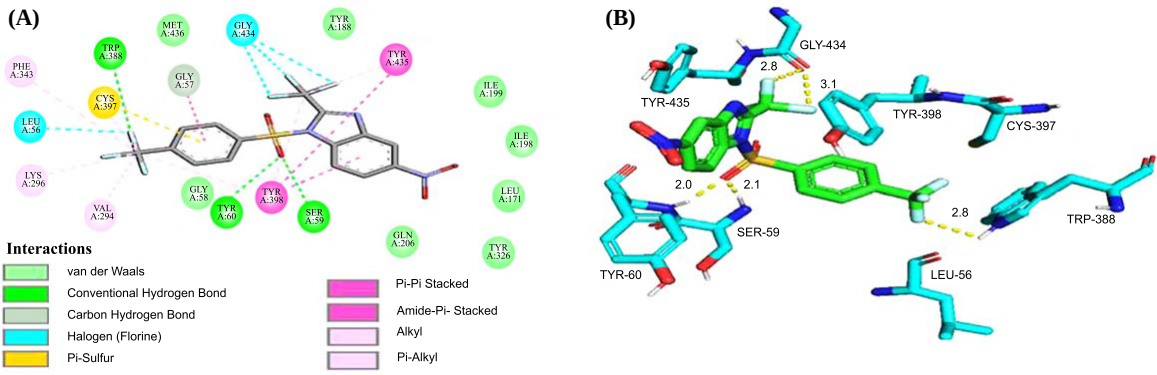
The docking poses of best-hit **2cj**, **A**) 2D ligand interactions like hydrogen bond donor and hydrogen bond acceptor. **B**) 3D ligand interaction diagram of hydrogen bond donor and hydrogen bond acceptor.

**Table 1:** List of some market-available anti-Parkinsonian drugs (data collected from the Drugbank Database).

**Name**	**Chemical Structure**	**Classification**	**Solubility (mg. mL-1)**	**Half-life (h)**	**Refs.**
Trihexyphenidyl I (MW 301.47)	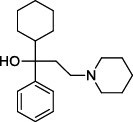	Anti-cholinergic	0.00314	2.9-3.5	[15]
Benztropine II (MW 307.43)	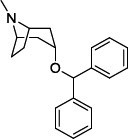	Anti-cholinergic	0.00121	~36	[16]
Pramipexole III (MW 211.33)	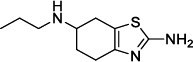	Dopamine receptor agonist	0.14	8.5-12	[17]
Rotigotine IV (MW 315.48)	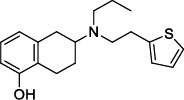	Dopamine receptor agonist	0.00904	3	[18]
Levodopa V (MW 197.19)	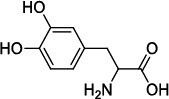	Dopamine replacement agent	3.3	2.3	[19]
Selegiline VI (MW 187.29)	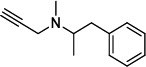	MAO inhibitor	0.0254	1.2-2	[20]
Rasagiline VII (MW 171.24)	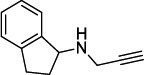	MAO inhibitor	0.0249	3	[21]
Tolcapone VIII (MW 273.24)	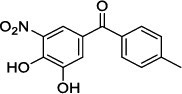	Catechol-o-methyltransferase inhibitor	0.0569	2-3.5	[22]
Carbidopa IX (MW 226.23)	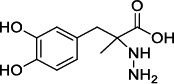	Dopa decarboxylase inhibitor	3.73	1.78	[23]
Amantadine X (MW 151.25)	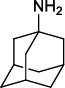	Anti-glutamatergic	0.0846	10-14	[24]

**Note:** [14].

**Table 2:** Compounds with their binding affinity predicted by AutoDock Vina (ADV).

**Ligand**	**ADV score#**	**Ligand**	**ADV score#**
**1ag**	-7.7	**2bj**	-11.7
**1ah**	-9.8	**2ci**	-11.3
**1ai**	-10.3	**2cj**	-11.9
**1aj**	-11.0	**3aj**	-10.6
**1ak**	-9.9	**3ak**	-9.7
**1bh**	-10.3	**3bj**	-11.5
**1bi**	-11.0	**3bk**	-10.3
**1bj**	-11.8	**II**	-9.2
**1bk**	-10.6	**III**	-6.8
**2ai**	-10.2	**V**	-7.1
**2aj**	-11.0	**VI**	-7.3
**2ak**	-9.8	**VII**	-8.0
**2bi**	-11.0	**VIII**	-9.6

**Note:** #Binding affinity (kcal/mol).

**Table 3:** Isolated yields of compounds 1a-3b.

**Entry**	**R^1^**	**R^2^**	**Reaction Condition**	**Compound**	**Yield (%)**
1	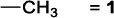	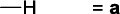	HCOOH, reflux, 4h	**1a**	74
2		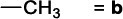	CH_3_COOH, HCl, reflux, 12h	**1b**	84
3	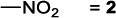	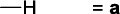	HCOOH, reflux, 4h	**2a**	62
4		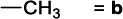	CH_3_COOH, HCl, reflux, 12h	**2b**	71
5		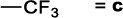	CF_3_COOH, HCl, reflux, 12h	**2c**	55
6	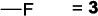	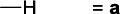	HCOOH, reflux, 4h	**3a**	71
7		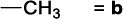	CH_3_COOH, HCl, reflux, 12h	**3b**	71

**Table 4:** Isolated yields of compound 1ai-3bj.

**Entry**	**R^1^**	**R^2^**	**R^3^**	**Compound**	**Yield (%)**
1	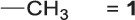	hydrogen	4-methyl phenyl	**1ai**	66
2			4-trifluoromethyl phenyl	**1aj**	53
3		methyl	4-methyl phenyl	**1bi**	61
4			4-trifluoromethyl phenyl	**1bj**	47
5	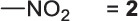	hydrogen	4-methyl phenyl	**2ai**	55
6			4-trifluoromethyl phenyl	**2aj**	44
7		methyl	4-methyl phenyl	**2bi**	54
8			4-trifluoromethyl phenyl	**2bj**	45
9		trifluoromethyl	4-methoxy phenyl	**2ci**	51
10			4-trifluoromethyl phenyl	**2cj**	43
11	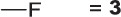	hydrogen	4-trifluoromethyl phenyl	**3aj**	53
12		methyl	4-trifluoromethyl phenyl	**3bj**	50

**Table 5:** MM-PBSA binding free energy of selected complex (MAO-B-2cj).

Van der Waals energy	-138.107 ± 1.466 kJ/mol
Electrostatic energy	-17.211 ± 0.198 kJ/mol
Polar solvation energy	+ 113.736 ± 0.957 kJ/mol
SASA energy	-12.672 ± 0.134 kJ/mol
Binding free energy	-54.266 ± 0.985 kJ/mol

## Data Availability

The data and supportive information are available within the article.

## LIST OF ABBREVIATIONS

CSF: Cerebrospinal Fluid
TLC: Thin Layer Chromatography
GI: Gastrointestinal Tract
TFA: Trifluoroacetic Acid
HCC: Hepatocellular Carcinoma
MD: Molecular Dynamics
RMSF: Root-Mean-Square-Fluctuations
