# Supplementary material for: Design, Synthesis, Molecular Docking, Pharmacokinetic Properties, and Molecular Dynamics Simulation of Sulfonyl Derivatives of Benzimidazole against Parkinson’s Disease
Source: Curr Med Chem. 2024 Oct 24;33(5):1096–119. doi: 10.2174/0109298673337912241007120510 (PMC13223459; doi:10.2174/0109298673337912241007120510)
Supplement: Supplementary file 1 [file CMC-33-5-1096_SD1.pdf]

Supplementary Material

Design, Synthesis, Molecular Docking, Pharmacokinetic Properties, and Molecular Dynamics Simulation of Sulfonyl Derivatives of Benzimidazole against Parkinson's Disease

Subarna Roy[1,2], Subhankar Basak[1], Shristi Roy[1], Paromita Dey[1], Hema Barman[1], Bhagat Singh[3], Kaushik Sarkar[4], Subhadeep Sen[1], Rajesh Kumar Das[4], Sudhan Debnath[5,*] and Goutam Biswas[1,*]

[1]Department of Chemistry, Cooch Behar Panchanan Barma University, Cooch Behar, West Bengal, 736101, India; [2]Department of Chemistry, Indian Institute of Technology Indore, Khandwa Road, Simrol, Madhya Pradesh, 453552, India; [3]Department of Chemistry and Biochemistry, University of North Carolina at Greensboro, Greensboro, North Carolina 27402, United States; [4]Department of Chemistry, University of North Bengal, Darjeeling, West Bengal, 734013, India; [5]Department of Chemistry, Netaji Subhash Mahavidyalaya, Udaipur, Tripura, 799114, India

Supplementary Information:

Contents	Page No.
➢ Names and Structures of the Designed Compounds (**1a-3b, 1ag-3bk**)	*S2-S6*
➢ Spectral Data of the Synthesized Intermediates (**1a-3b**)	*S7-S10*
➢ Spectral Data of the Synthesized Derivatives (**1ai-3bj**)	*S10-S36*
➢ Analysis of Docking Modes of **1ag-3bk**	*S36-S56*
➢ Analysis of MD Simulation of MAO-B-**2cj** complex	*S57*
➢ Analysis of Physicochemical properties and Drug-likeness	*S58*
➢ Pharmacokinetic properties Analysis	*S59-S60*
➢ Toxicity profile/Toxicological endpoints Analysis	*S61-S62*

Supplemental Materials

Names and Structures of the Designed Compounds (1a-3b, 1ag-3bk):

Compound	IUPAC Nomenclature	Structure
**1a**	5-methyl-1H-benzo[d]imidazole	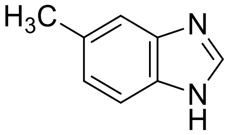
**1b**	2,5-dimethyl-1H-benzo[d]imidazole	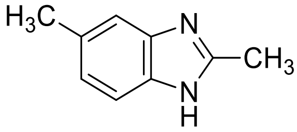
**2a**	5-nitro-1H-benzo[d]imidazole	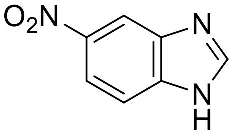
**2b**	2-methyl-5-nitro-1H-benzo[d]imidazole	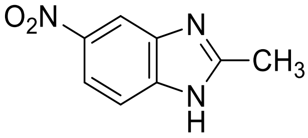
**2c**	5-nitro-2-(trifluoromethyl)-1H-benzo[d]imidazole	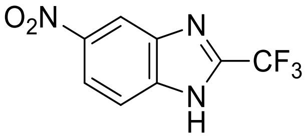
**3a**	5-fluoro-1H-benzo[d]imidazole	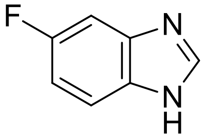
**3b**	5-fluoro-2-methyl-1H-benzo[d]imidazole	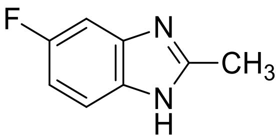

**1ag**	5-methyl-1-(methylsulfonyl)-1H-benzo[d]imidazole	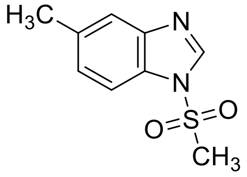
**1ah**	5-methyl-1-(phenylsulfonyl)-1H-benzo[d]imidazole	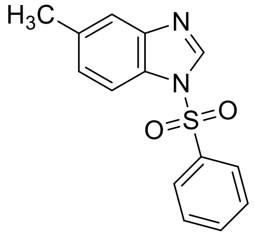
**1ai**	5-methyl-1-tosyl-1H-benzo[d]imidazole	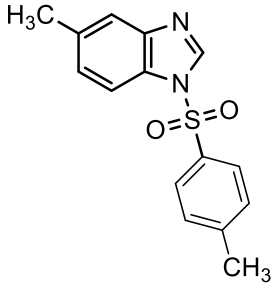
**1aj**	5-methyl-1-((4-(trifluoromethyl)phenyl)sulfonyl)-1H-benzo[d]imidazole	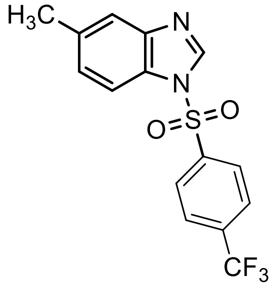
**1ak**	1-((4-methoxyphenyl)sulfonyl)-5-methyl-1H-benzo[d]imidazole	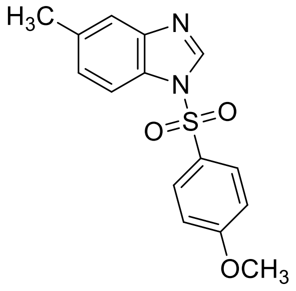
**1bh**	2,5-dimethyl-1-(phenylsulfonyl)-1H-benzo[d]imidazole	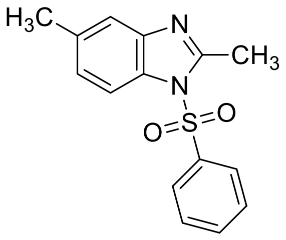

**1bi**	2,5-dimethyl-1-tosyl-1H-benzo[d]imidazole	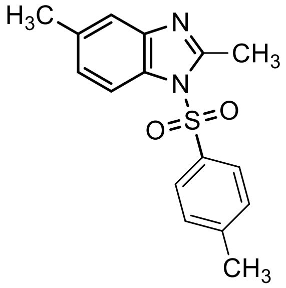
**1bj**	2,5-dimethyl-1-((4-(trifluoromethyl)phenyl)sulfonyl)-1H-benzo[d]imidazole	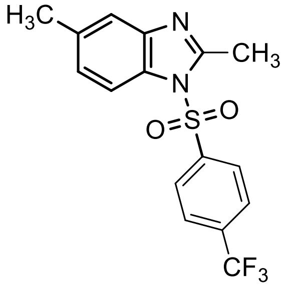
**1bk**	1-((4-methoxyphenyl)sulfonyl)-2,5-dimethyl-1H-benzo[d]imidazole	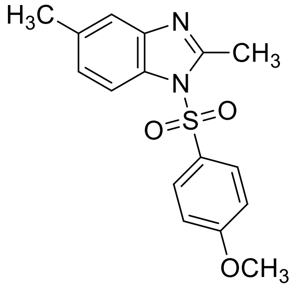
**2ai**	5-nitro-1-tosyl-1H-benzo[d]imidazole	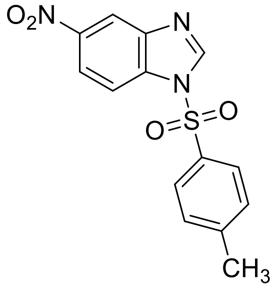
**2aj**	5-nitro-1-((4-(trifluoromethyl)phenyl)sulfonyl)-1H-benzo[d]imidazole	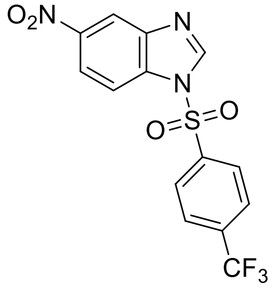

**2ak**	1-((4-methoxyphenyl)sulfonyl)-5-nitro-1H-benzo[d]imidazole	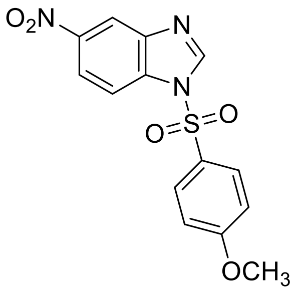
**2bi**	2-methyl-5-nitro-1-tosyl-1H-benzo[d]imidazole	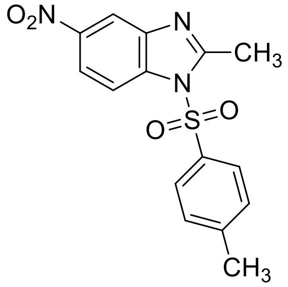
**2bj**	2-methyl-5-nitro-1-((4-(trifluoromethyl)phenyl)sulfonyl)-1H-benzo[d]imidazole	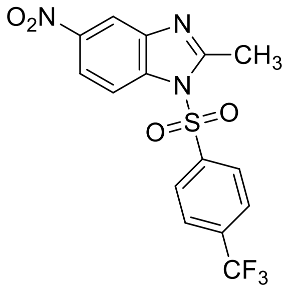
**2ci**	5-nitro-1-tosyl-2-(trifluoromethyl)-1H-benzo[d]imidazole	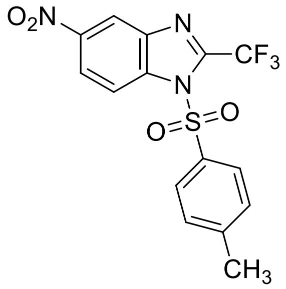
**2cj**	5-nitro-2-(trifluoromethyl)-1-((4-(trifluoromethyl)phenyl)sulfonyl)-1H-benzo[d]imidazole	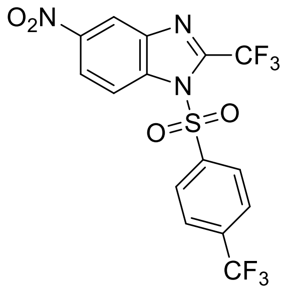

**3aj**	5-fluoro-1-((4-(trifluoromethyl)phenyl)sulfonyl)-1H-benzo[d]imidazole	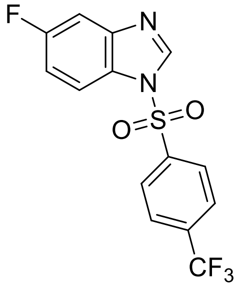
**3ak**	5-fluoro-1-((4-methoxyphenyl)sulfonyl)-1H-benzo[d]imidazole	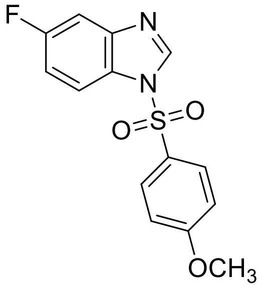
**3bj**	5-fluoro-2-methyl-1-((4-(trifluoromethyl)phenyl)sulfonyl)-1H-benzo[d]imidazole	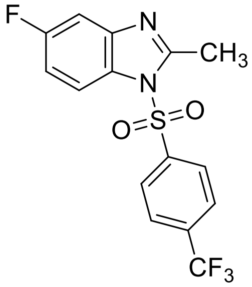
**3bk**	5-fluoro-1-((4-methoxyphenyl)sulfonyl)-2-methyl-1H-benzo[d]imidazole	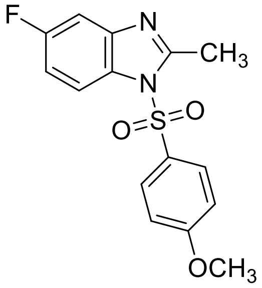

Spectral Data of the Synthesized Intermediates (1a-3b):

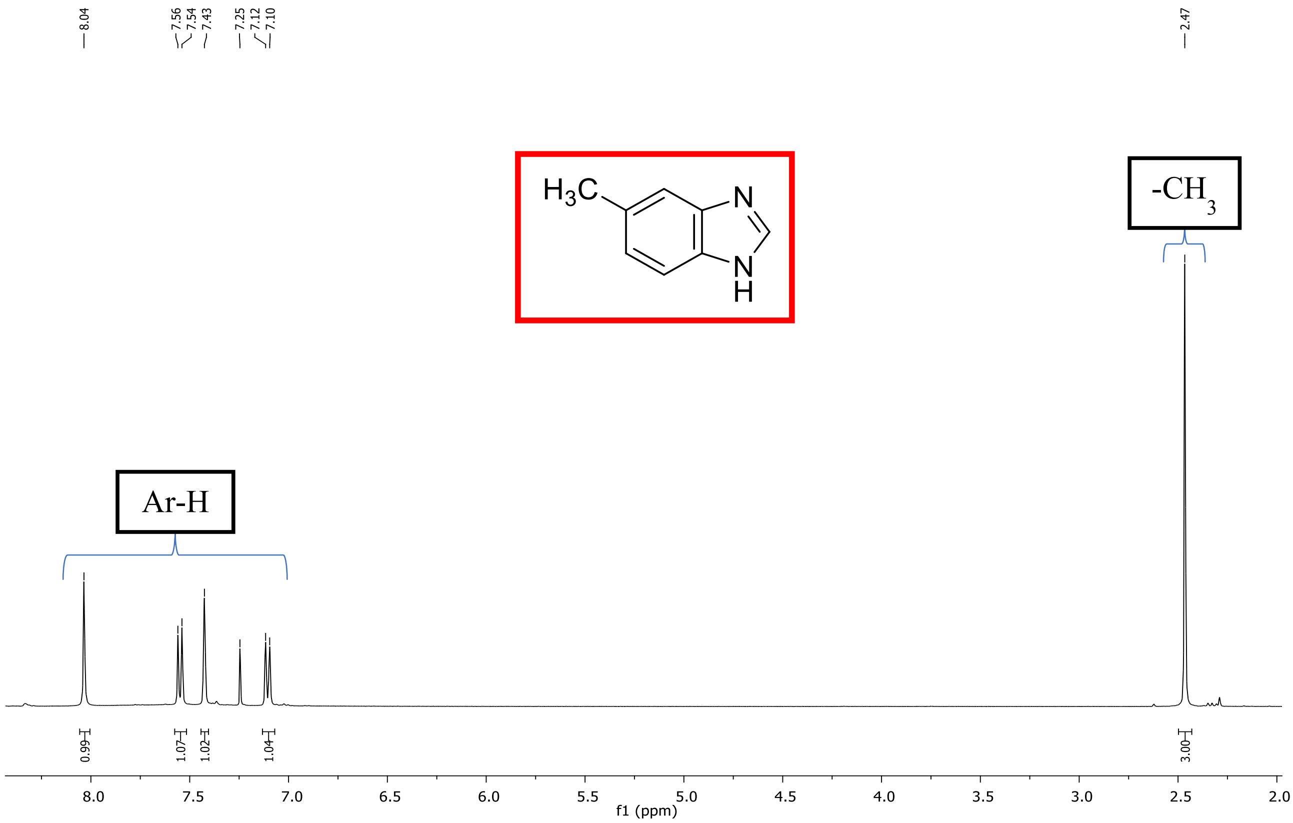

Fig S1: ^{1}H NMR Spectrum of compound **1a**.

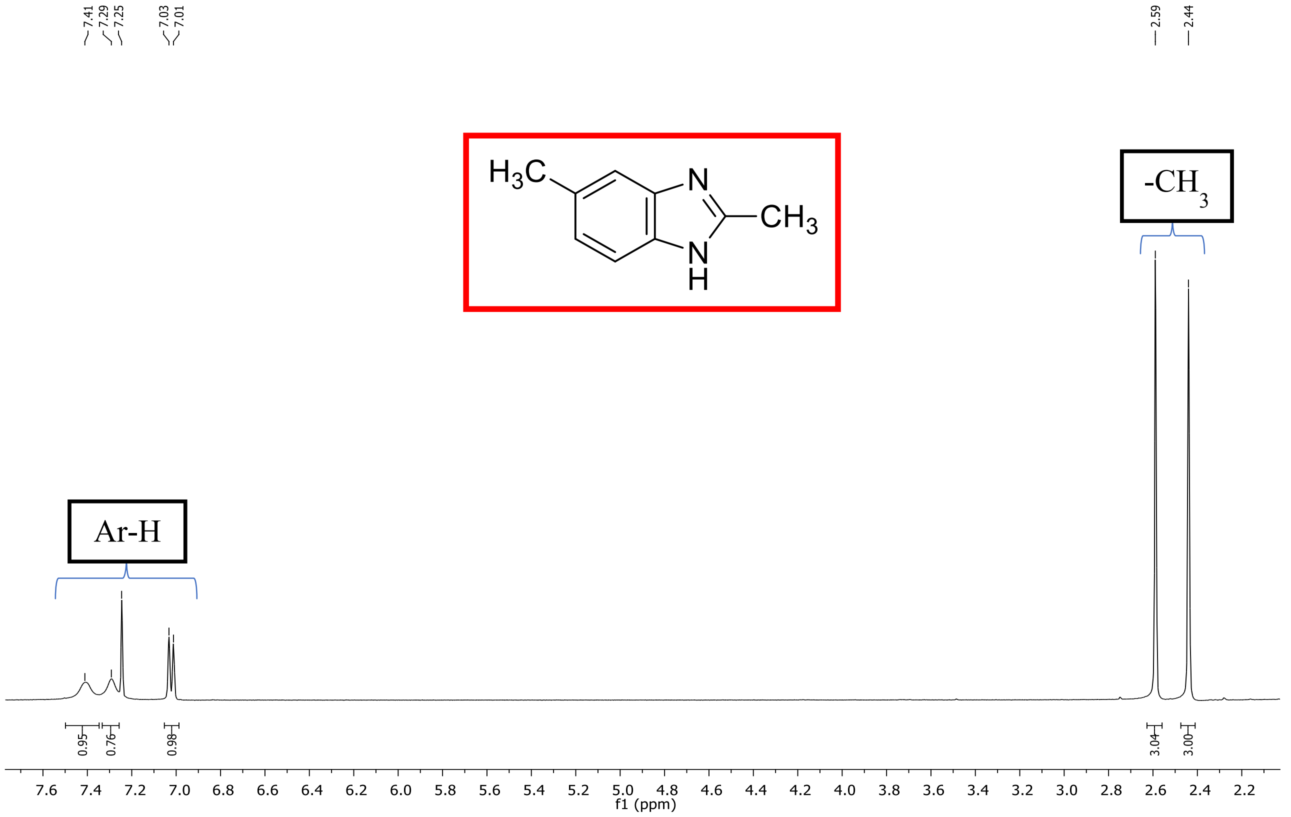

Fig S2: ^{1}H NMR Spectrum of compound **1b**.

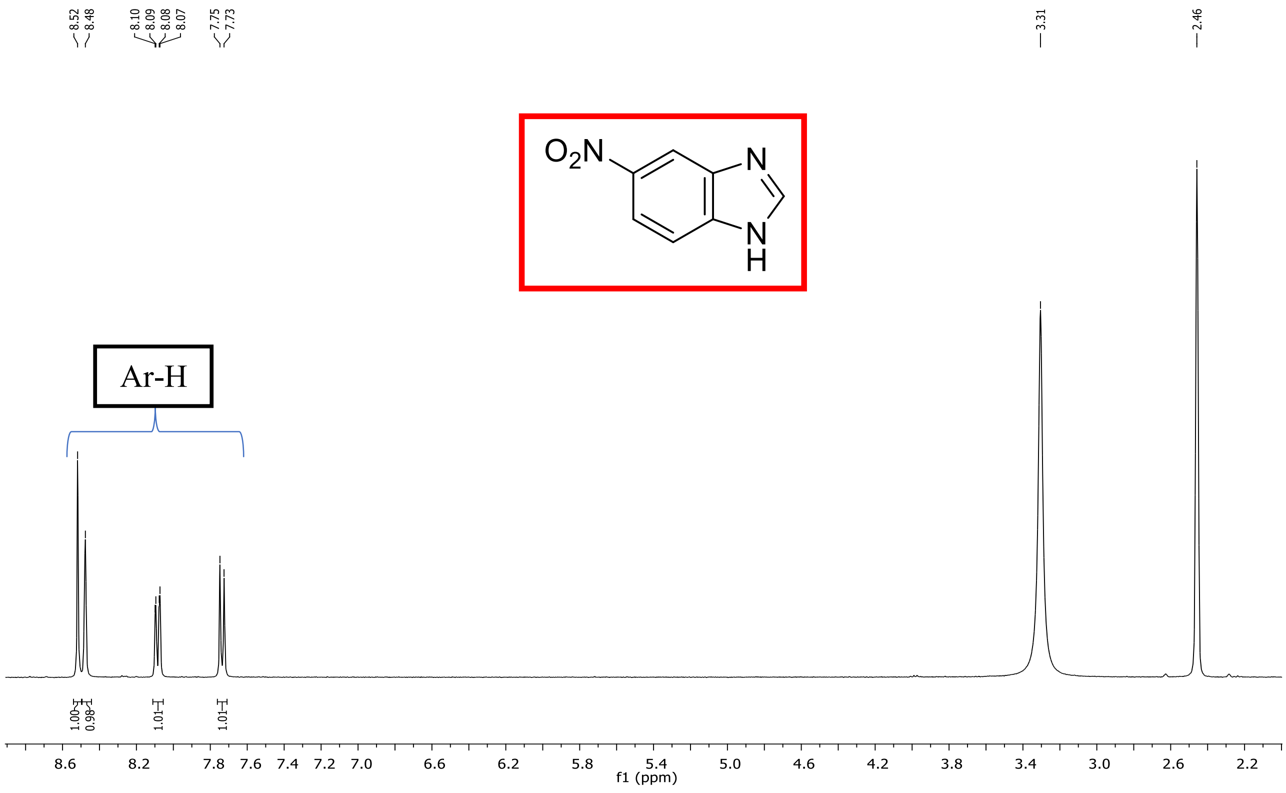

Fig S3: ^{1}H NMR Spectrum of compound **2a**.

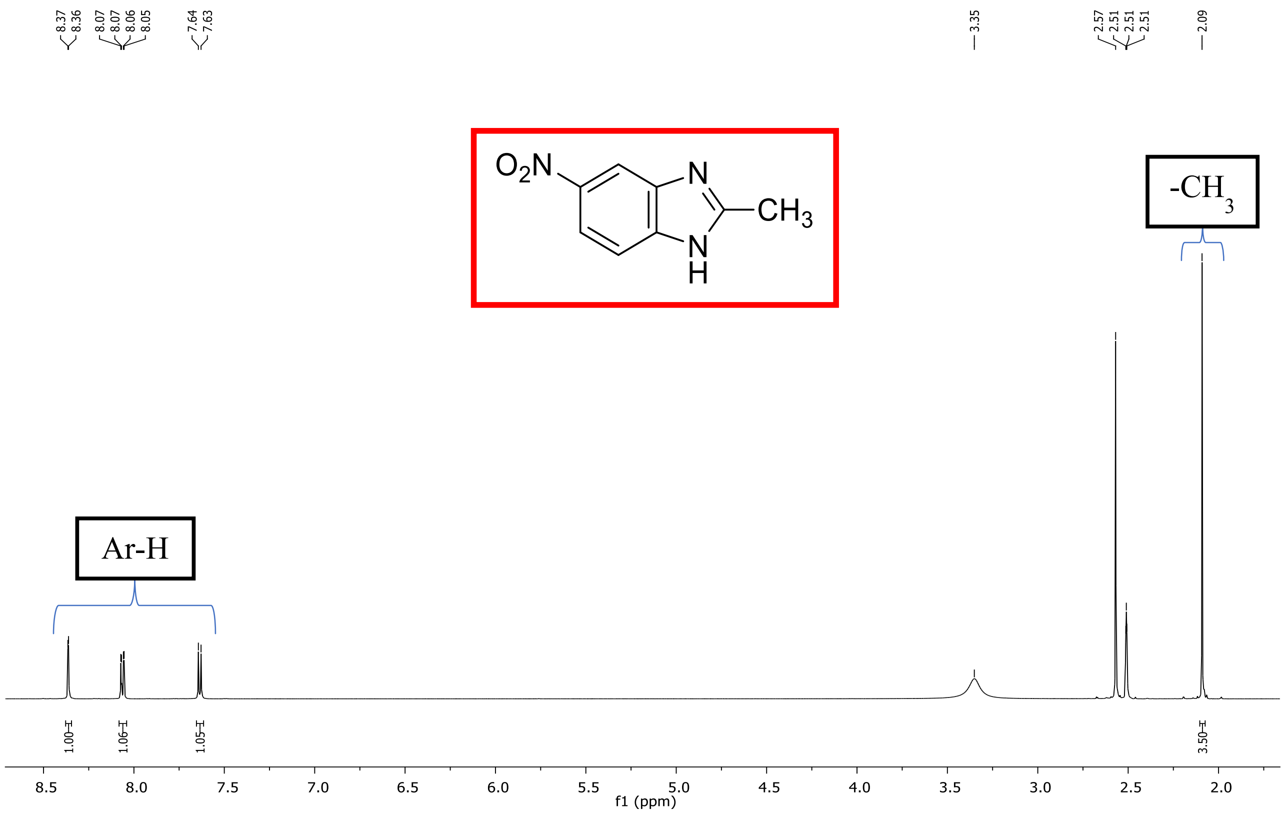

Fig S4: ^{1}H NMR Spectrum of compound **2b**.

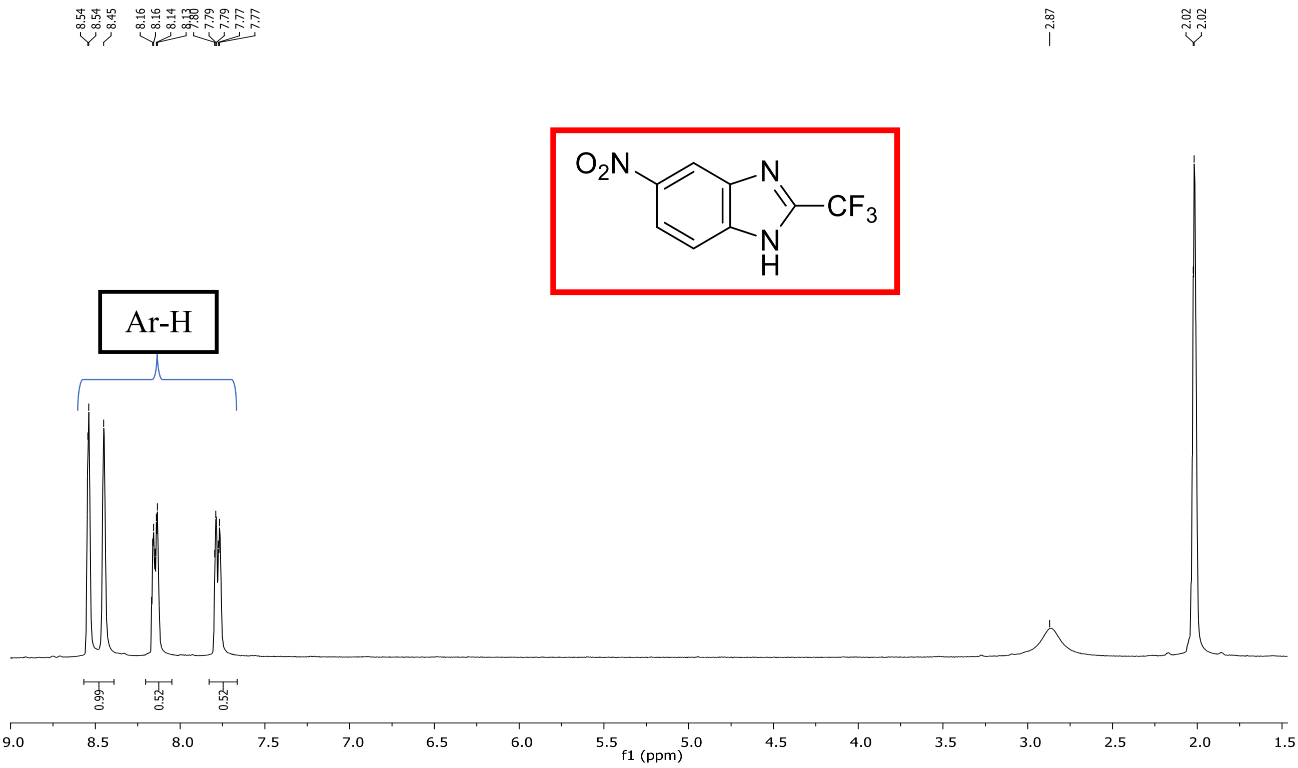

Fig S5: ^{1}H NMR Spectrum of compound **2c**.

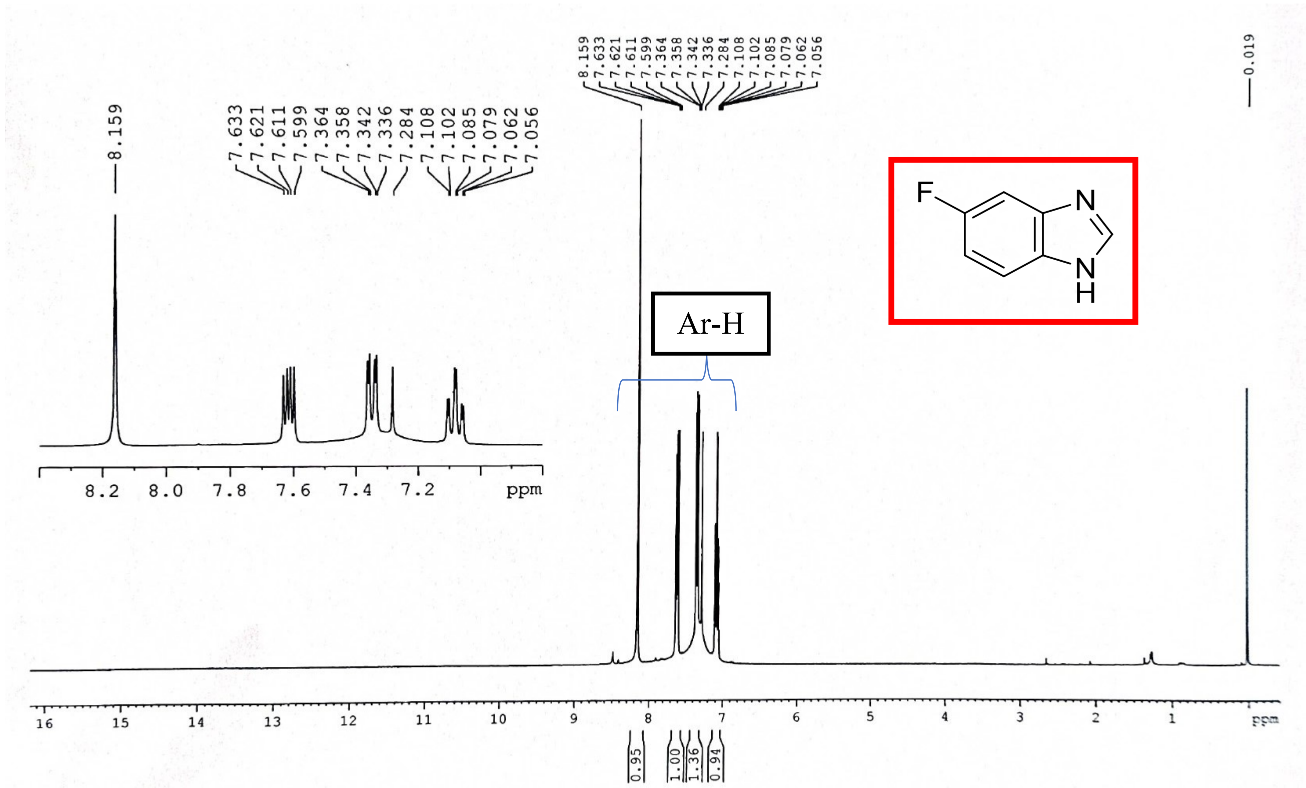

Fig S6: ^{1}H NMR Spectrum of compound **3a**.

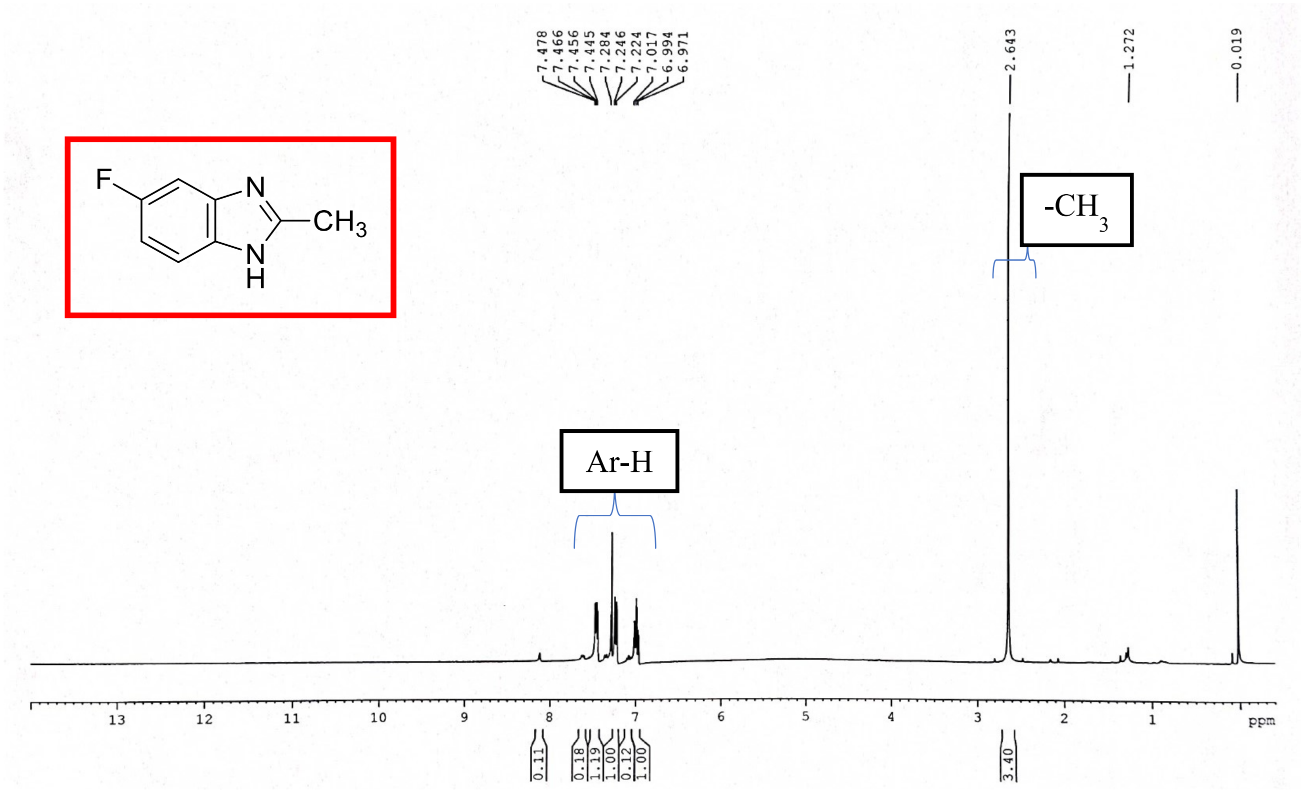

Fig S7: ^{1}H NMR Spectrum of compound **3b**.

Spectral Data of the Synthesized Derivatives (1ai-3bj):

1. IR, NMR and Mass Spectra of compound **1ai:**

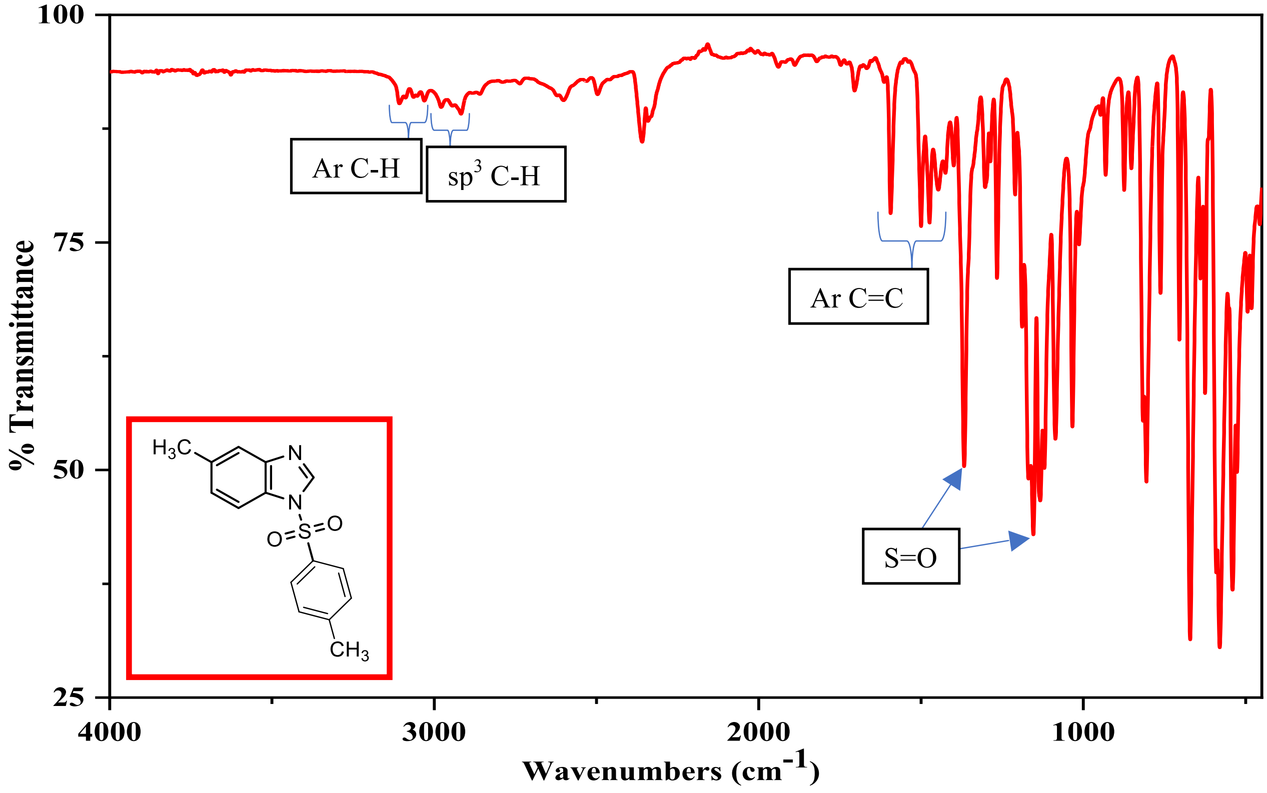

Fig S8: IR Spectrum of compound **1ai**.

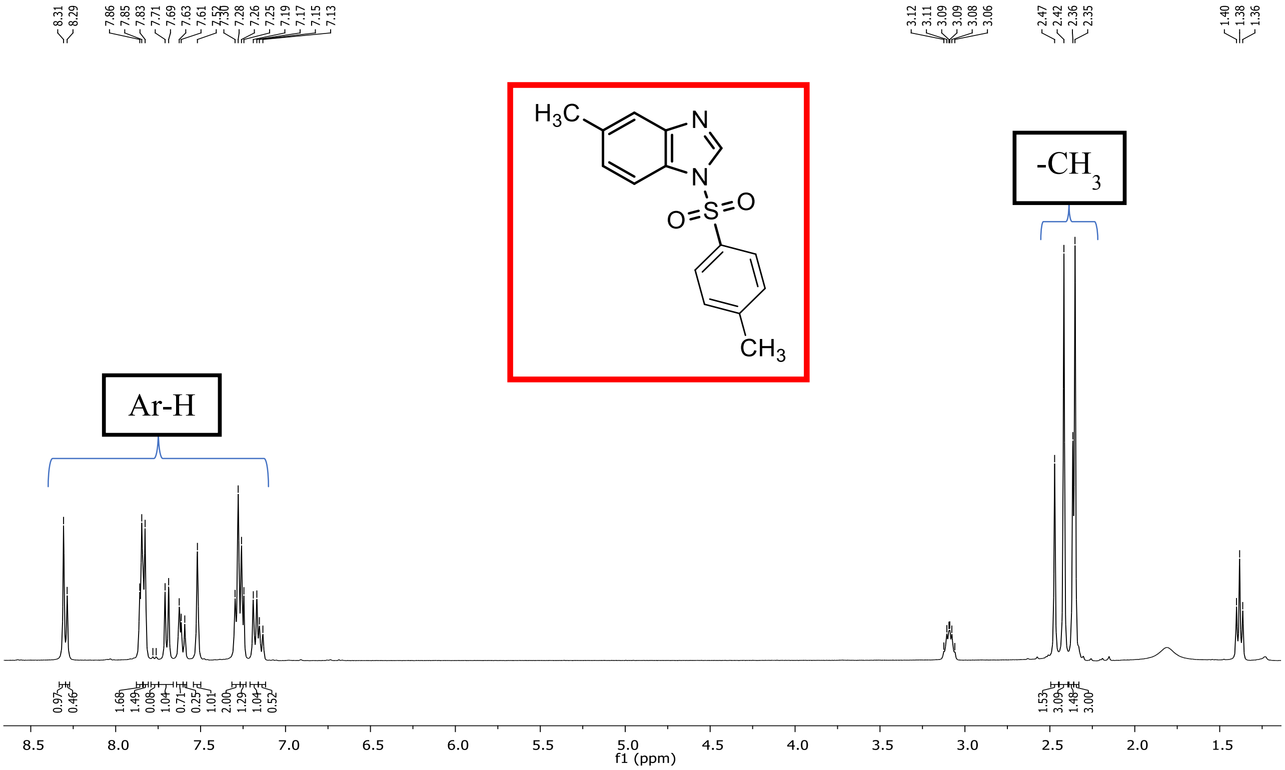

Fig S9: ^{1}H NMR Spectrum of compound **1ai**.

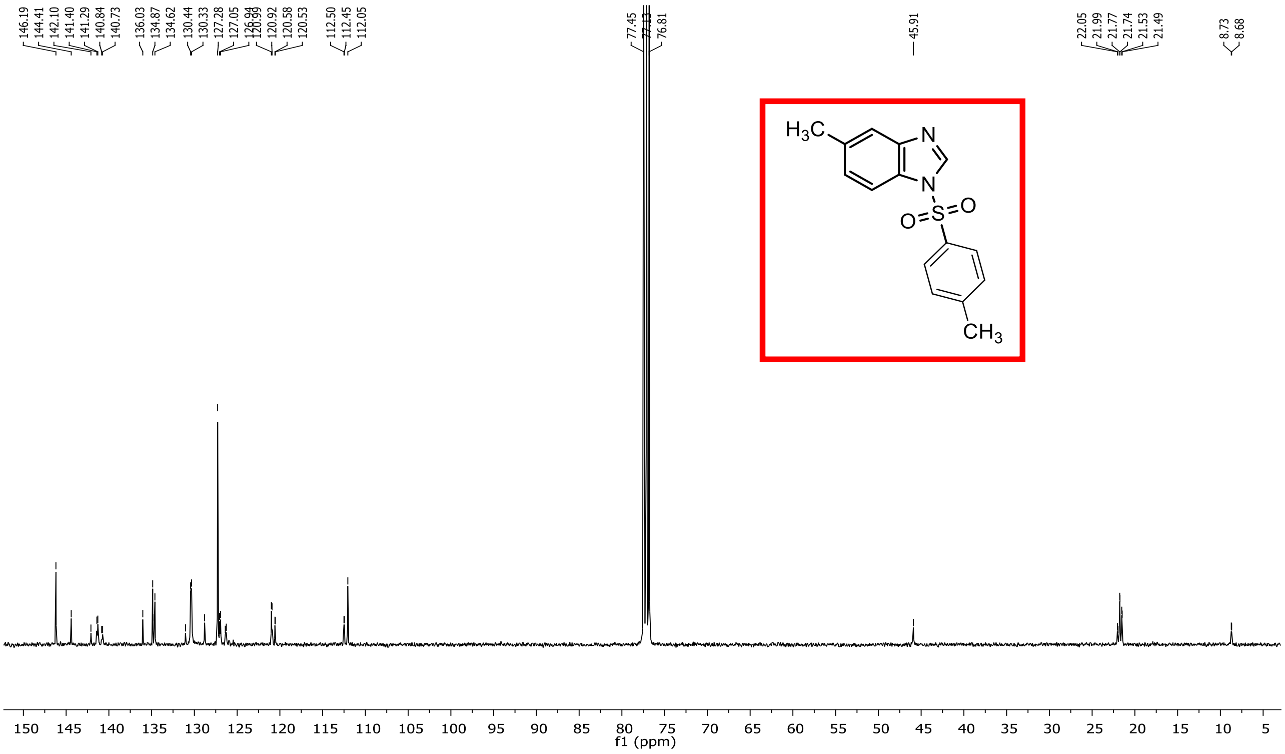

Fig S10: ^{13}C NMR Spectrum of compound **1ai**.

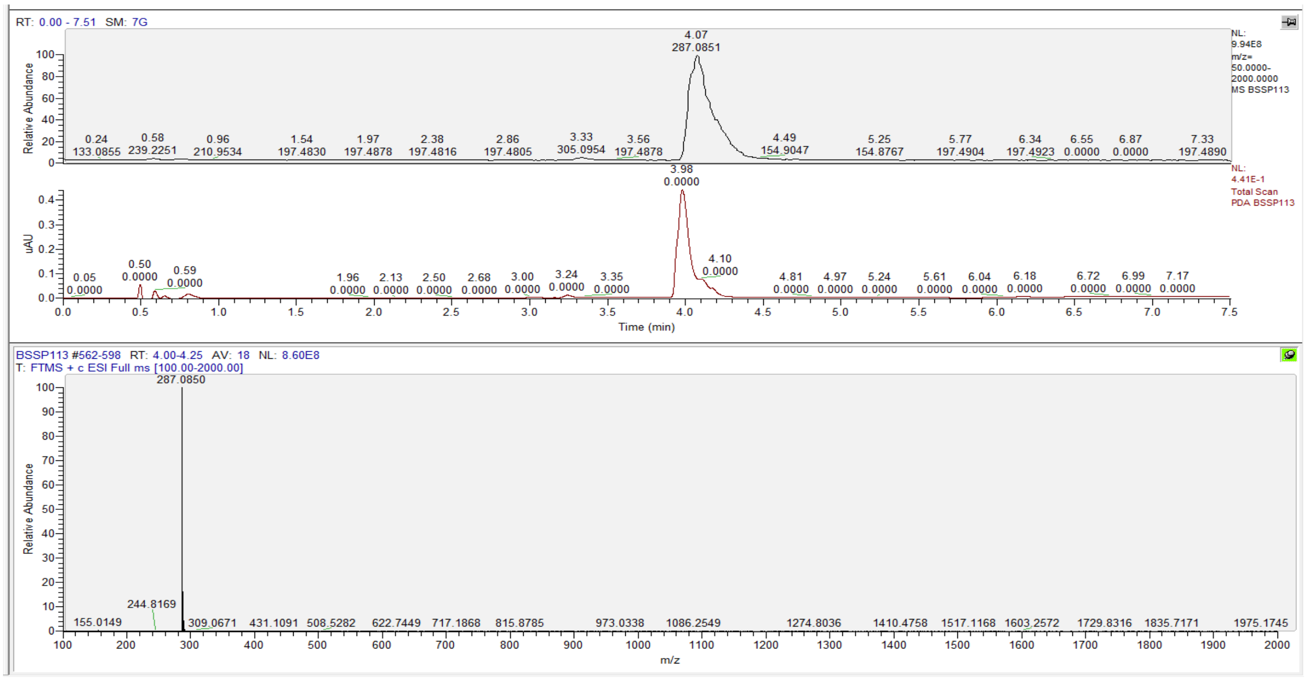

Fig S11: Mass Spectrum of compound **1ai**.

2. IR and NMR Spectra of compound **1aj:**

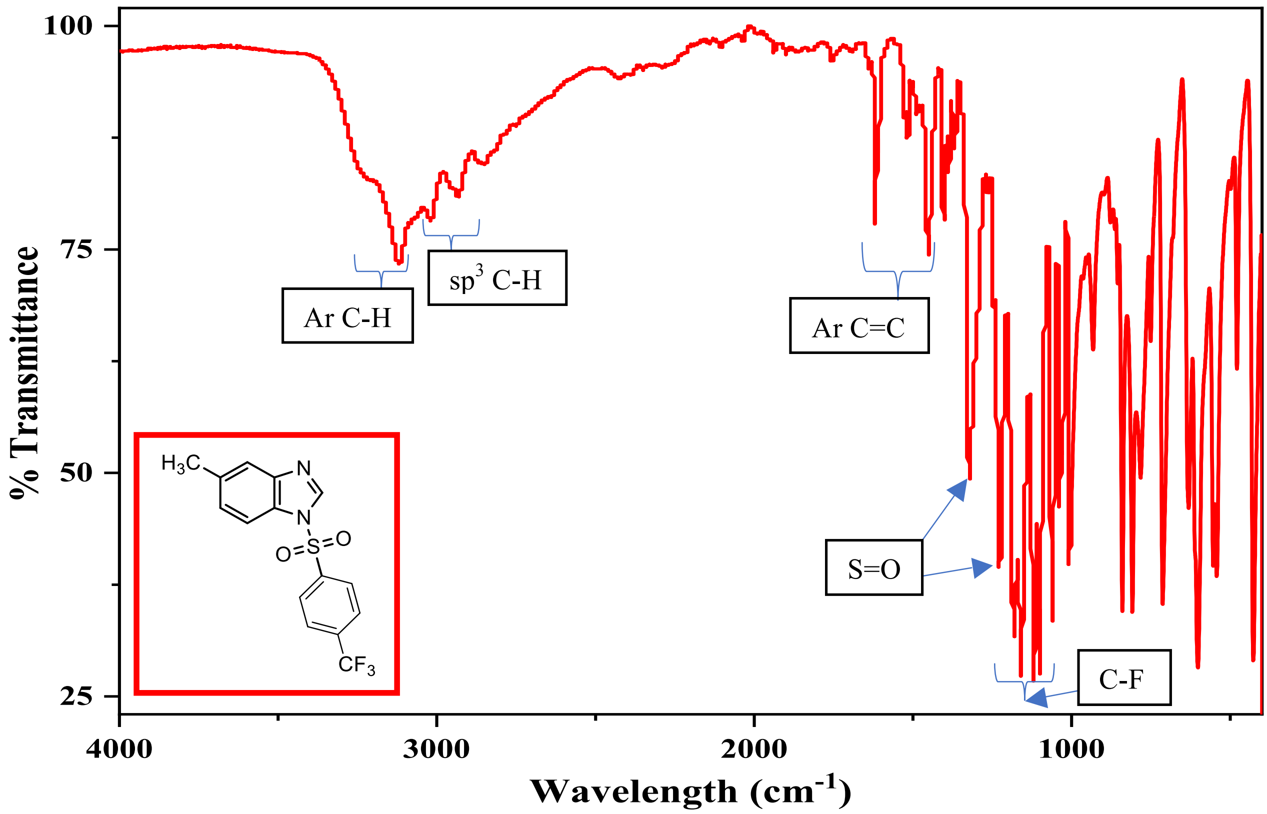

Fig S12: IR Spectrum of compound **1aj**.

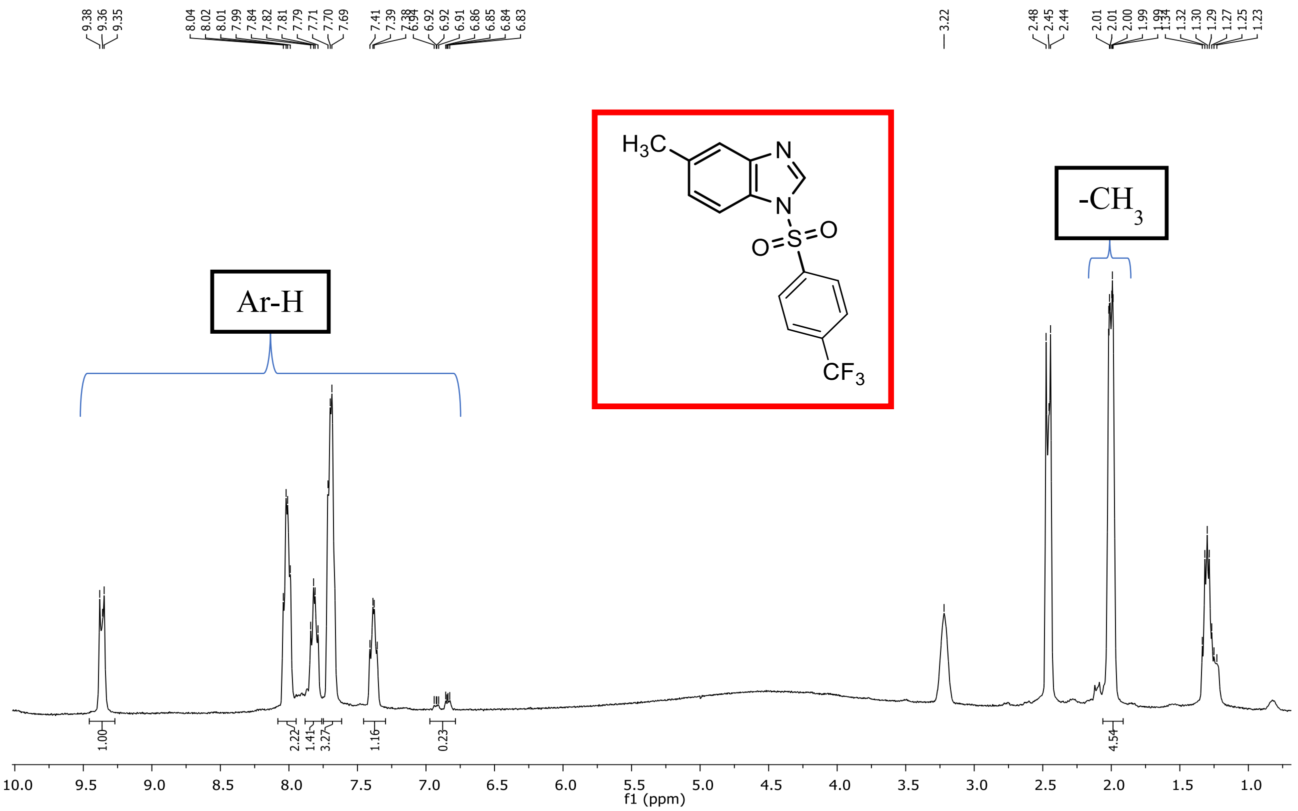

Fig S13: ^{1}H NMR Spectrum of compound **1aj**.

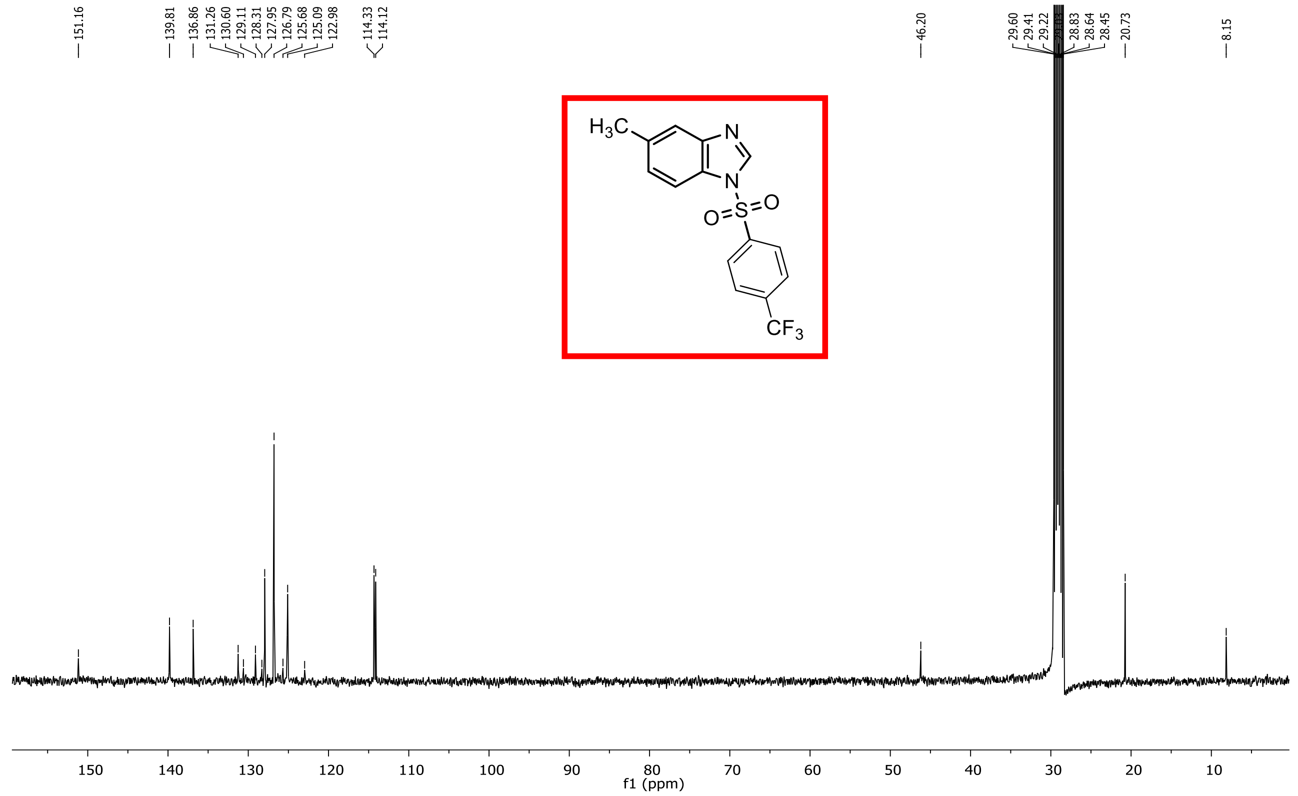

Fig S14: ^{13}C NMR Spectrum of compound **1aj**.

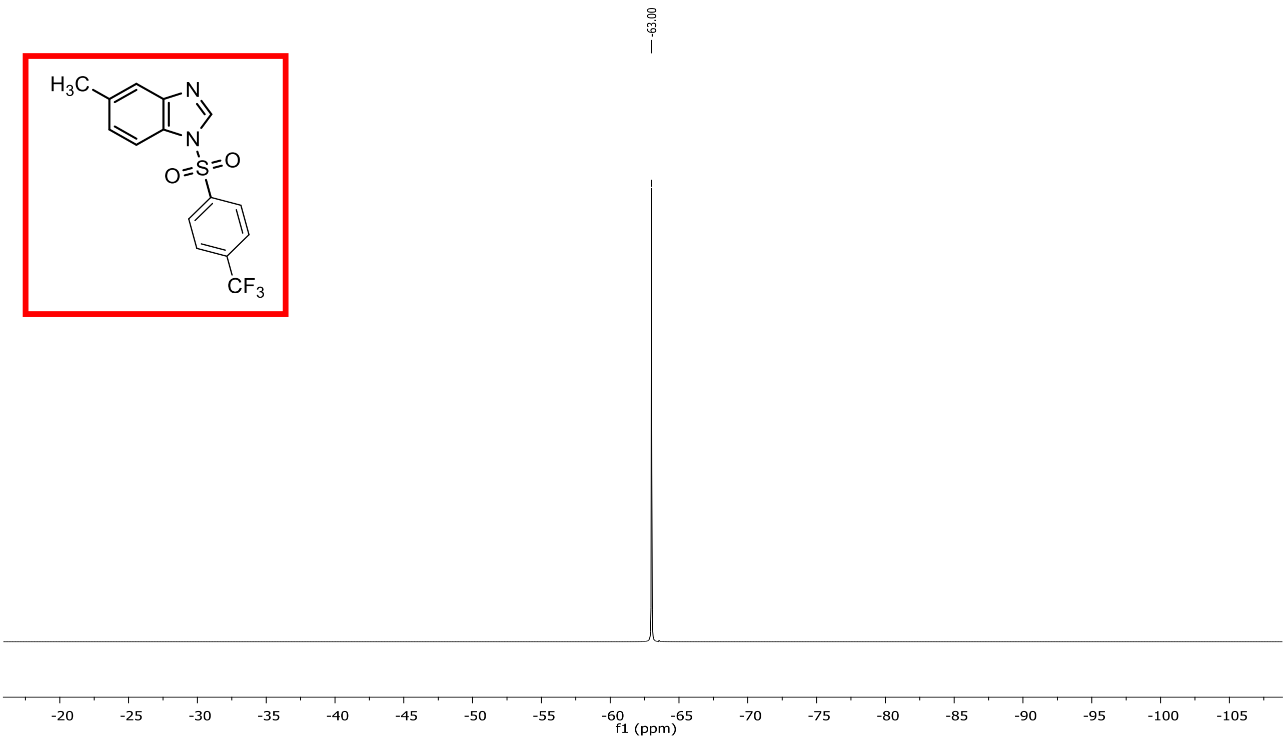

Fig S15: ^{19}F NMR Spectrum of compound **1aj**.

3. IR, NMR and Mass Spectra of compound **1bi:**

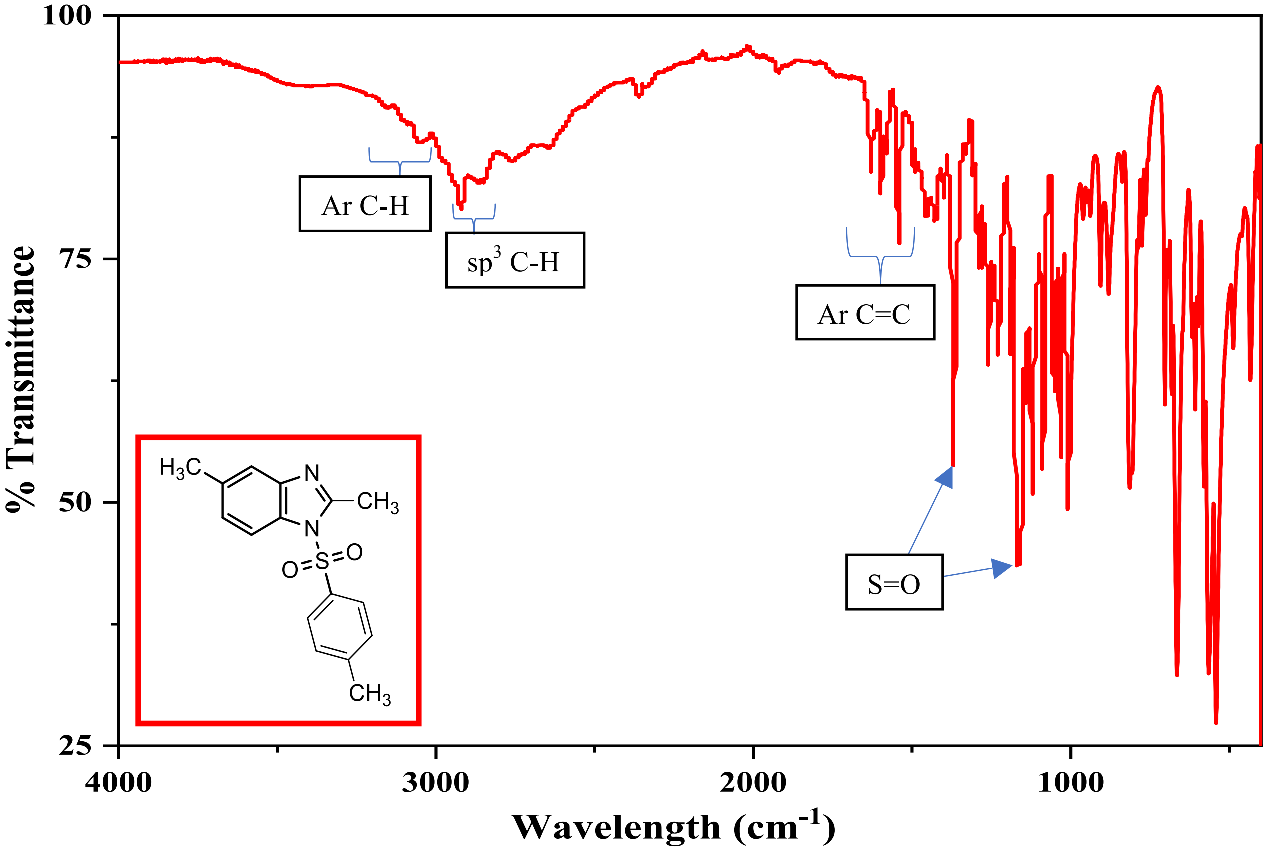

Fig S16: IR Spectrum of compound **1bi**.

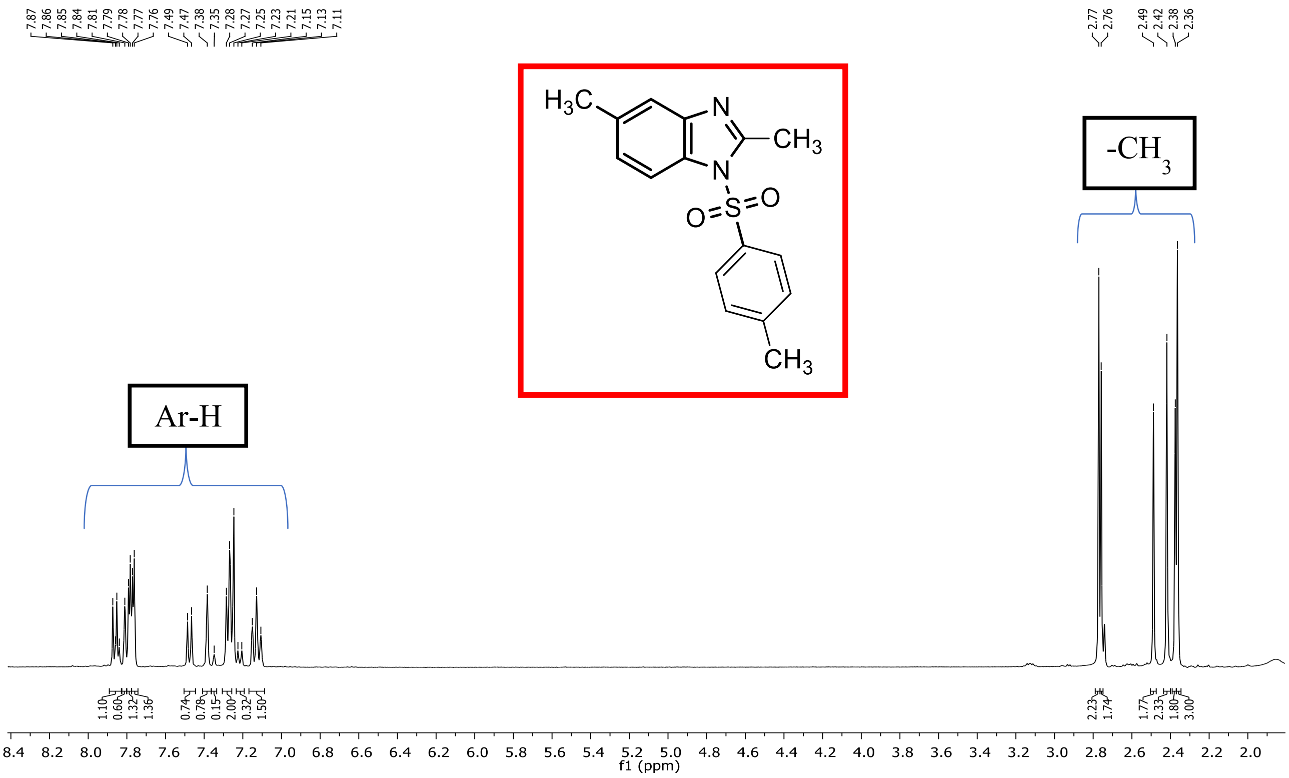

Fig S17: ^{1}H NMR Spectrum of compound **1bi**.

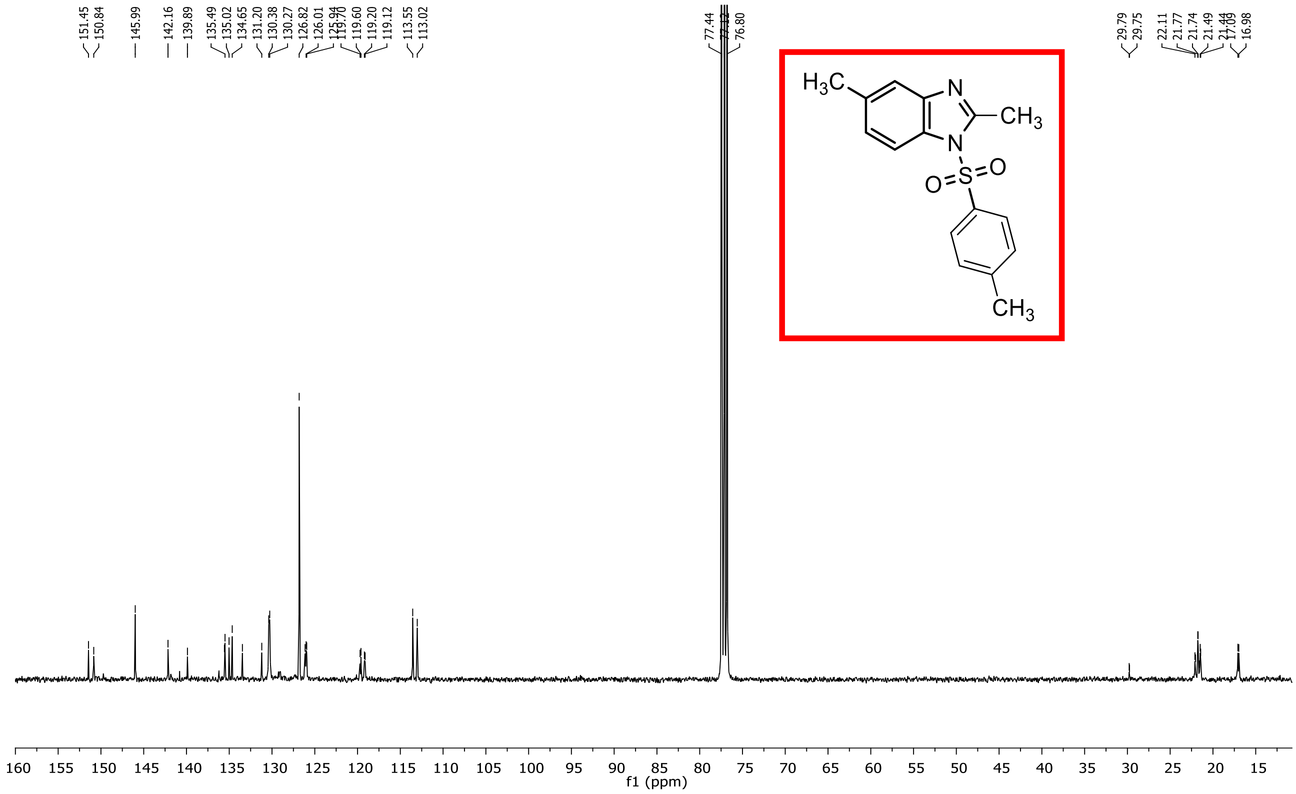

Fig S18: ^{13}C NMR Spectrum of compound **1bi**.

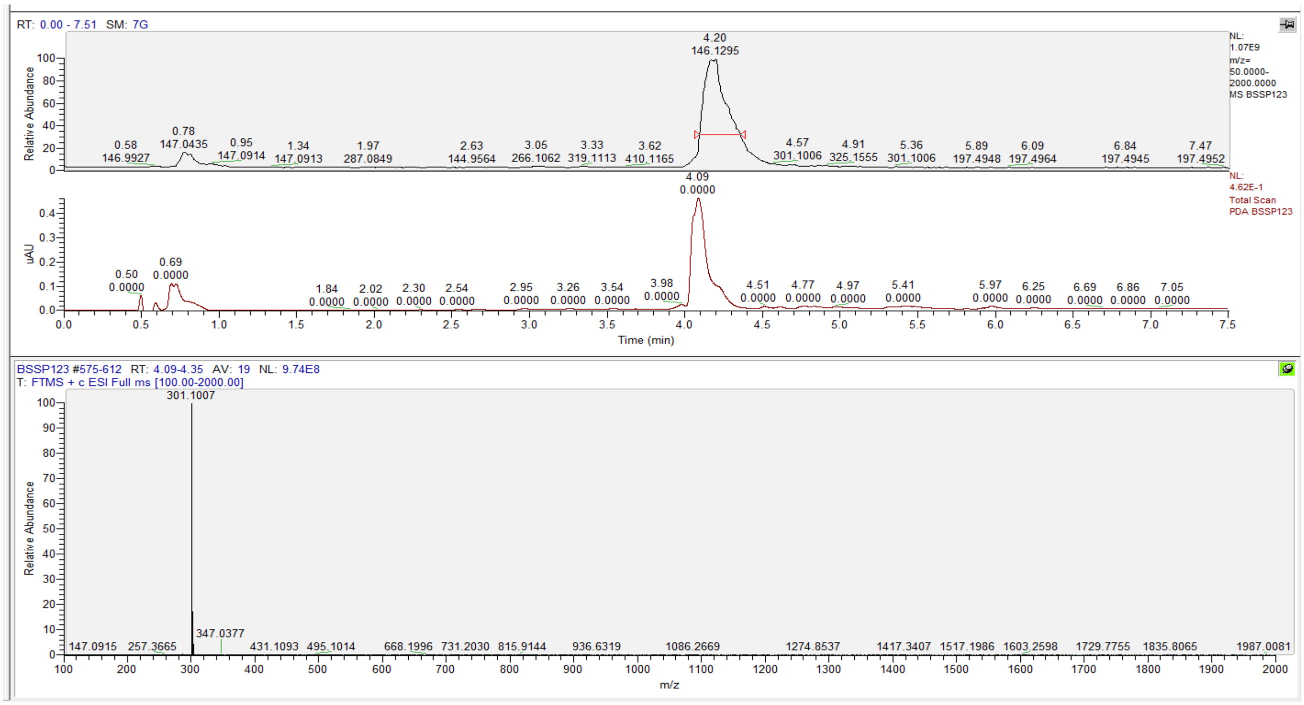

Fig S19: Mass Spectrum of compound **1bi**.

4. IR, NMR and Mass Spectra of compound **1bj:**

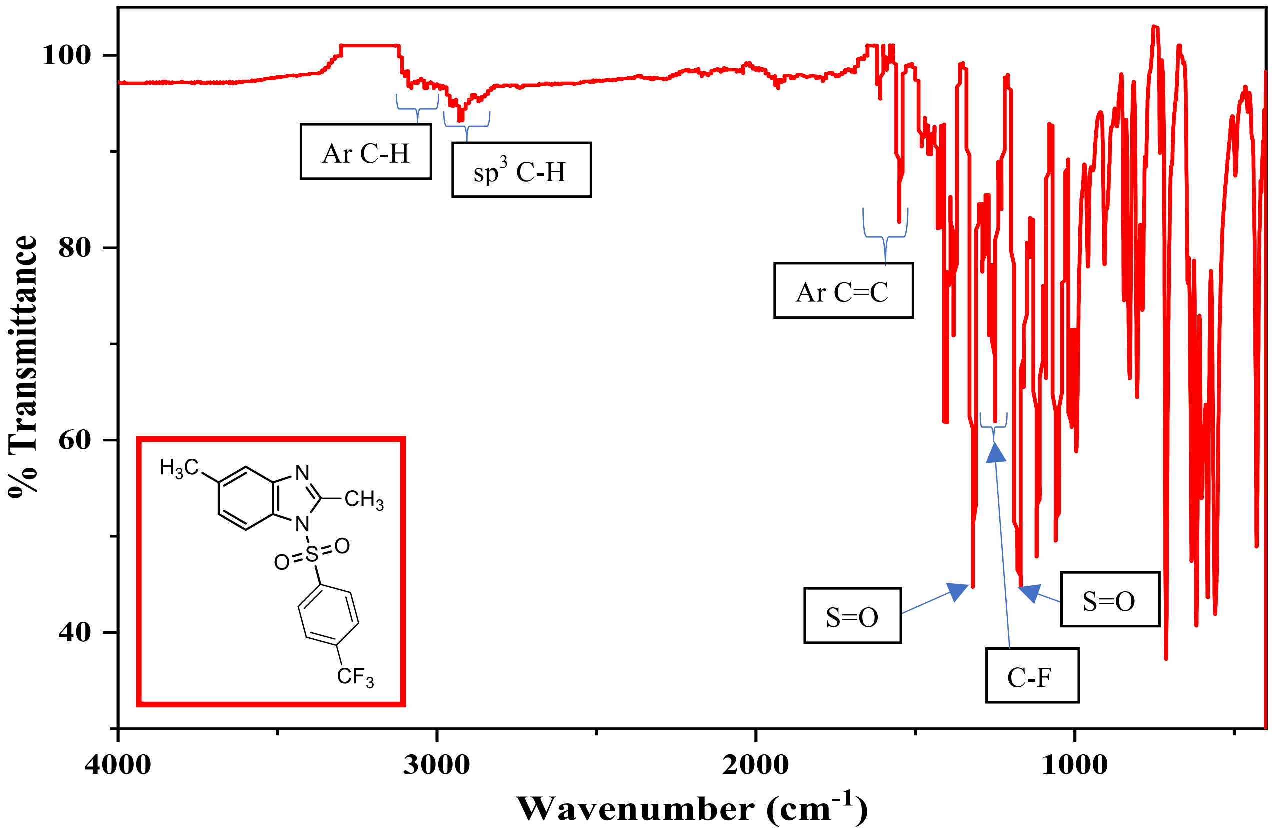

Fig S20: IR Spectrum of compound **1bj**.

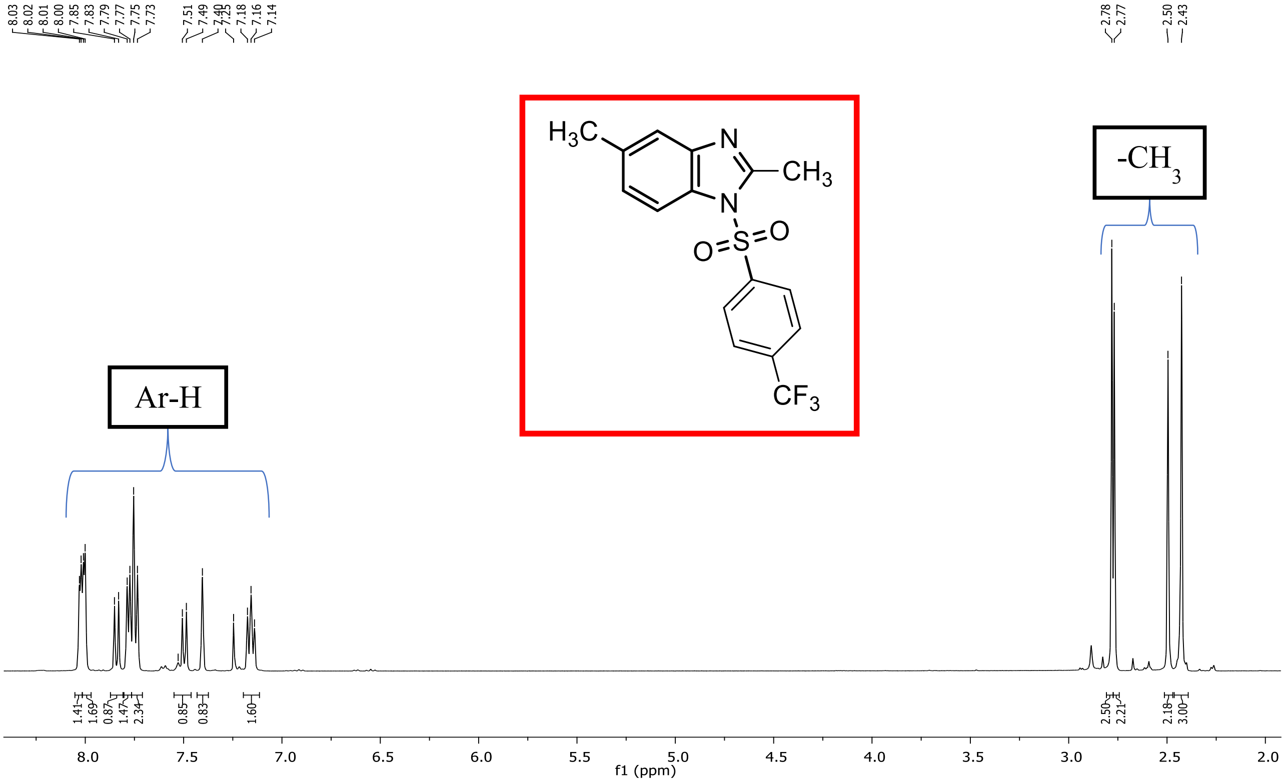

Fig S21: ^{1}H NMR Spectrum of compound **1bj**.

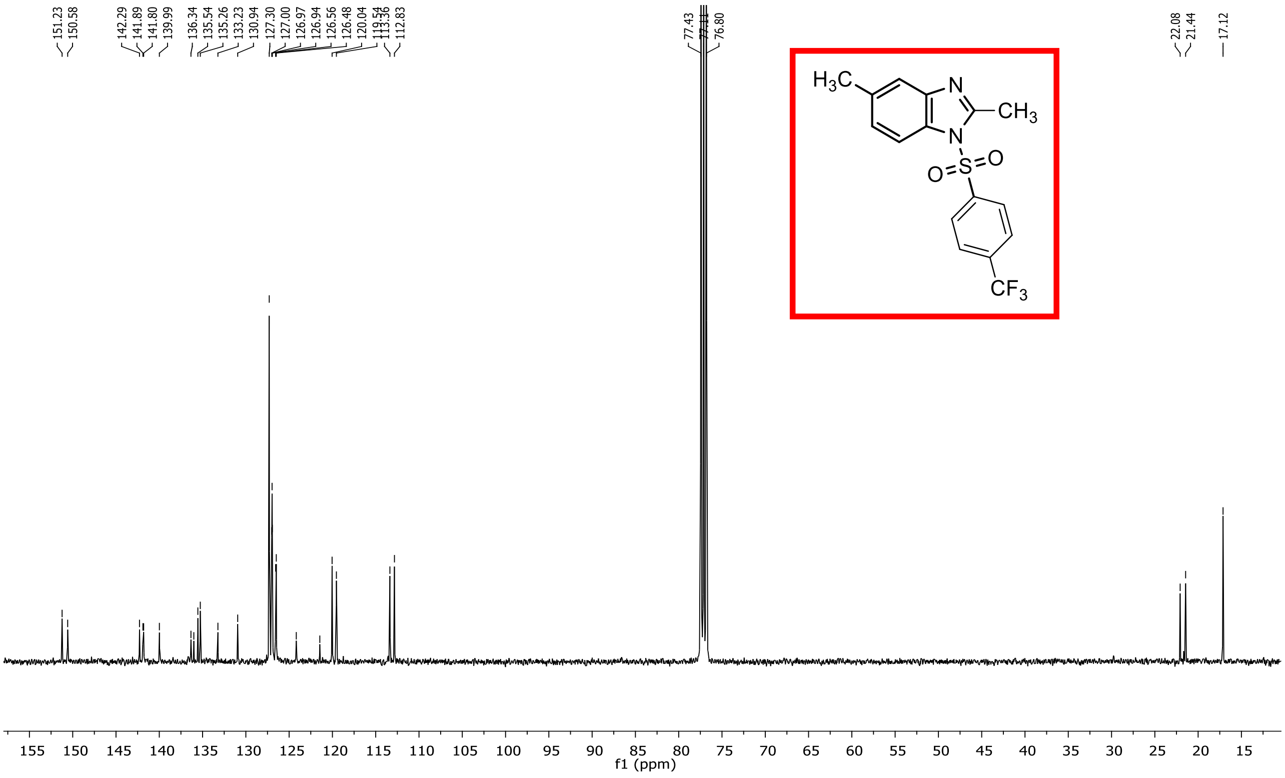

Fig S22: ^{13}C NMR Spectrum of compound **1bj**.

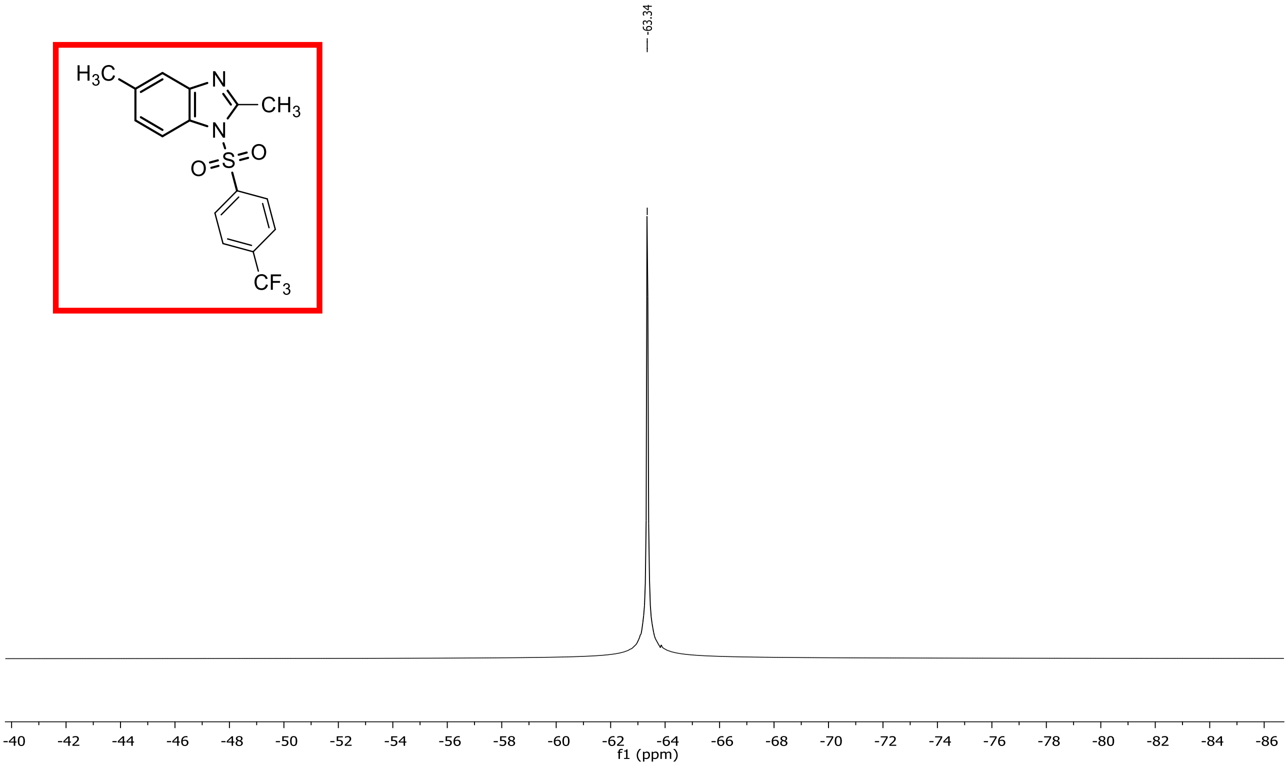

Fig S23: ^{19}F NMR Spectrum of compound **1bj**.

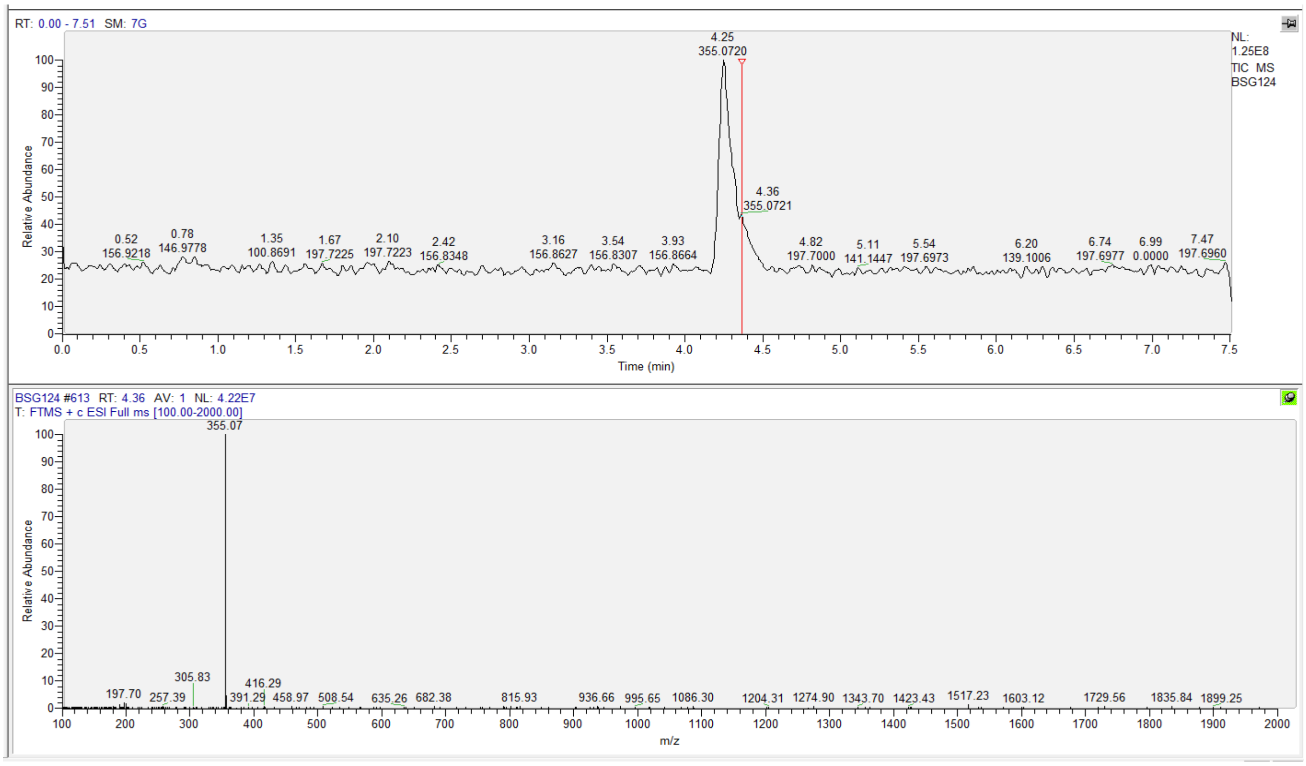

Fig S24: Mass Spectrum of compound **1bj**.

5. IR, NMR and Mass Spectra of compound **2ai:**

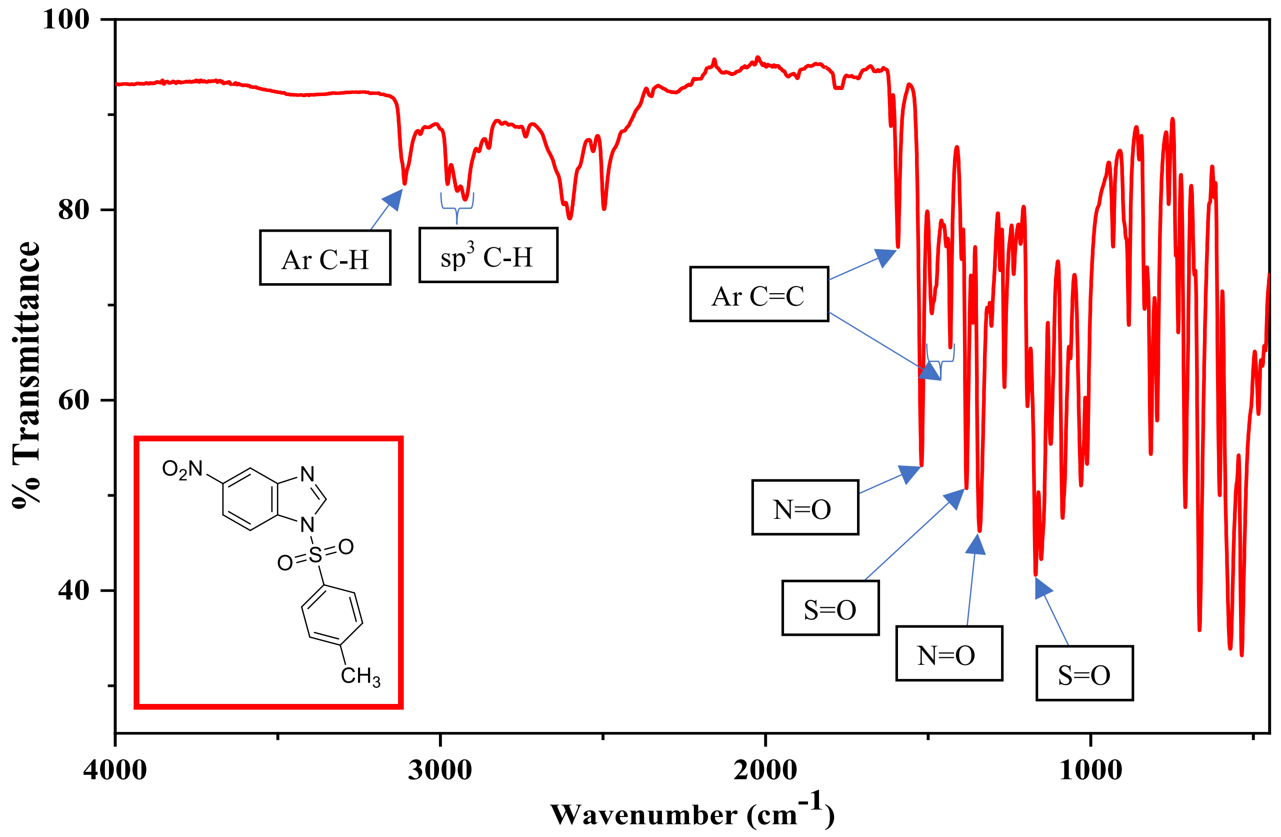

Fig S25: IR Spectrum of compound **2ai**.

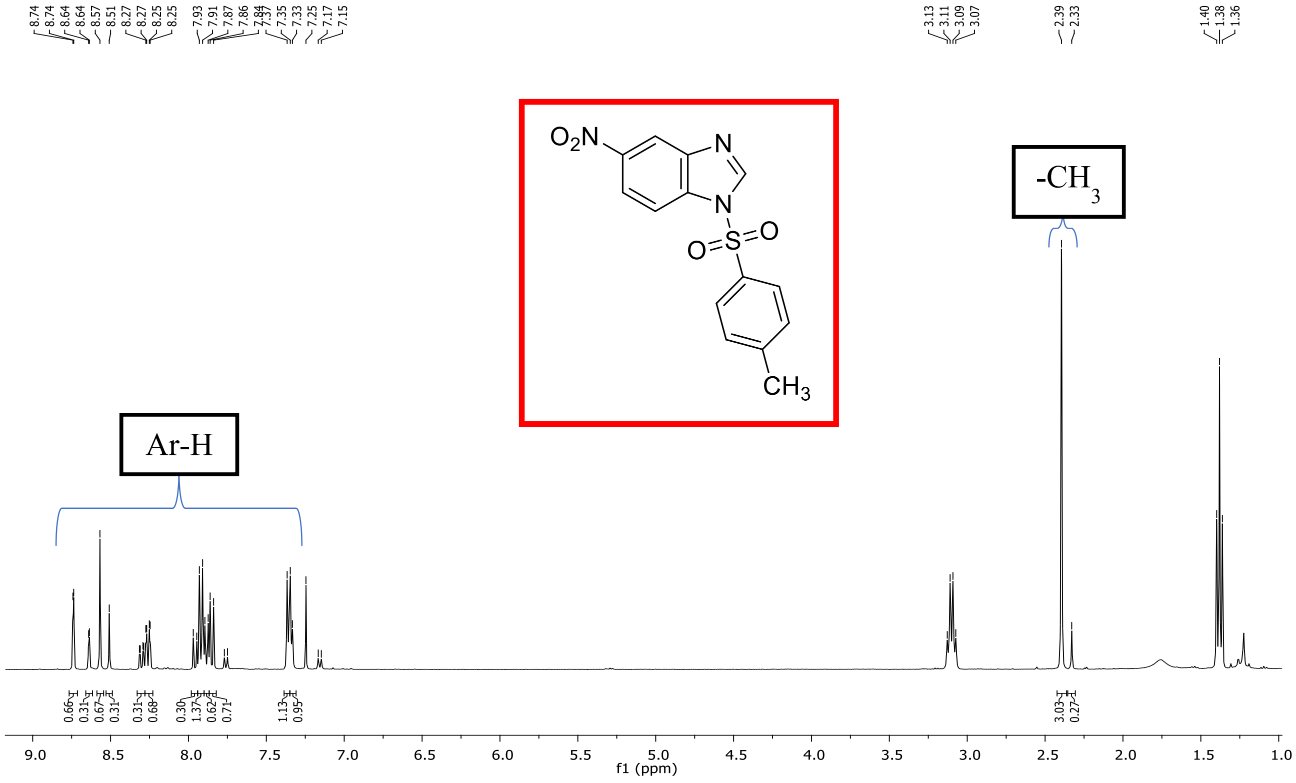

Fig S26: ^{1}H NMR Spectrum of compound **2ai**.

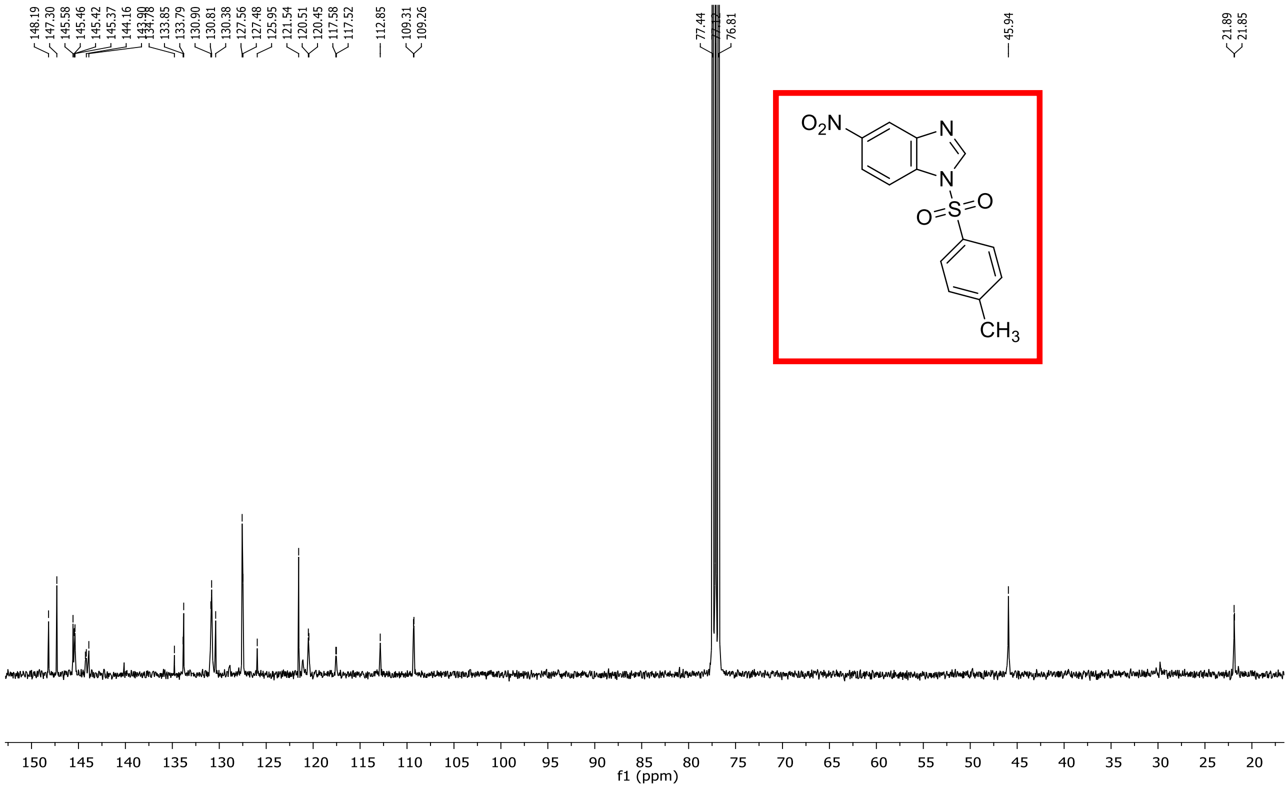

Fig S27: ^{13}C NMR Spectrum of compound **2ai**.

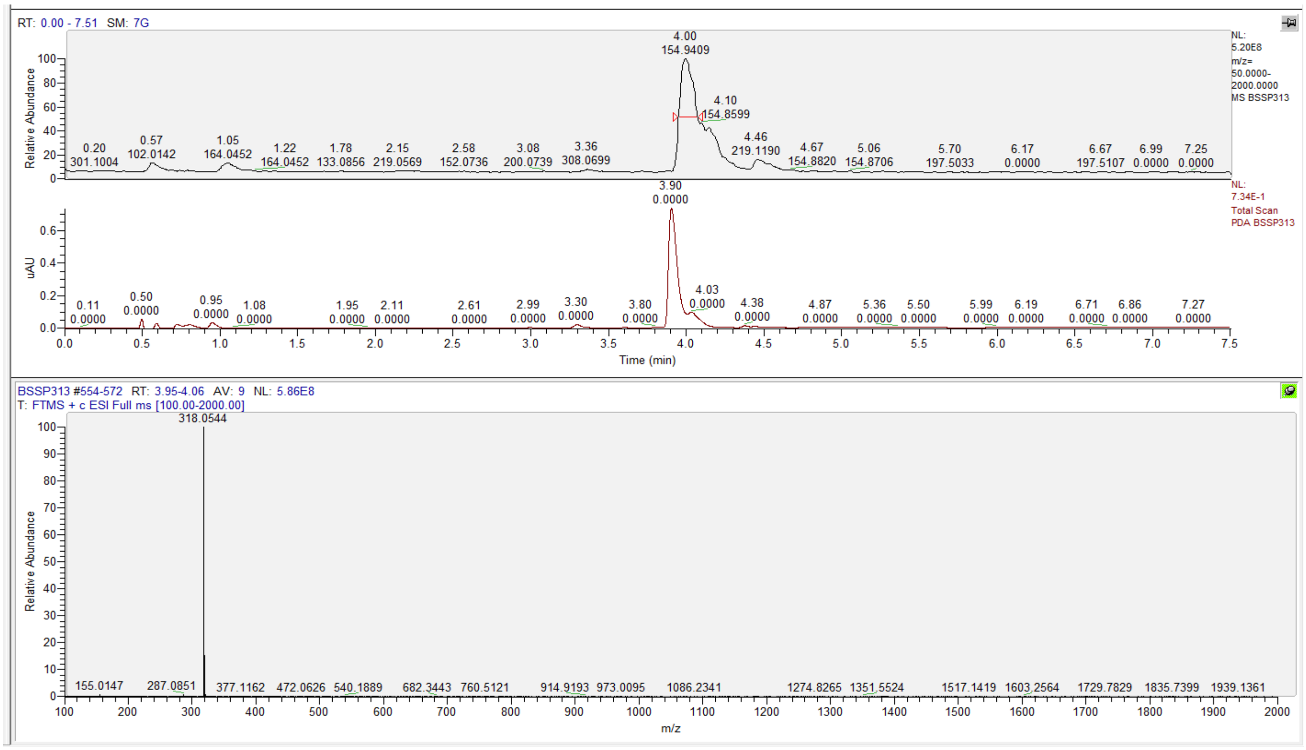

Fig S28: Mass Spectrum of compound **2ai**.

6. IR, NMR and Mass Spectra of compound **2aj**:

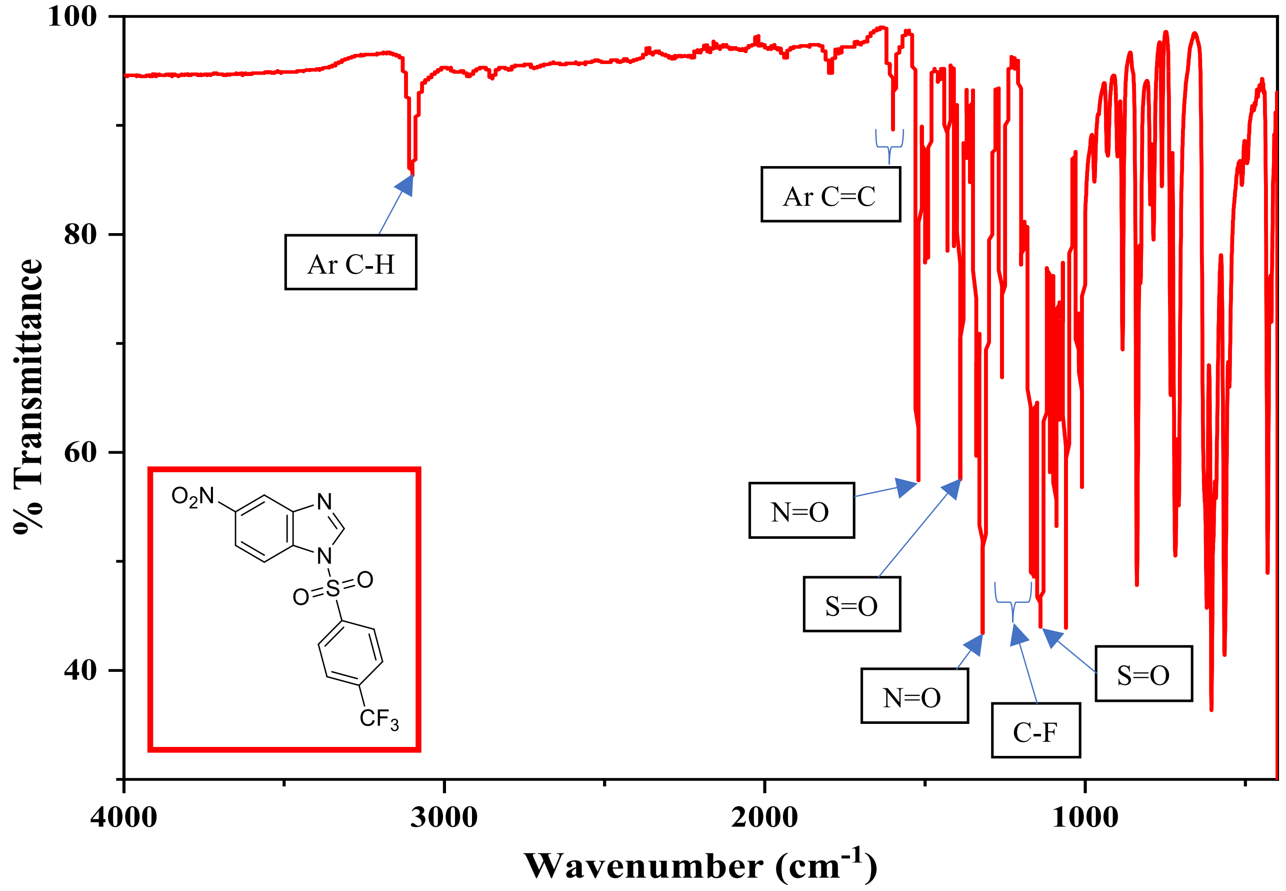

Fig S29: IR Spectrum of compound **2aj**.

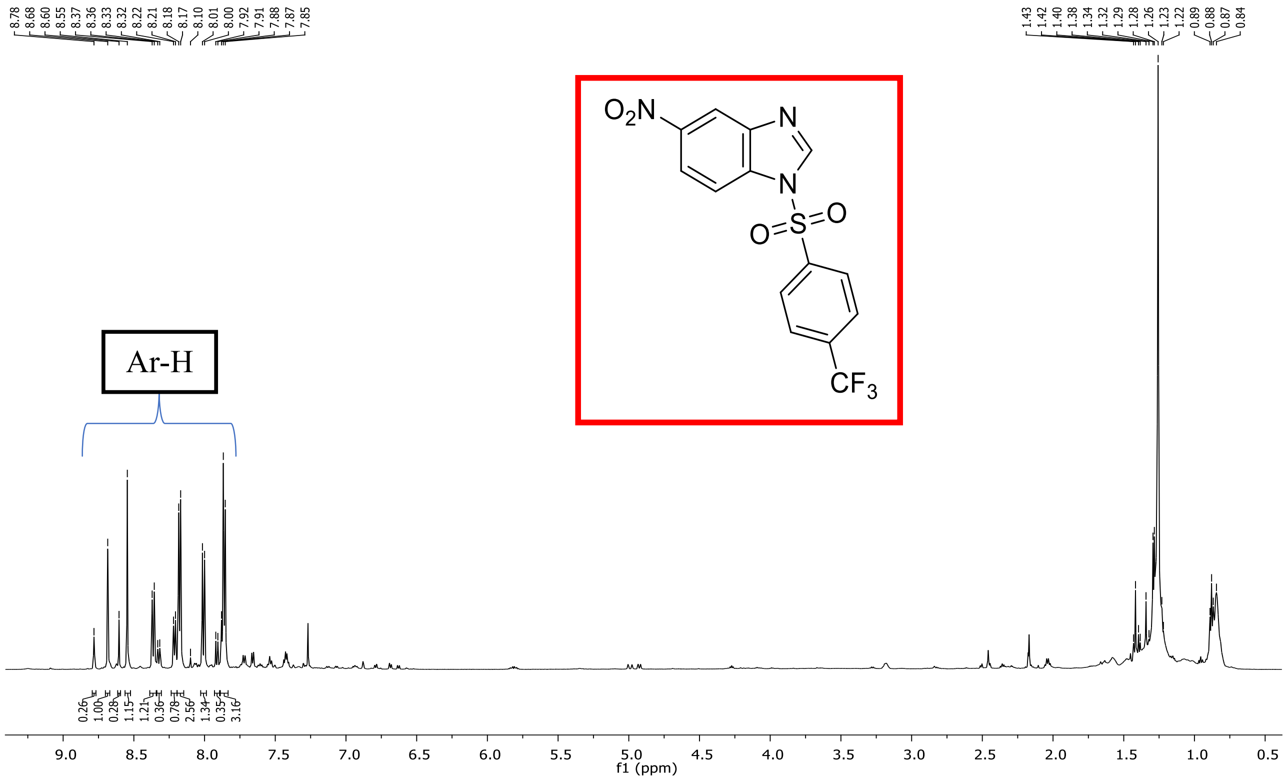

Fig S30: ^{1}H NMR Spectrum of compound **2aj**.

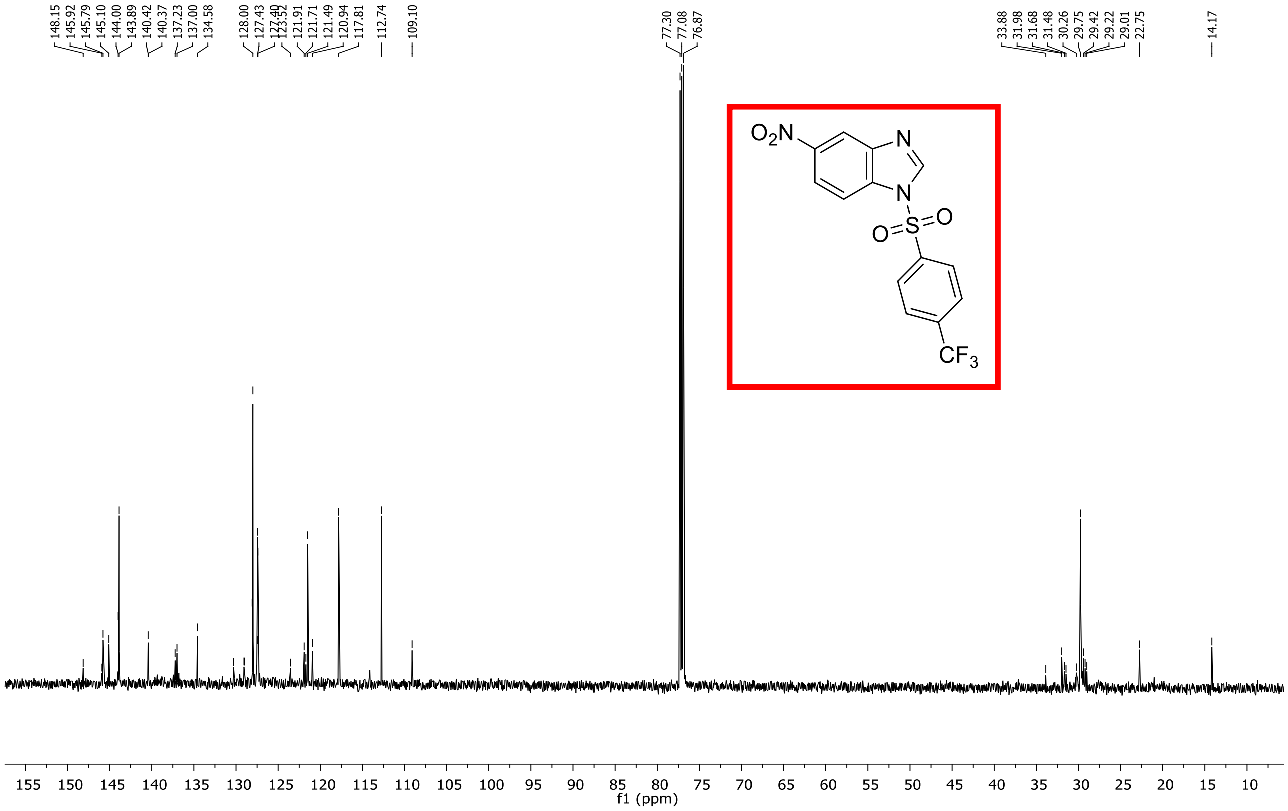

Fig S31: ^{13}C NMR Spectrum of compound **2aj**.

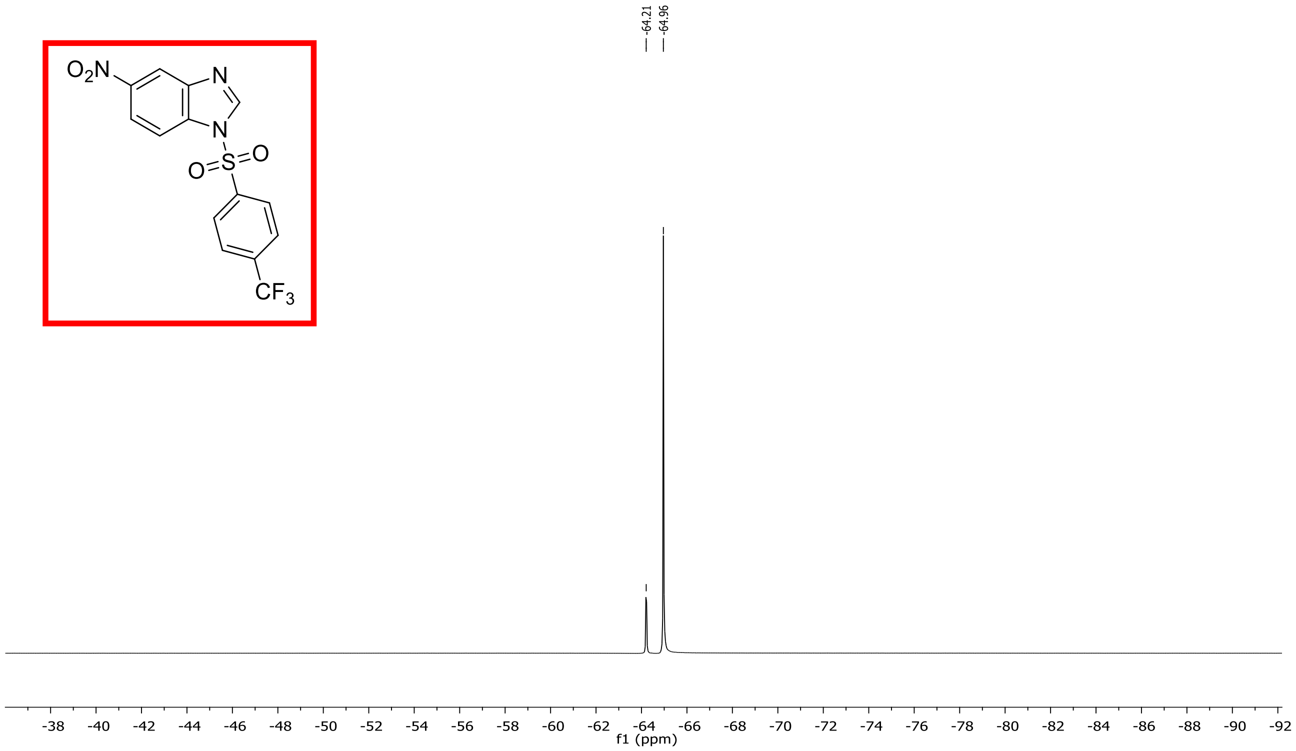

Fig S32: ^{19}F NMR Spectrum of compound **2aj**.

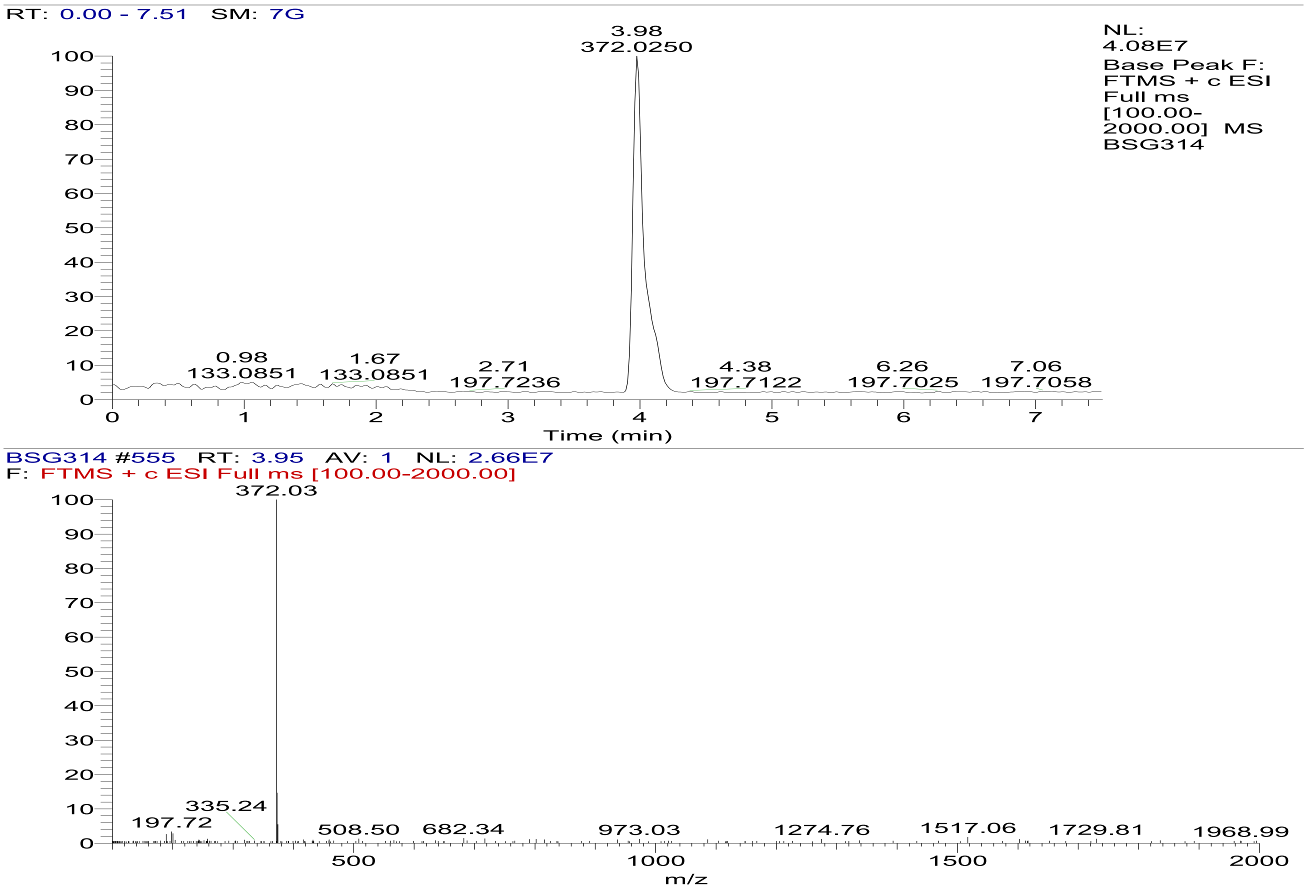

Fig S33: Mass Spectrum of compound **2aj**.

7. IR, NMR and Mass Spectra of compound **2bi:**

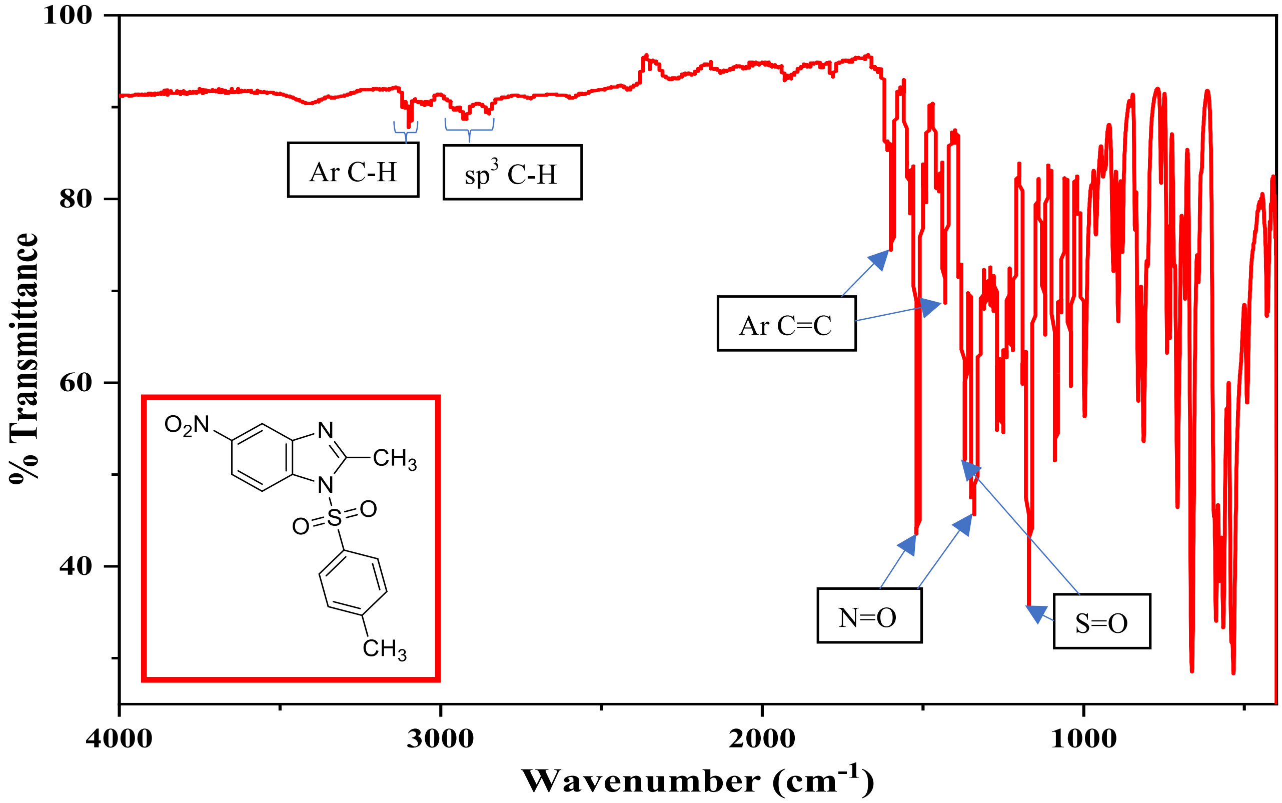

Fig S34: IR Spectrum of compound **2bi**.

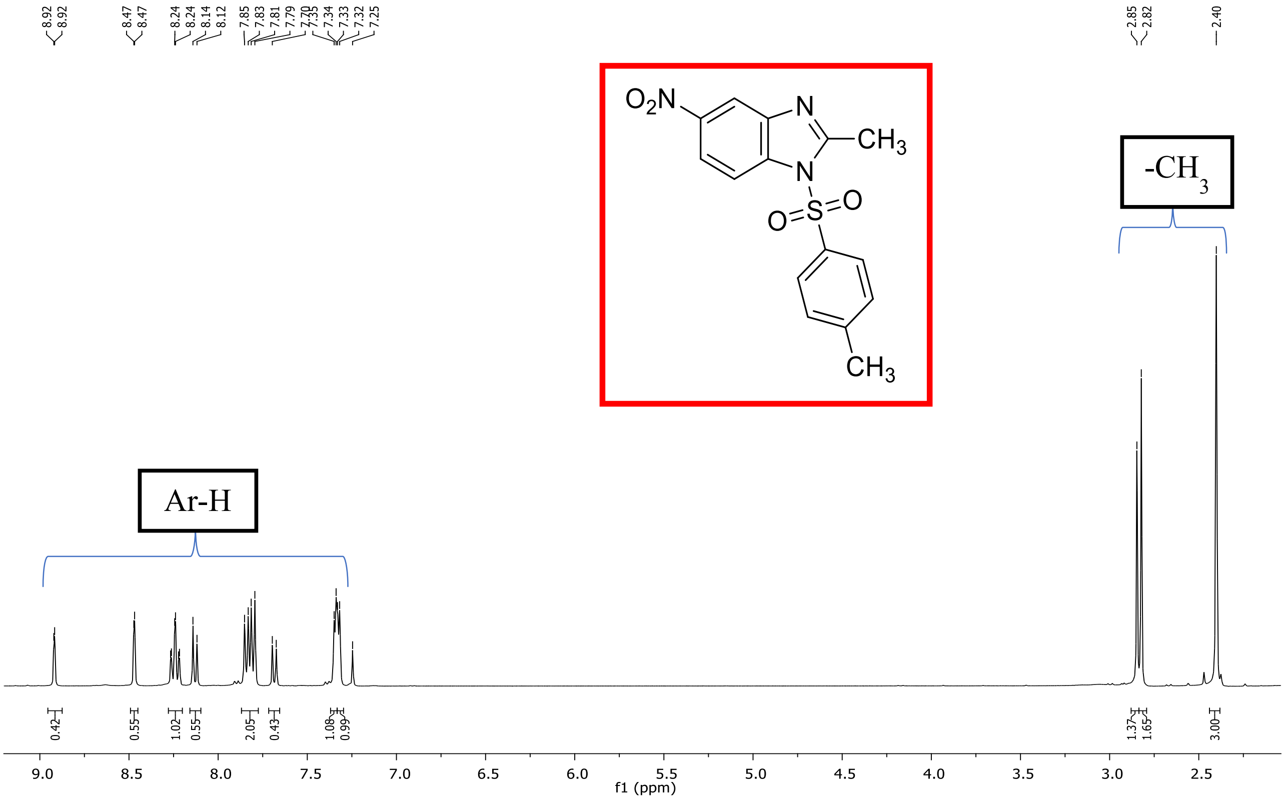

Fig S35: ^{1}H NMR Spectrum of compound **2bi**.

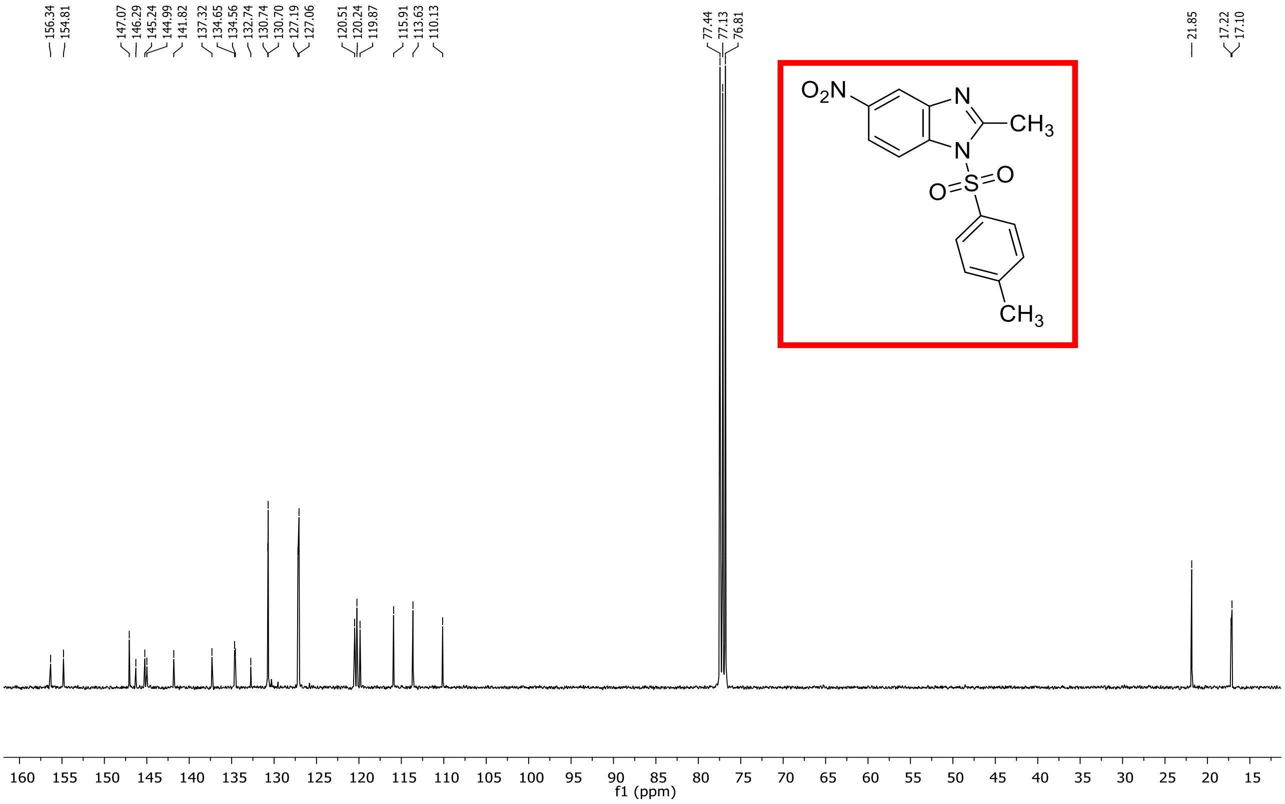

Fig S36: ^{13}C NMR Spectrum of compound **2bi**.

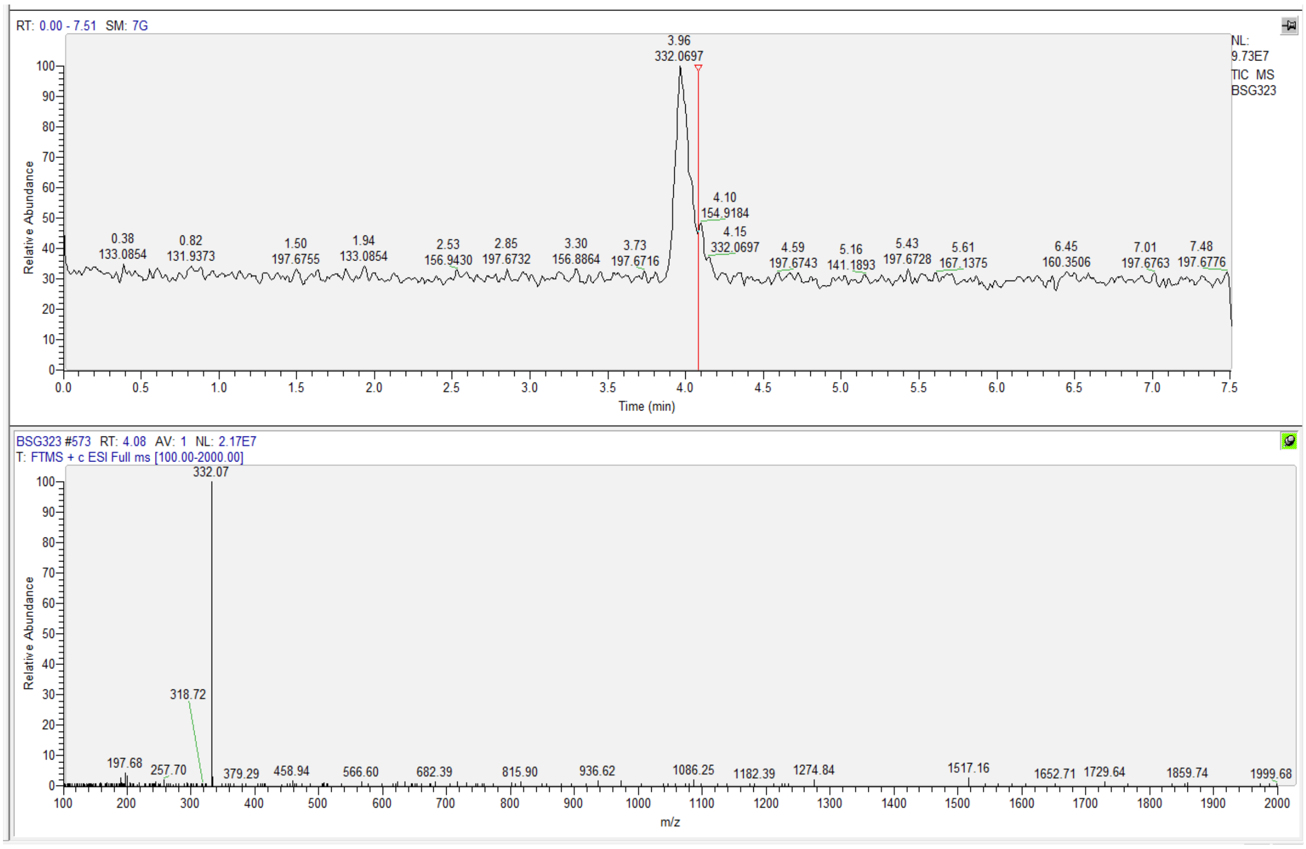

Fig S37: Mass Spectrum of compound **2bi**.

8. IR, NMR and Mass Spectra of compound **2bj**:

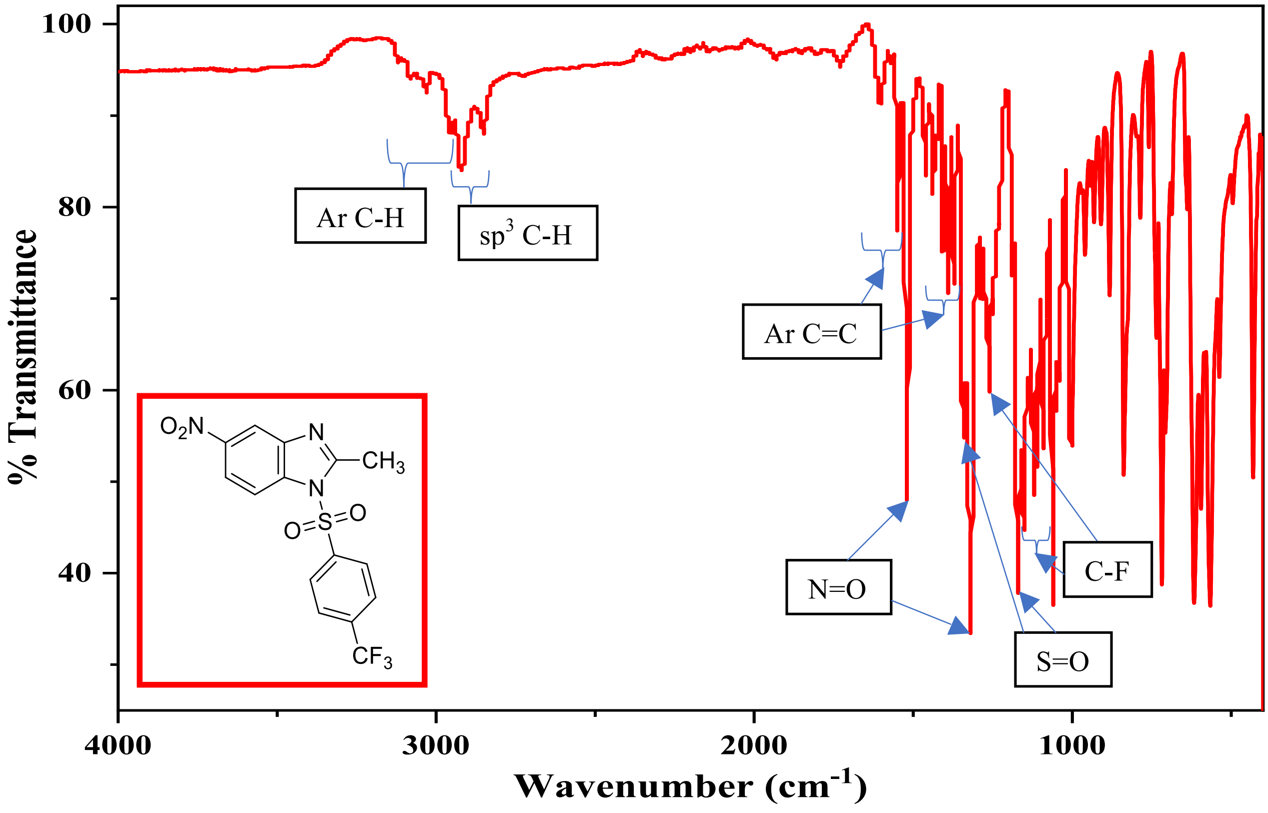

Fig S38: IR Spectrum of compound **2bj**.

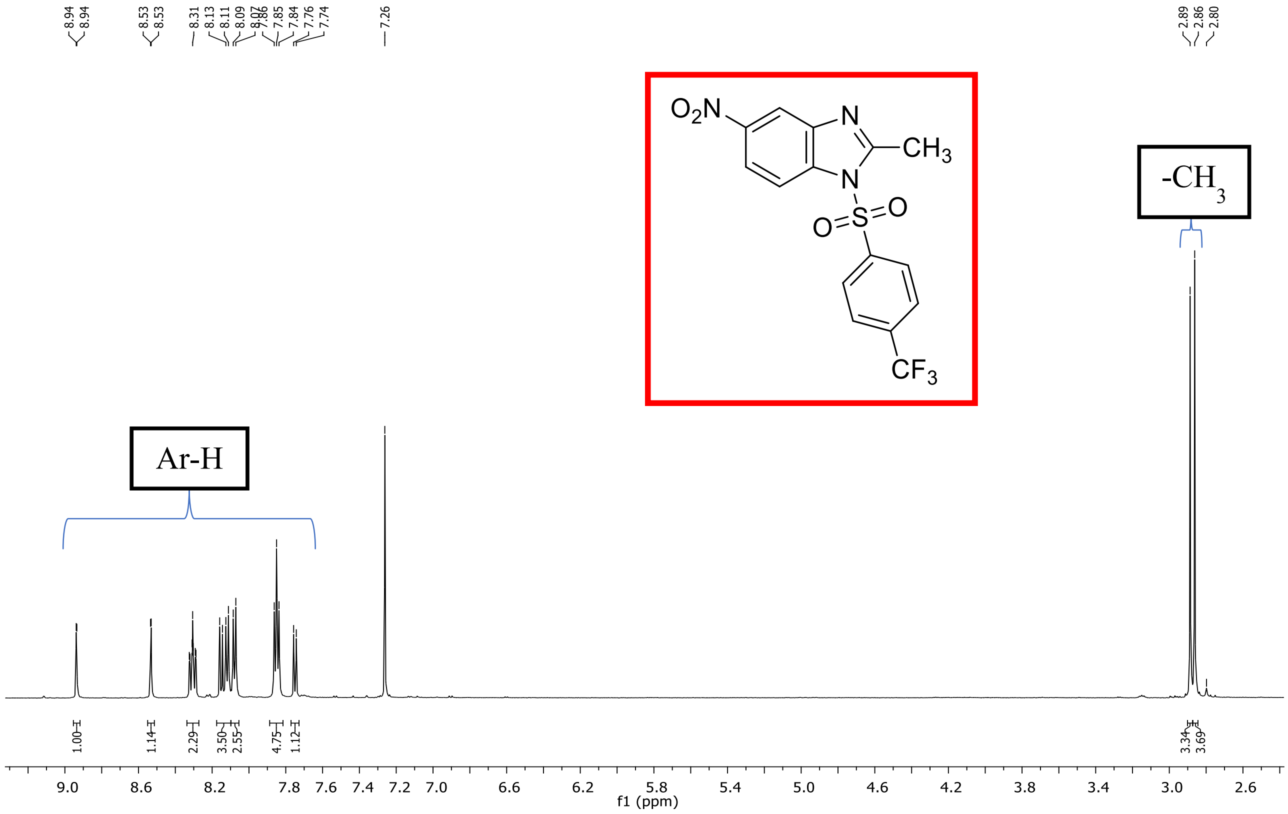

Fig S39: ^{1}H NMR Spectrum of compound **2bj**.

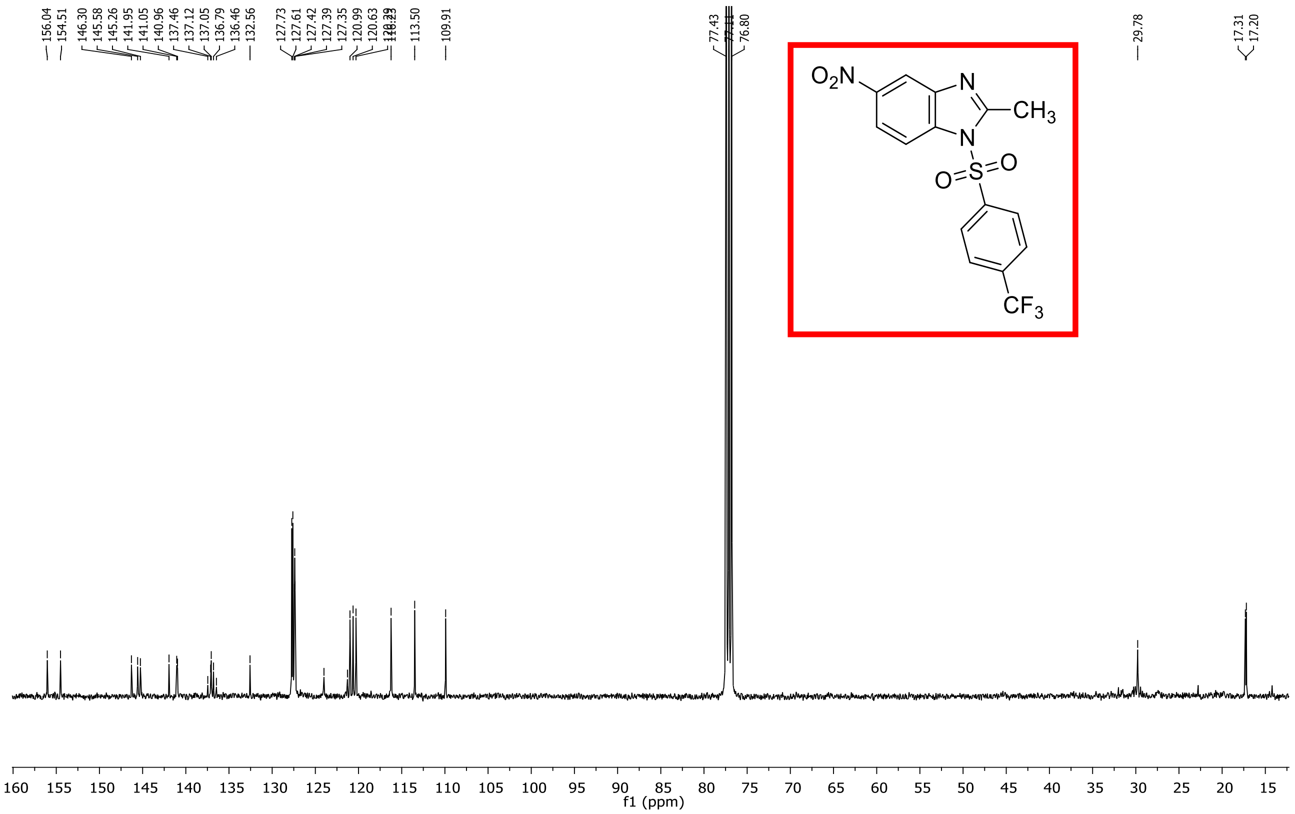

Fig S40: ^{13}C NMR Spectrum of compound **2bj**.

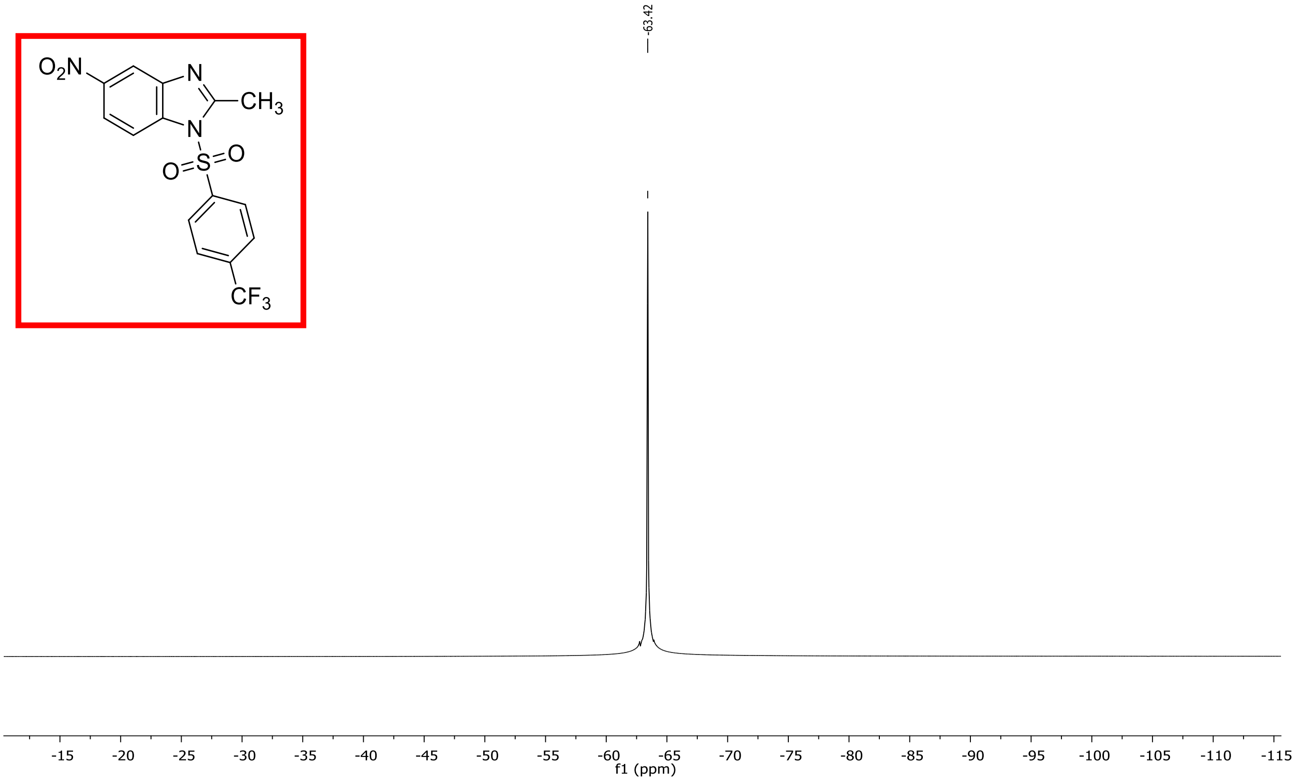

Fig S41: ^{19}F NMR Spectrum of compound **2bj**.

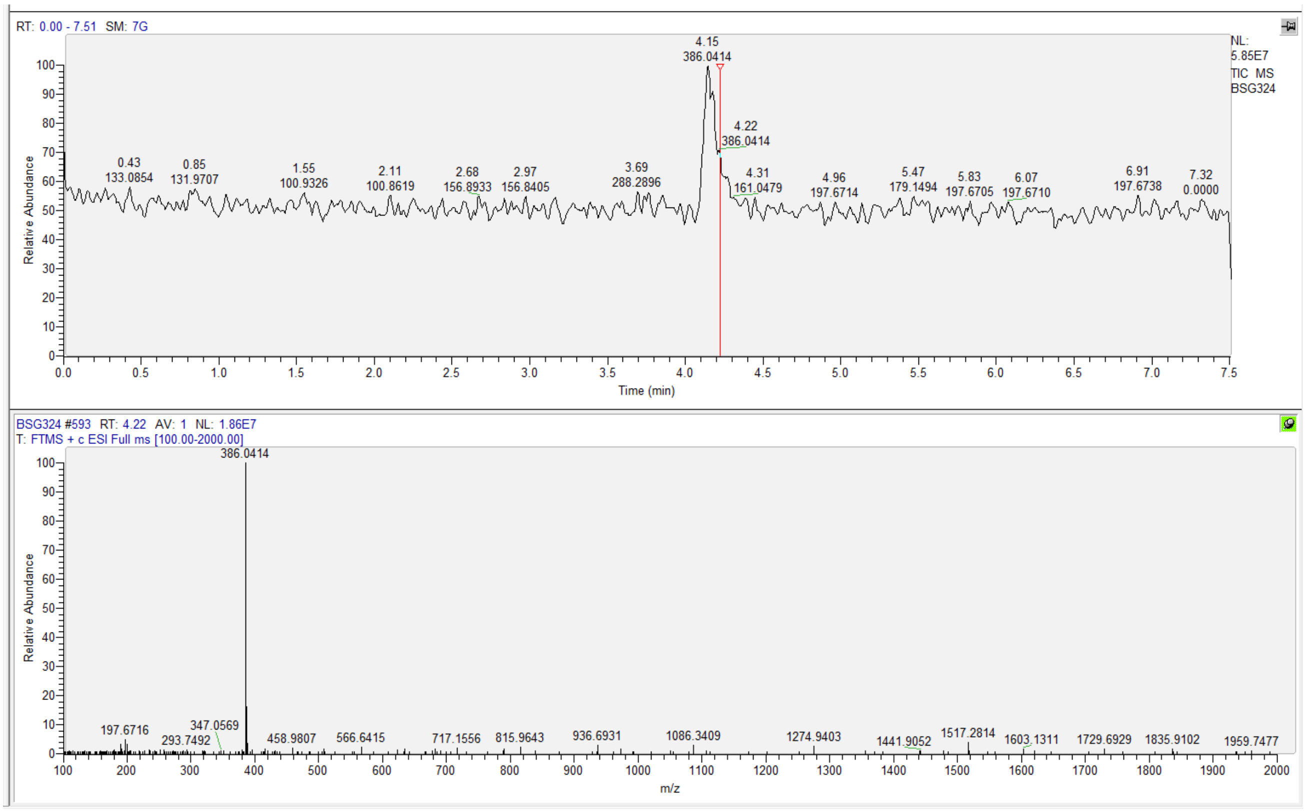

Fig S42: Mass Spectrum of compound **2bj**.

9. IR and NMR Spectra of compound **2ci:**

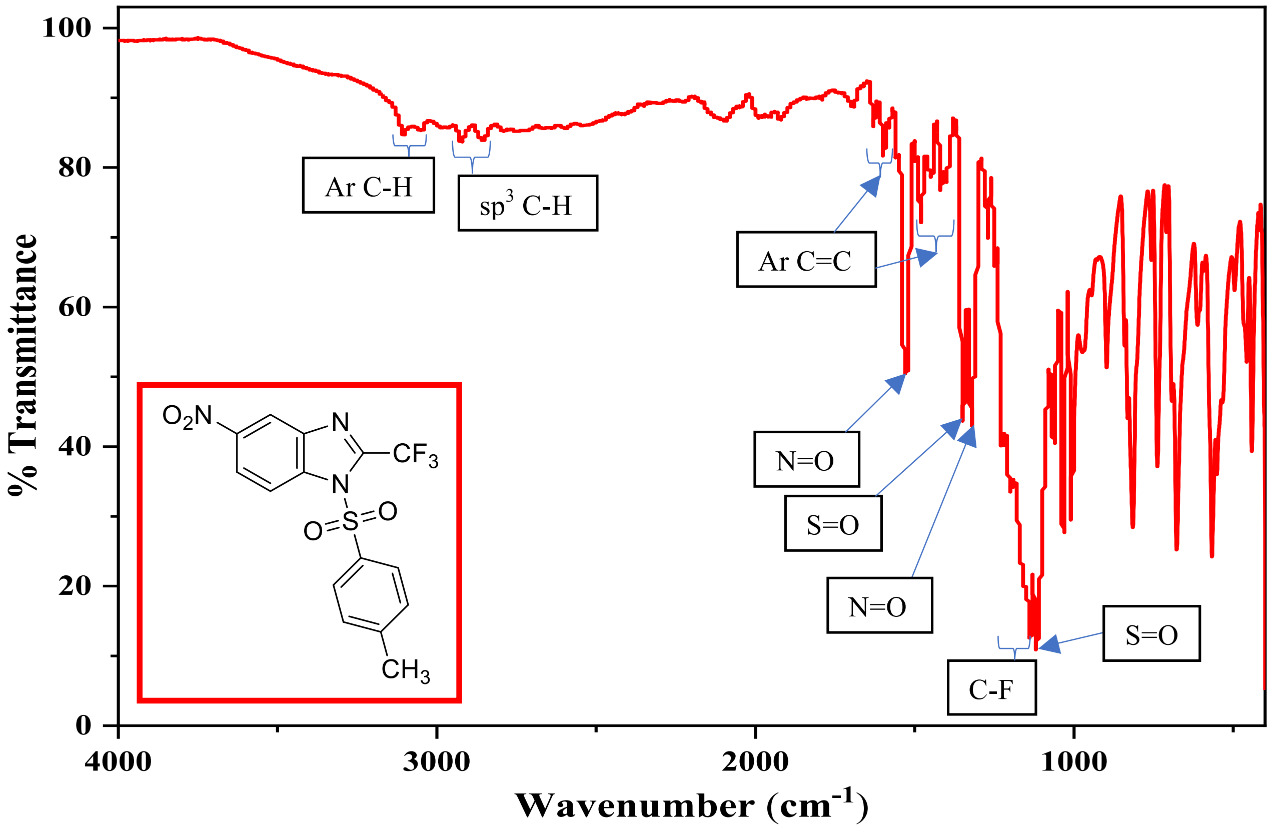

Fig S43: IR Spectrum of compound **2ci**.

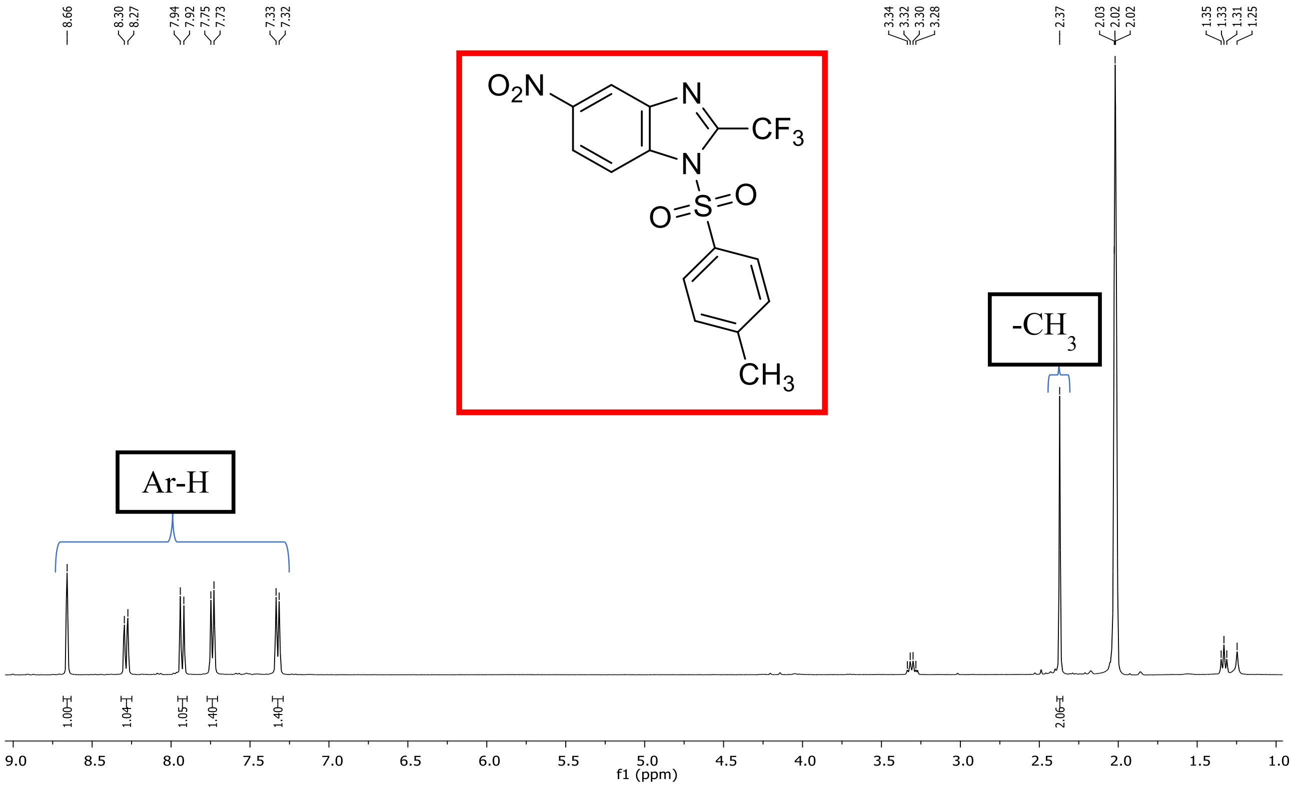

Fig S44: ^{1}H NMR Spectrum of compound **2ci**.

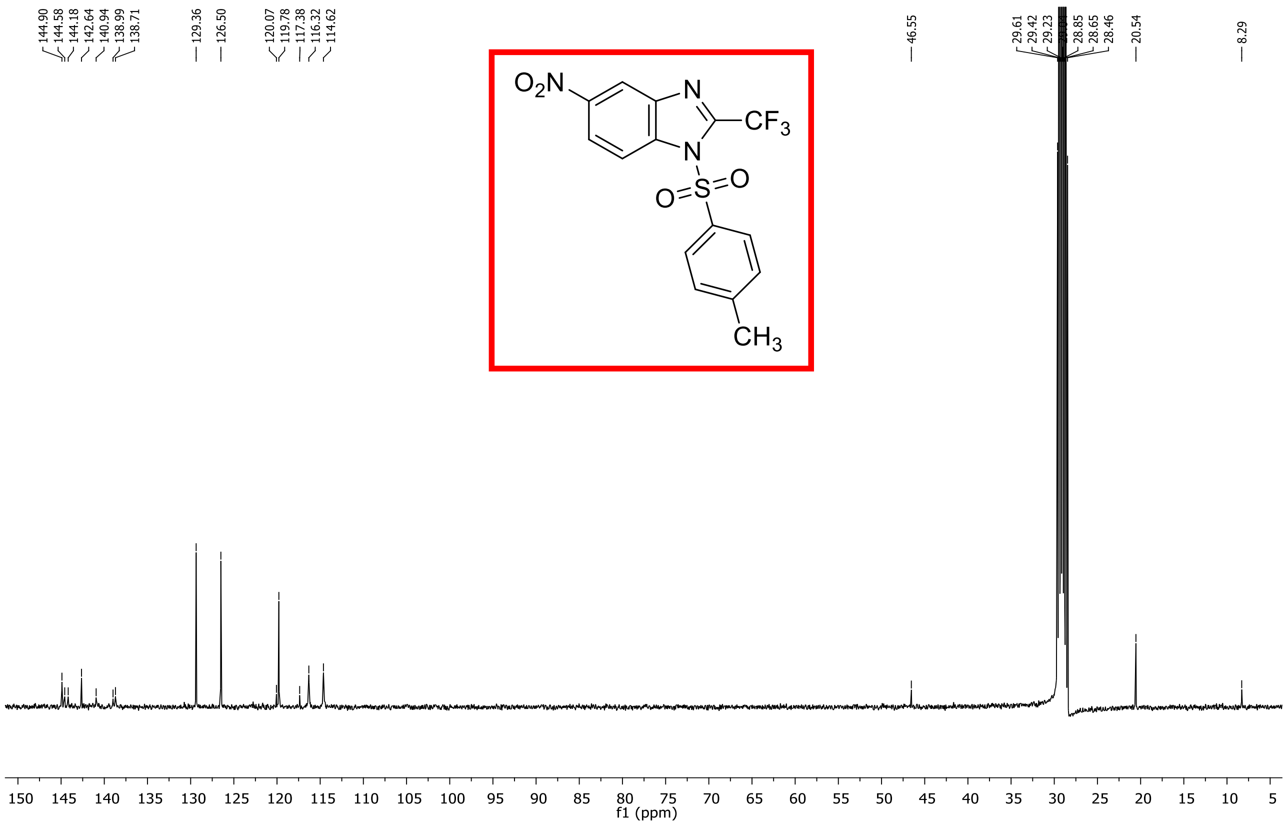

Fig S45: ^{13}C NMR Spectrum of compound **2ci**.

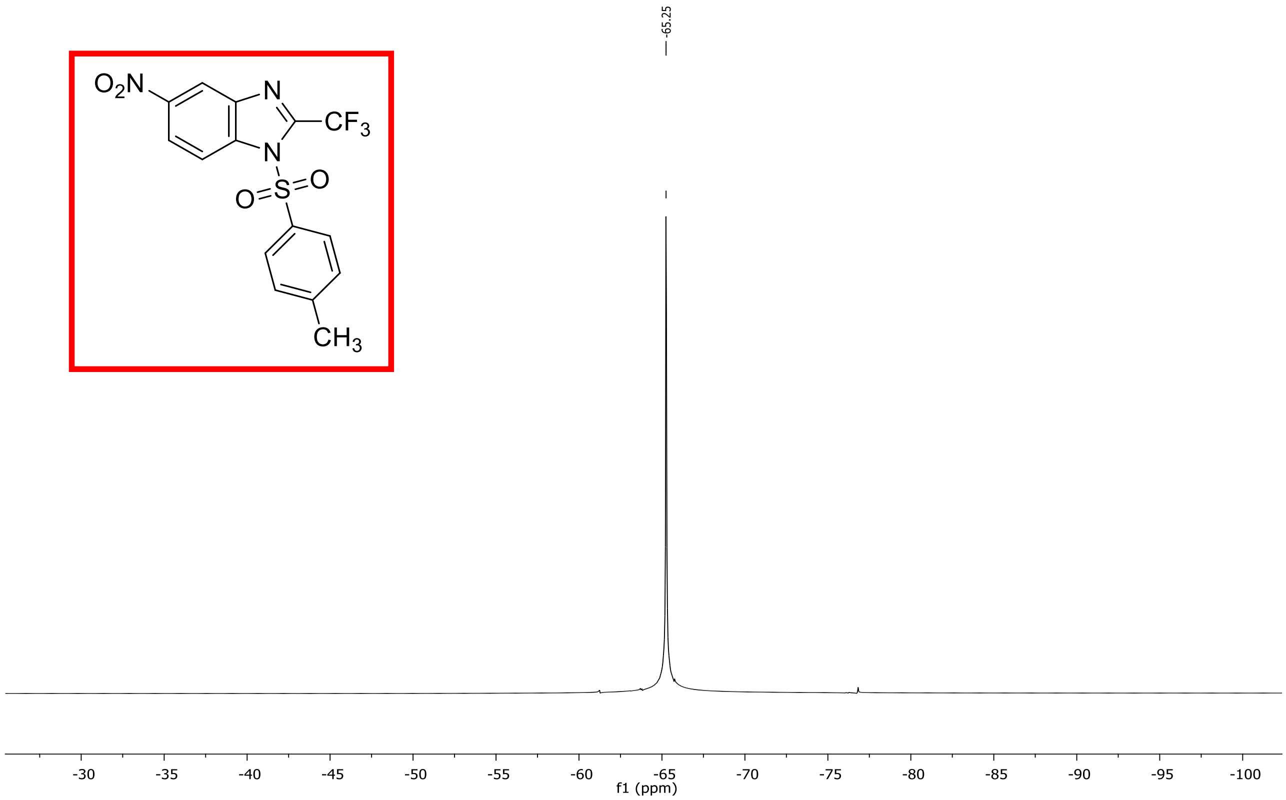

Fig S46: ^{19}F NMR Spectrum of compound **2ci**.

10. IR and NMR Spectra of compound **2cj:**

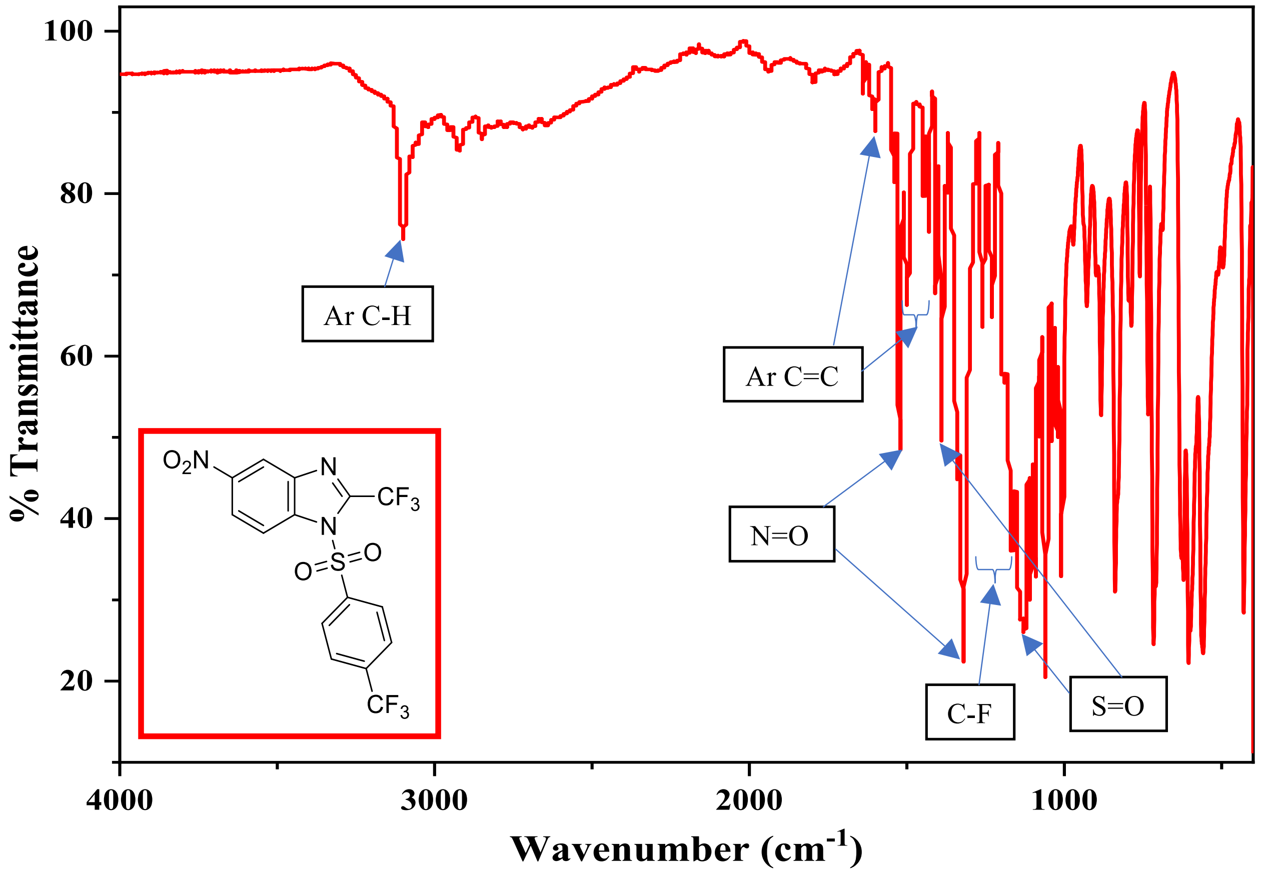

Fig S47: IR Spectrum of compound **2cj**.

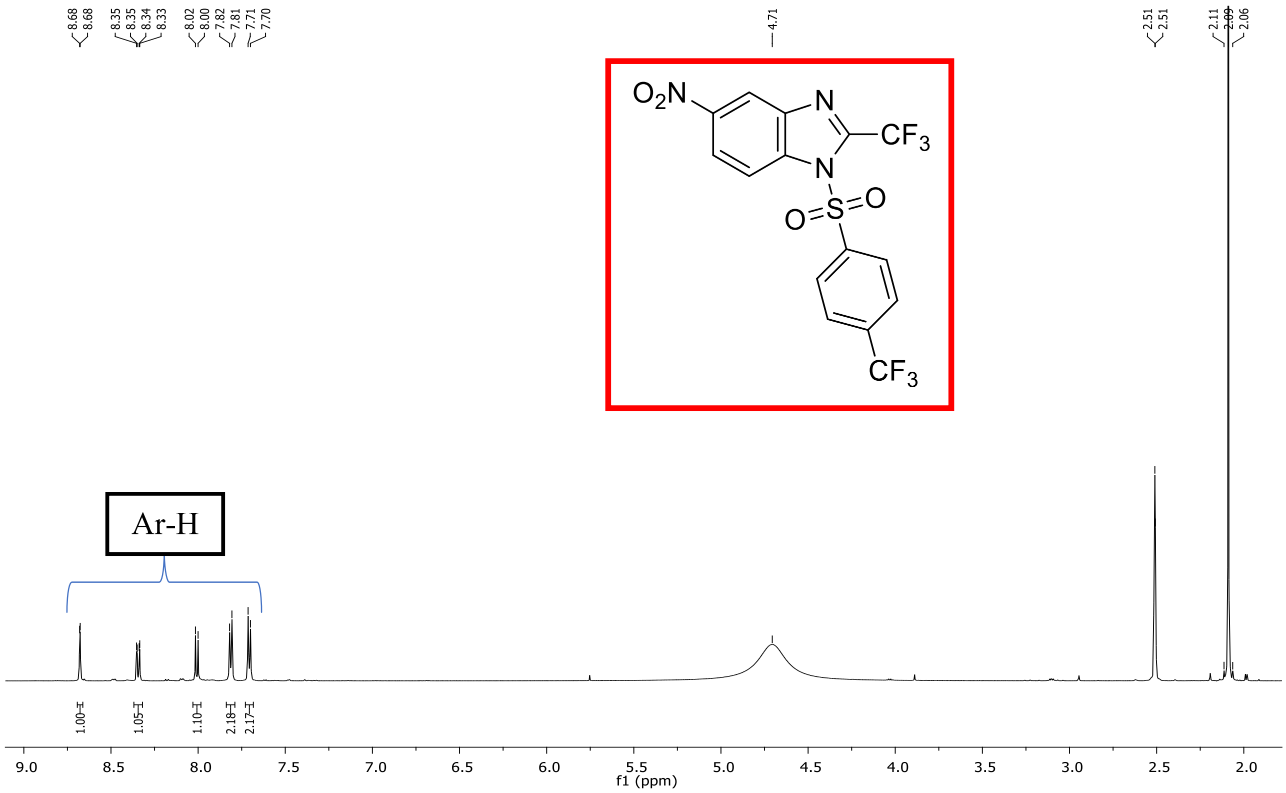

Fig S48: ^{1}H NMR Spectrum of compound **2cj**.

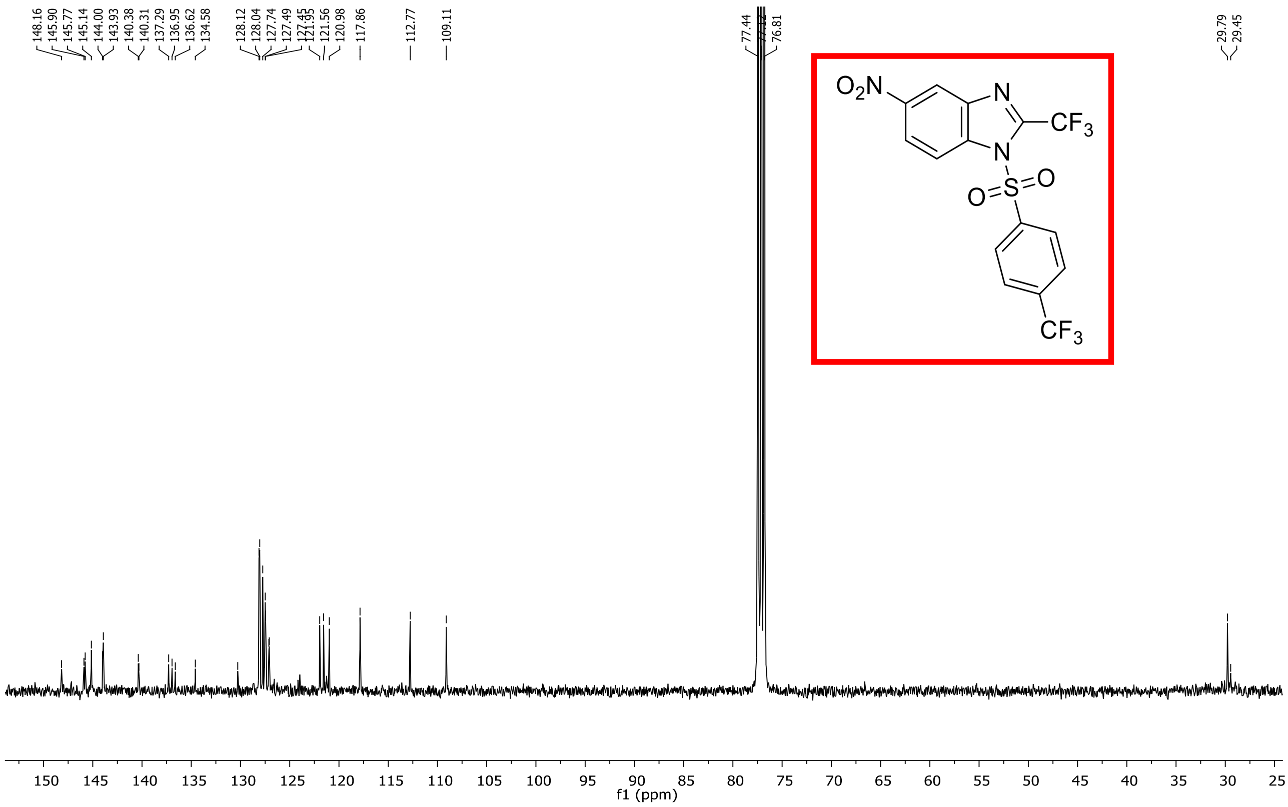

Fig S49: ^{13}C NMR Spectrum of compound **2cj**.

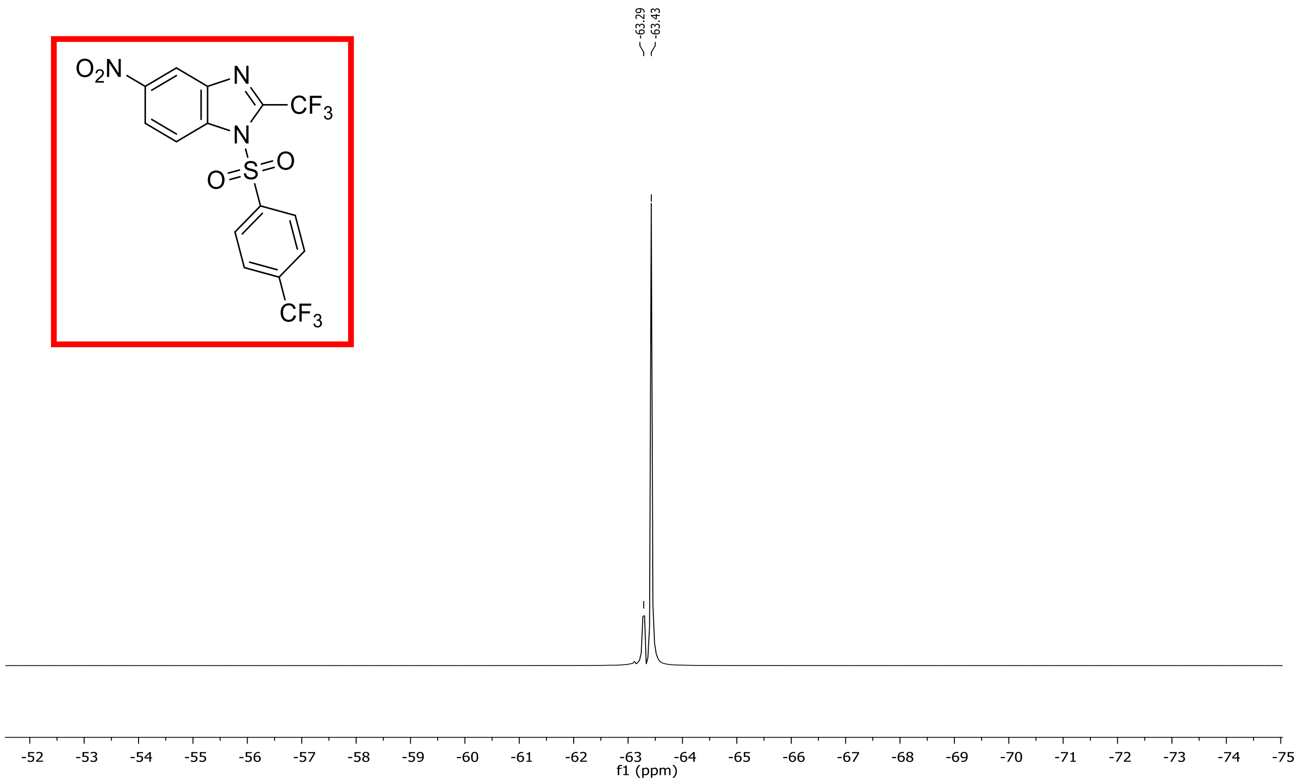

Fig S50: ^{19}F NMR Spectrum of compound **2cj**.

11. IR, NMR and Mass Spectra of compound **3aj:**

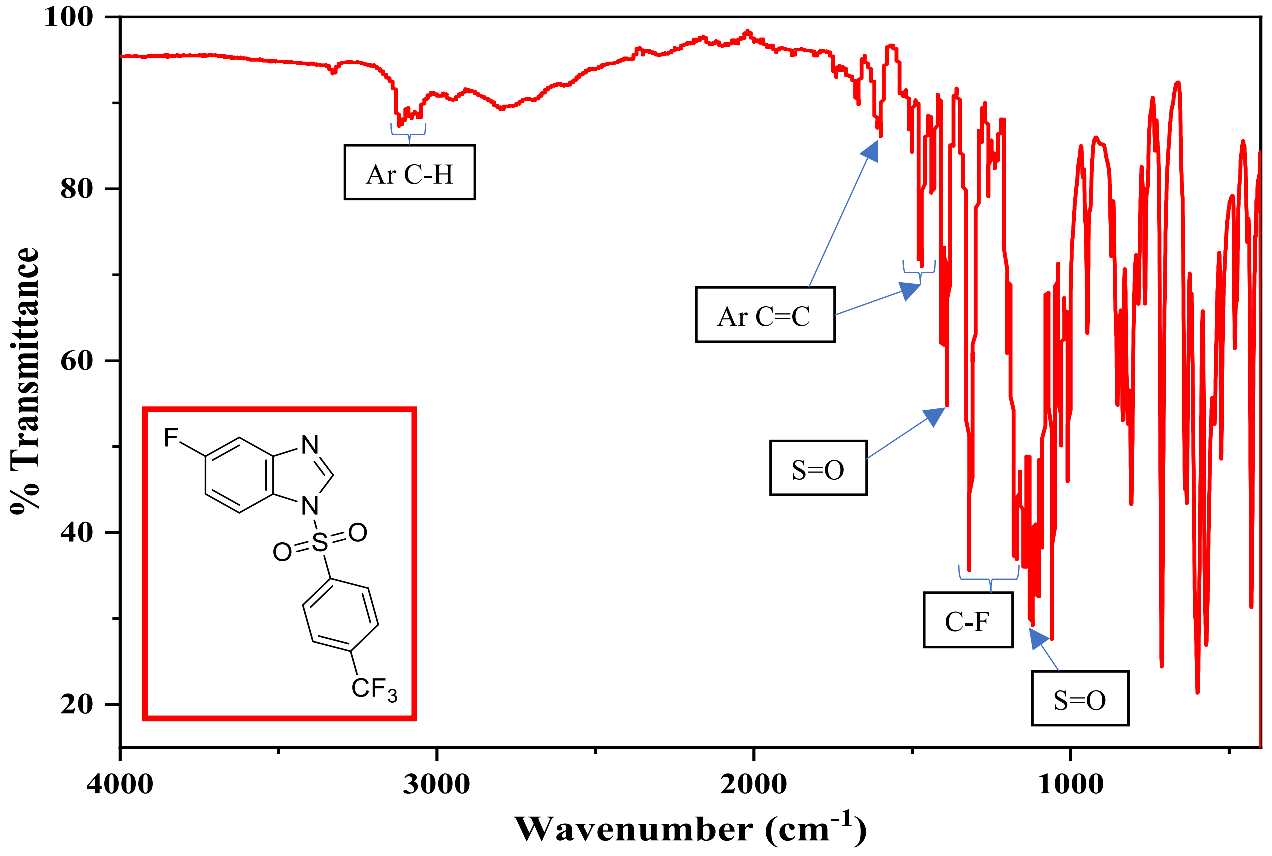

Fig S51: IR Spectrum of compound **3aj**.

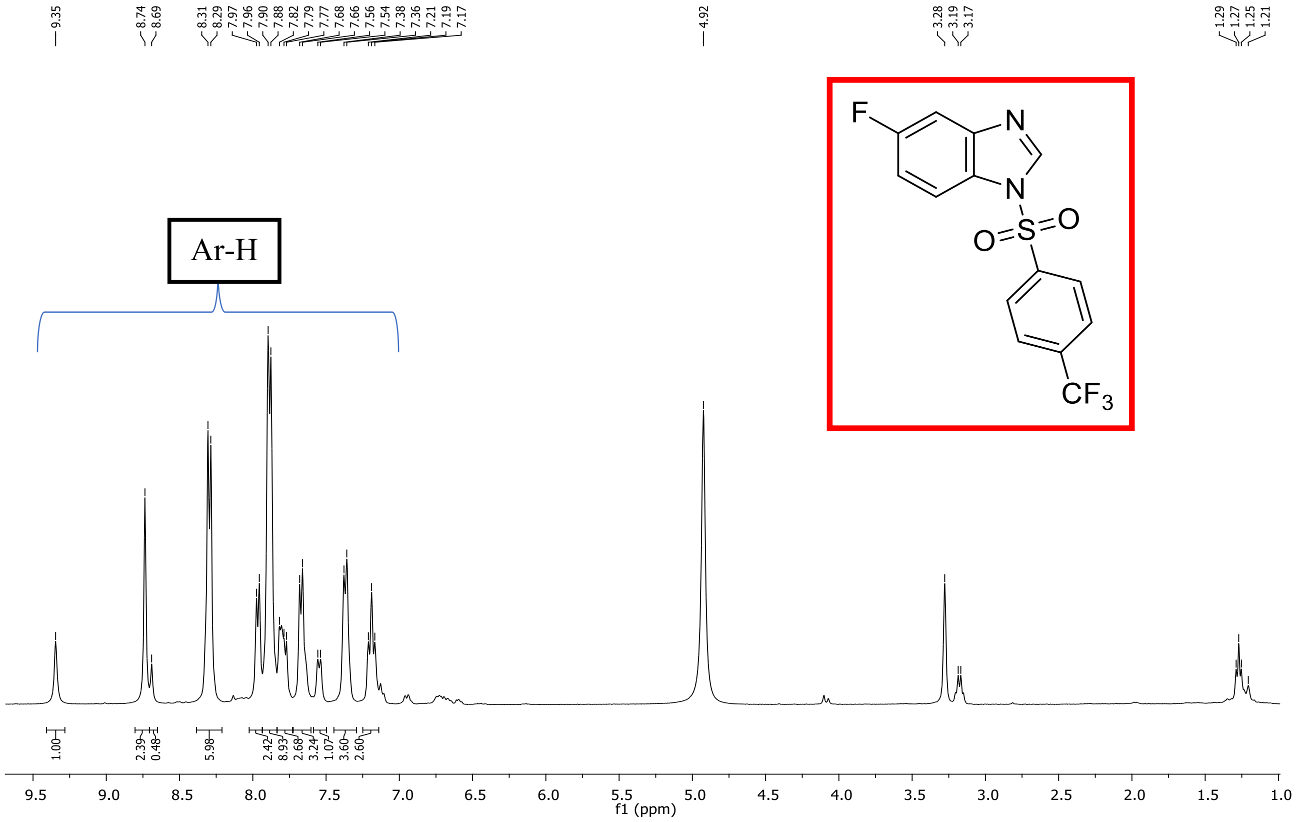

Fig S52: ^{1}H NMR Spectrum of compound **3aj**.

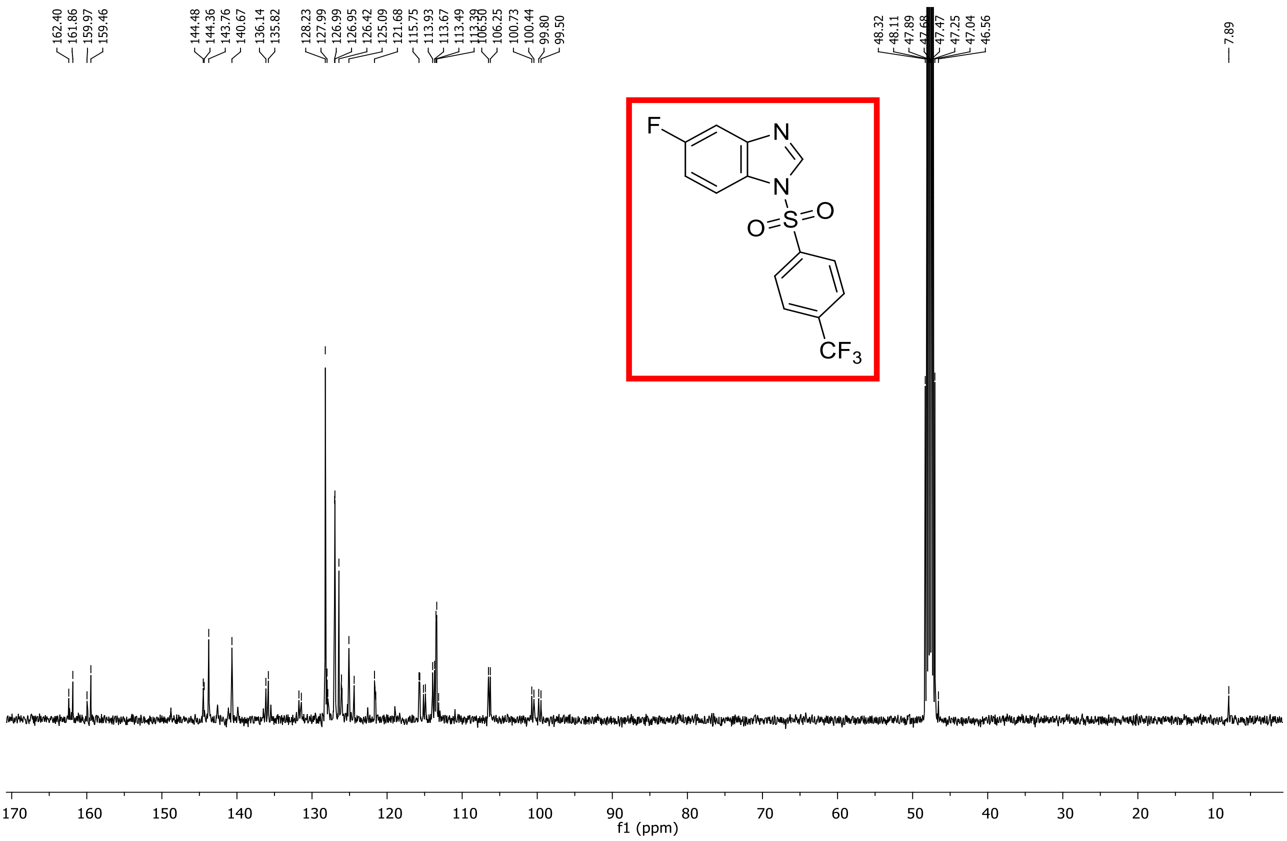

Fig S53: ^{13}C NMR Spectrum of compound **3aj**.

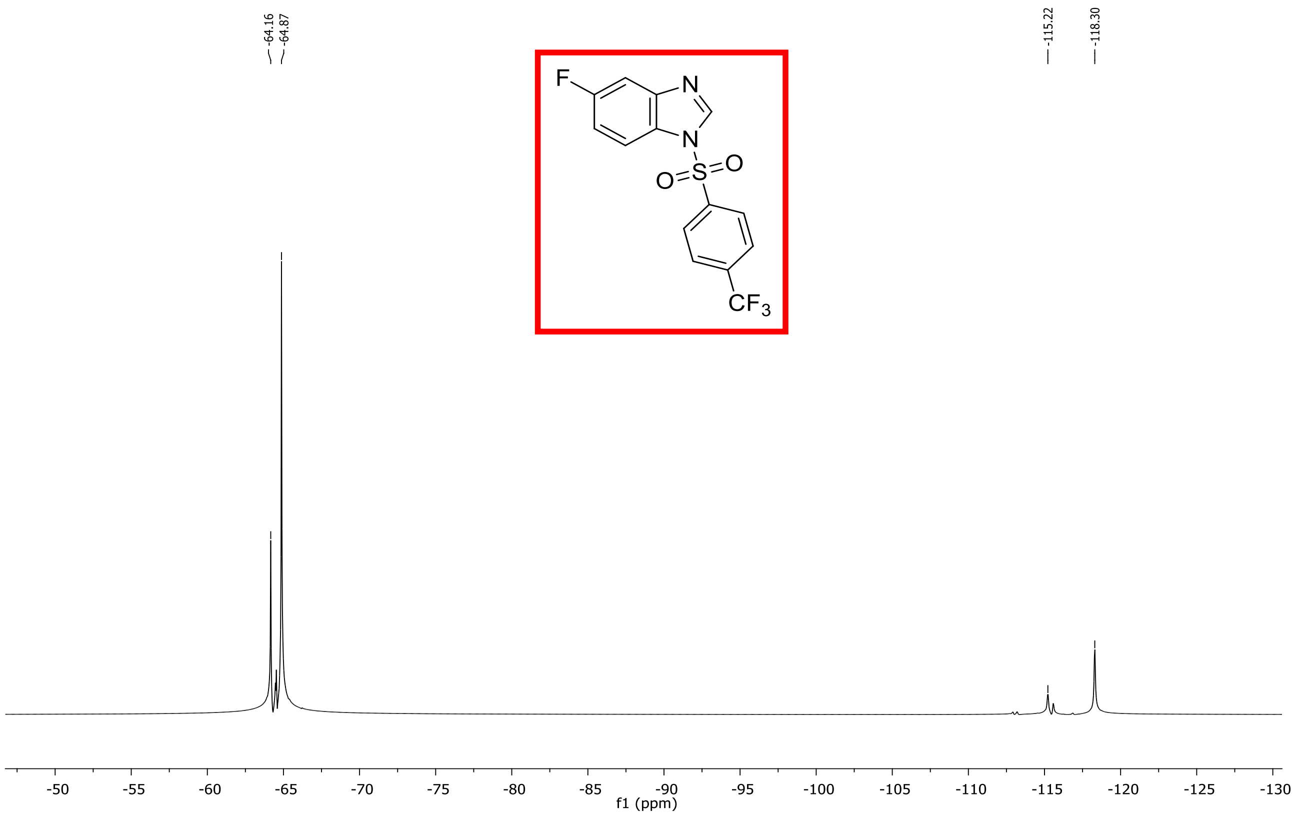

Fig S54: ^{19}F NMR Spectrum of compound **3aj**.

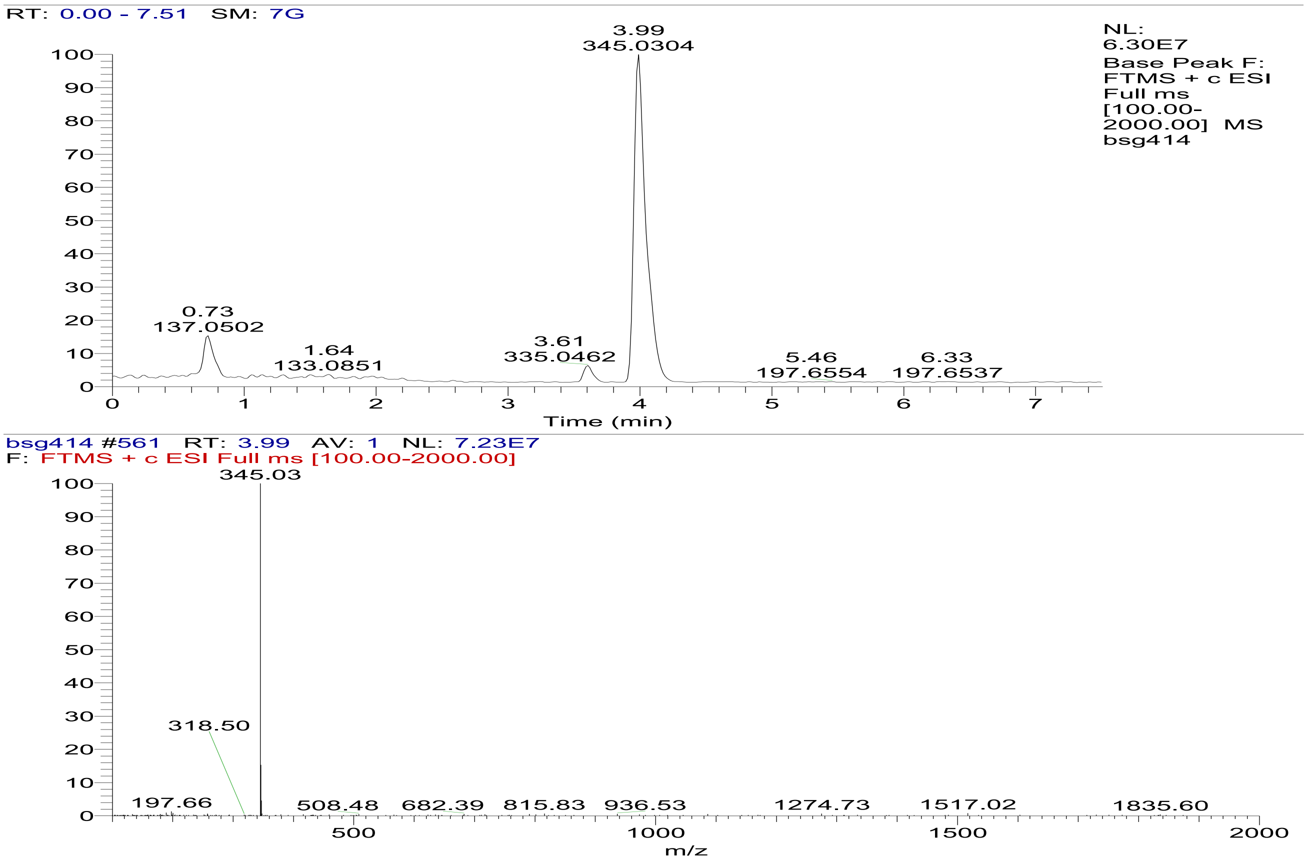

Fig S55: Mass Spectrum of compound **3aj**.

12. IR and NMR Spectra of compound **3bj**:

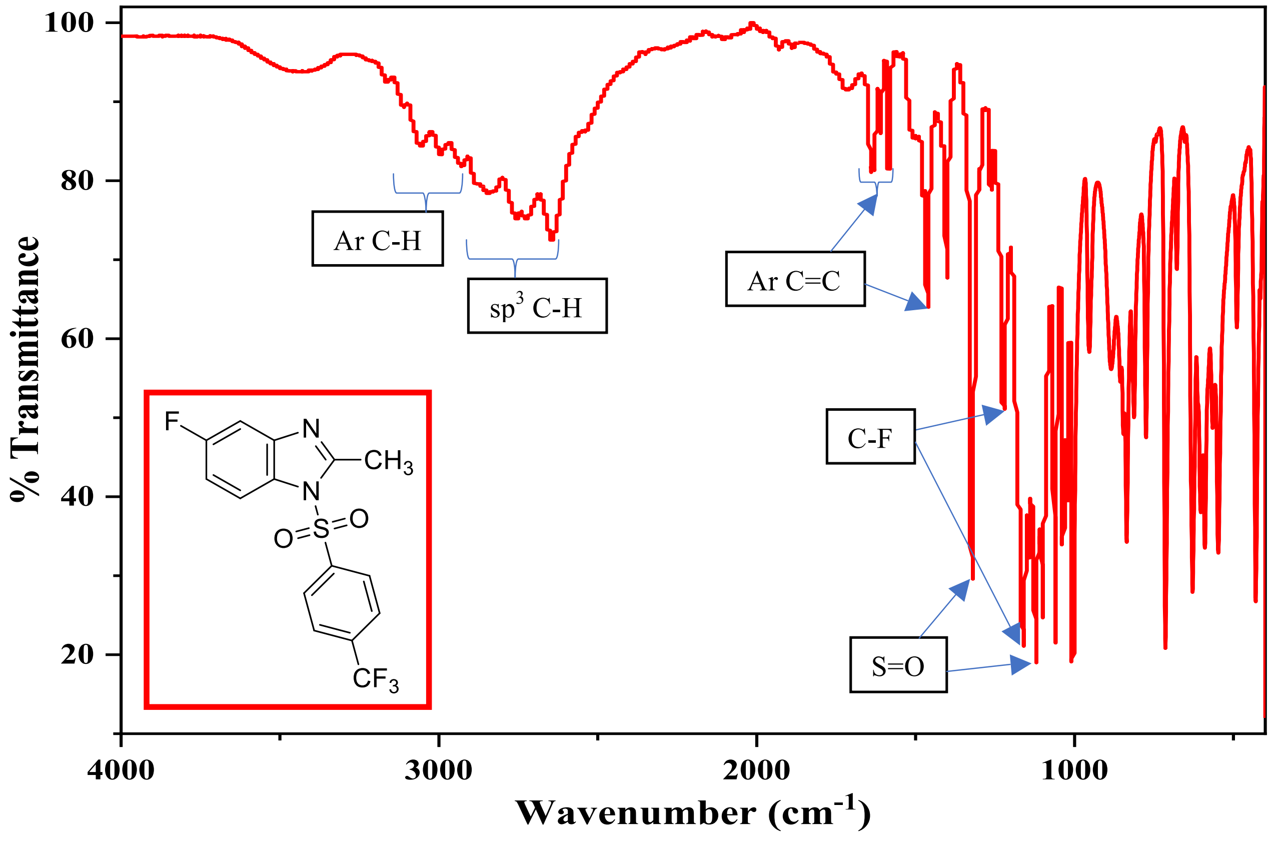

Fig S56: IR and of compound **3bj**.

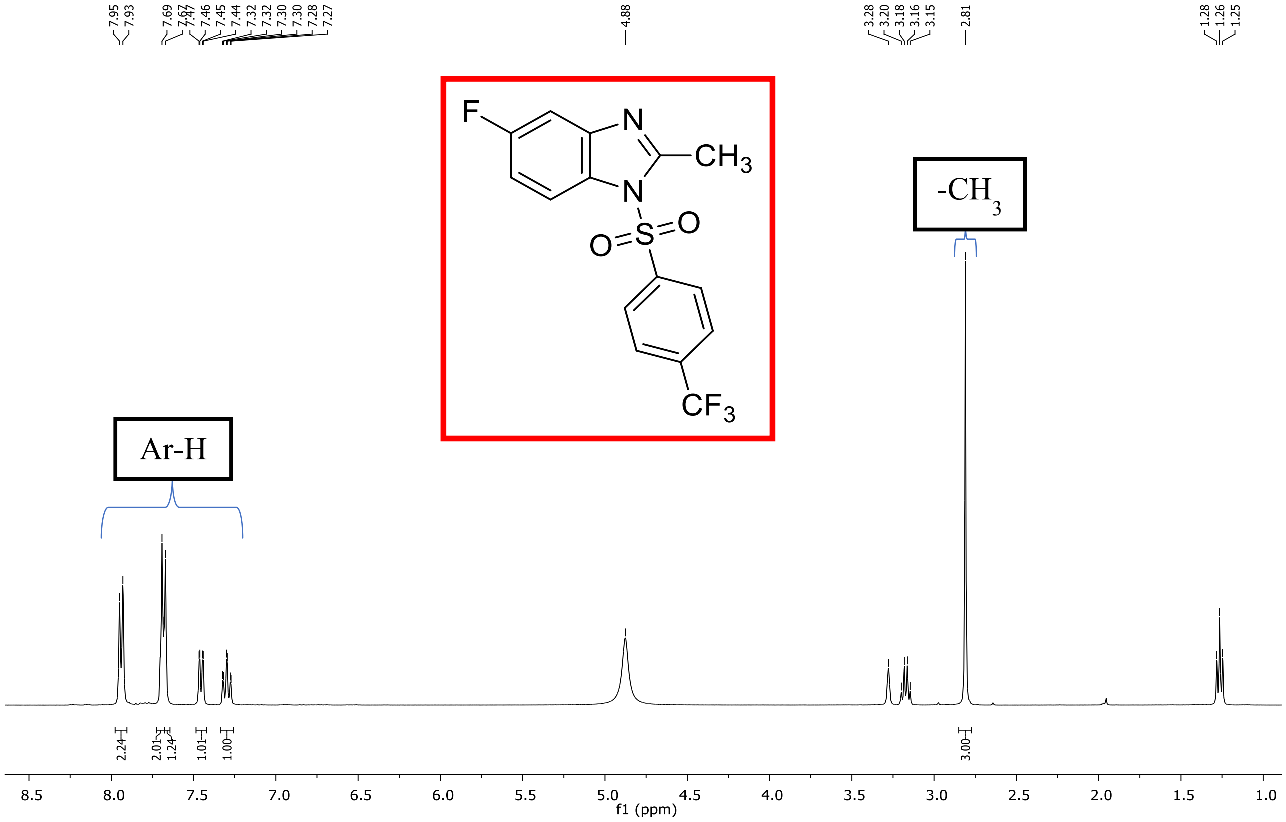

Fig S57: ^{1}H NMR Spectrum of compound **3bj**.

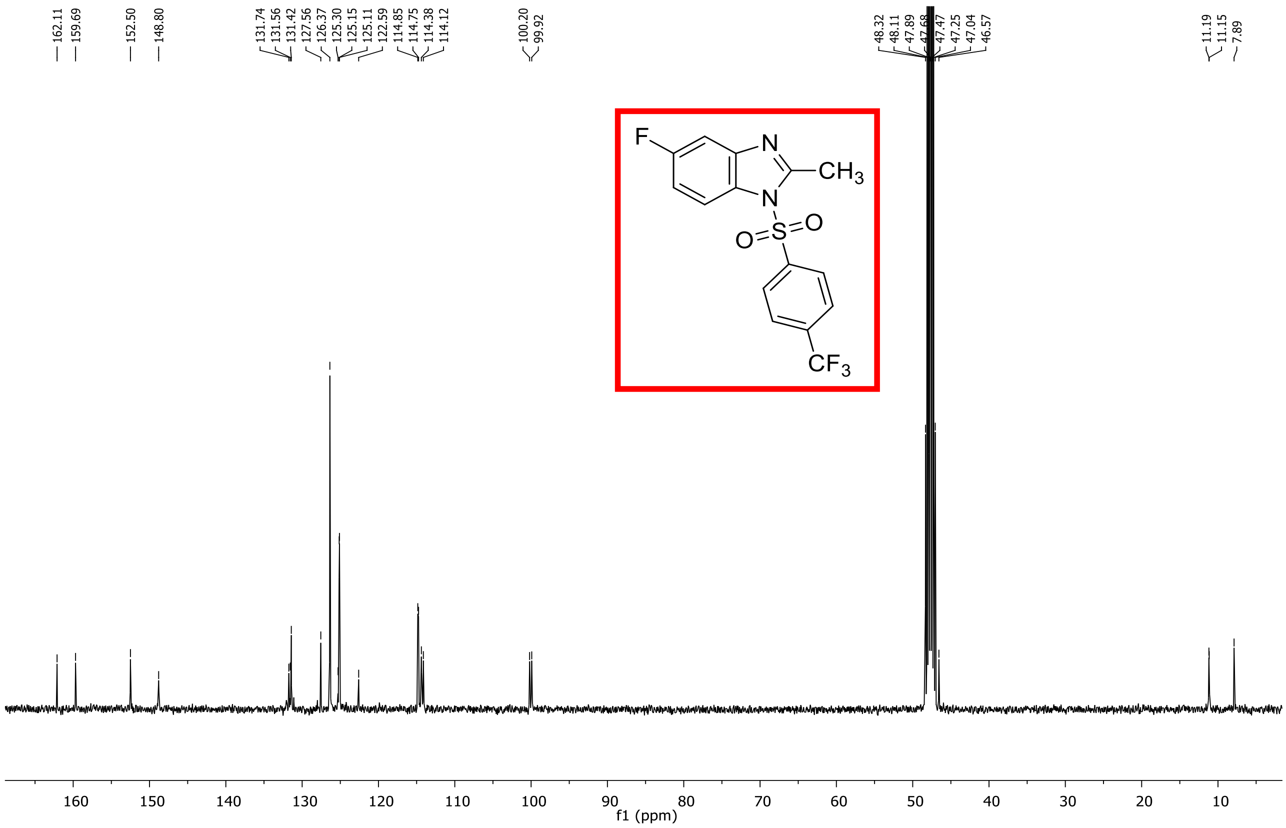

Fig S58: ^{13}C NMR Spectrum of compound **3bj**.

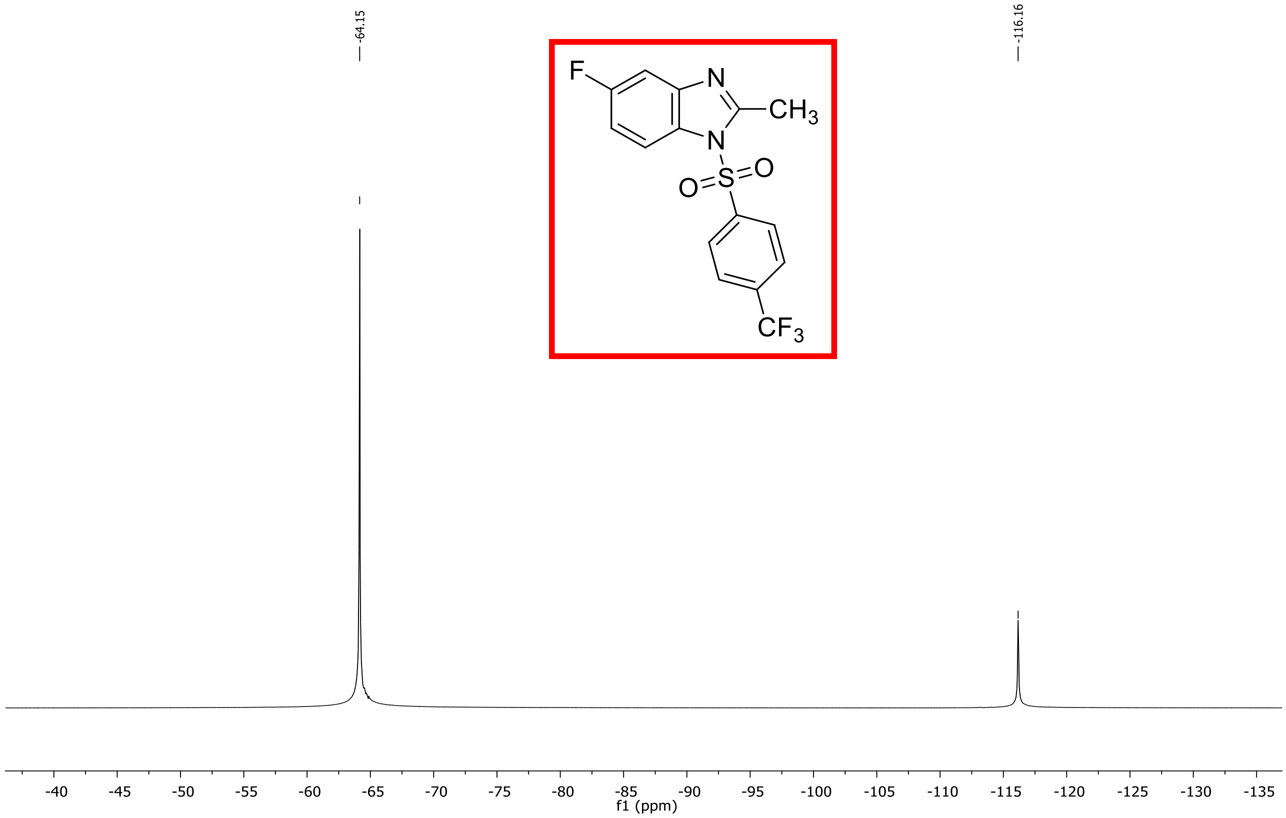

Fig S59: ^{19}F NMR Spectrum of compound **3bj**.

Analysis of Docking Modes of 1ag-3bk:

Table S1: Interacting residues and the types of interactions (ADV result)

Ligand	Interacting residue	Interaction
**1ag**	SER-59, TYR-60	Conventional Hydrogen Bond
	GLY-58	van der Waals
	CYS-397	π-Sulfur
	GLY-57	Amide-π stacked
	VAL-294, LYS-296, CYS-397	Alkyl
	TRP-388	π-Alkyl
**1ah**	SER-59, TYR-60	Conventional Hydrogen Bond
	GLY-58	van der Waals
	TRP-388	π-σ
	CYS-397, TYR-60	π-Sulfur
	TYR-435	π-π stacked
	GLY-57	Amide-π stacked

Ligand	Interacting residue	Interaction
	TRP-388	π-Alkyl
	VAL-294, LYS-296, CYS-397	Alkyl
**1ai**	SER-59, TYR-60	Conventional Hydrogen Bond
	GLY-58	van der Waals
	CYS-397, TYR-60	π-Sulfur
	TYR-435	π-π stacked
	GLY-57	Amide-π stacked
	VAL-294, LYS-296, CYS-397	Alkyl
	TRP-388	π-σ
	TRP-388, TYR-398	π-Alkyl
**1aj**	SER-59, TYR-60	Conventional Hydrogen Bond
	GLY-58	van der Waals
	CYS-172	Carbon Hydrogen Bond
	CYS-172, LEU-171, GLN-206	Halogen (Fluorine)
	CYS-397	π-Sulfur
	TYR-435	π-π stacked
	GLY-57	Amide-π stacked
	LEU-171, VAL-294, CYS-397, LYS-296	Alkyl
	TRP-388, TYR-398	π-Alkyl
**1ak**	SER-59, TYR-60	Conventional Hydrogen Bond
	GLY-58	van der Waals
	TRP-388	π-σ
	CYS-397	π-Sulfur
	TYR-435	π-π stacked
	GLY-57	Amide-π stacked
	VAL-294, LYS-296, CYS-397	Alkyl
	TRP-388, TYR-398	π-Alkyl
**1bh**	SER-59, TYR-60	Conventional Hydrogen Bond
	GLY-58	van der Waals

Ligand	Interacting residue	Interaction
	TYR-435	π-σ
	CYS-397	π-Sulfur
	TYR-435	π-π Stacked
	PHE-343	π-π T-shaped
	GLY-57	Amide-π Stacked
	LEU-171	Alkyl
	TYR-326	π-Alkyl
**1bi**	SER-59, TYR-60	Conventional Hydrogen Bond
	GLY-58	van der Waals
	TYR-435	π-σ
	CYS-397	π-Sulfur
	TYR-435	π-π Stacked
	GLY-57	Amide-π Stacked
	LEU-171, VAL-294, CYS-397	Alkyl
	TYR-326, TRP-388, TYR-398	π-Alkyl
**1bj**	SER-59, TYR-60	Conventional Hydrogen Bond
	GLY-58	van der Waals
	GLY-57	Carbon Hydrogen Bond
	LEU-56, GLY-57	Halogen (Fluorine)
	TYR-435	π-σ
	CYS-397	π-Sulfur
	TYR-435	π-π stacked
	PHE-343	π-π T-shaped
	GLY-57	Amide-π stacked
	LEU-171, VAL-294, CYS-397, LYS-296	Alkyl
	TYR-326, TRP-388, TYR-398	π-Alkyl
**1bk**	SER-59, TYR-60	Conventional Hydrogen Bond
	GLY-58	van der Waals
	CYS-397	π-Sulfur
	TYR-435	π-σ
	TYR-435	π-π stacked
	GLY-57	Amide-π stacked

Ligand	Interacting residue	Interaction
	LEU-171, VAL-294, CYS-397, LYS-296	Alkyl
	PHE-343	π-π T-shaped
	TYR-326, PHE-343, TRP-388	π-Alkyl
**2ai**	SER-59, TYR-60	Conventional Hydrogen Bond
	GLY-58	van der Waals
	CYS-397	π-Sulfur
	TYR-435	π-π stacked
	GLY-57	Amide-π stacked
	VAL-294, CYS-397, LYS-296	Alkyl
	TYR-398, TRP-388	π-Alkyl
**2aj**	SER-59, TYR-60, TYR-388	Conventional Hydrogen Bond
	GLY-58	van der Waals
	GLY-57	Carbon Hydrogen Bond
	GLY-57, LEU-56, TYR-388	Halogen (Fluorine)
	CYS-397	π-Sulfur
	TYR-435, TYR-398	π-π stacked
	GLY-57	Amide-π stacked
	VAL-294, CYS-397, LYS-296	Alkyl
	TRP-388, TYR-398, PHE-343	π-Alkyl
**2ak**	SER-59, TYR-60, MET-436, TRP-388	Conventional Hydrogen Bond
	GLY-58	van der Waals
	GLY-57, LEU-171, TYR-398	Carbon Hydrogen Bond
	CYS-397	π-Sulfur
	TYR-435	π-π stacked
	GLY-57	Amide-π stacked
	TYR-398	π-π T-shaped
	LEU-171	Alkyl
	PHE-343, TYR-398	π-Alkyl
**2bi**	SER-59, TYR-60	Conventional Hydrogen Bond
	GLY-58	van der Waals
	CYS-397	π-Sulfur

Ligand	Interacting residue	Interaction
	TYR-435	π-σ
	TYR-435	π-π stacked
	GLY-57	Amide-π stacked
	VAL-294, CYS-397	Alkyl
	TRP-388, TYR-398	π-Alkyl
**2bj**	SER-59, TYR-60	Conventional Hydrogen Bond
	GLY-58	van der Waals
	GLY-57	Carbon Hydrogen Bond
	GLY-57, LEU-56	Halogen (Fluorine)
	CYS-397	π-Sulfur
	TYR-435	π-σ
	TYR-435	π-π stacked
	GLY-57	Amide-π stacked
	VAL-294, CYS-397, LYS-296	Alkyl
	PHE-343	π-π T-shaped
	TRP-388, TYR-398	π-Alkyl
**2ci**	SER-59, TYR-60	Conventional Hydrogen Bond
	GLY-58	van der Waals
	CYS-397	π-Sulfur
	GLY-434	Halogen (Fluorine)
	TYR-435, TYR-398	π-π stacked
	GLY-57	Amide-π stacked
	VAL-294, LYS-296, CYS-397	Alkyl
	TRP-388, TYR-398, TYR-435	π-Alkyl
**2cj**	SER-59, TYR-60, TRP-388	Conventional Hydrogen Bond
	GLY-58	van der Waals
	GLY-57	Carbon Hydrogen Bond
	GLY-57, LEU-56, GLY-434	Halogen (Fluorine)
	CYS-397	π-Sulfur
	TYR-435, TYR-398	π-π stacked
	GLY-57	Amide-π stacked
	VAL-294, CYS-397, LYS-296	Alkyl

Ligand	Interacting residue	Interaction
	TRP-388, TYR-398, TYR-435, PHE-343	π-Alkyl
**3aj**	SER-59, TYR-60	Conventional Hydrogen Bond
	GLY-58	van der Waals
	CYS-172	Carbon Hydrogen Bond
	LEU-171, CYS-172, GLN-206	Halogen (Fluorine)
	CYS-397	π-Sulfur
	TYR-435	π-π stacked
	GLY-57	Amide-π stacked
	LEU-171	Alkyl
	TYR-398	π-Alkyl
**3ak**	SER-59, TYR-60	Conventional Hydrogen Bond
	GLY-58	van der Waals
	CYS-397, TYR-60	π-Sulfur
	TYR-398, TYR-435	π-π stacked
	GLY-57	Amide-π stacked
	TYR-398	π-Alkyl
**3bj**	SER-59, TYR-60	Conventional Hydrogen Bond
	GLY-58	van der Waals
	GLY-57	Carbon Hydrogen Bond
	GLY-57, LEU-56	Halogen (Fluorine)
	CYS-397	π-Sulfur
	TYR-435	π-σ
	TYR-435	π-π stacked
	GLY-57	Amide-π stacked
	VAL-294, CYS-397, LYS-296	Alkyl
	PHE-343	π-π T-shaped
	TRP-388, TYR-398	π-Alkyl
**3bk**	SER-59, TYR-60	Conventional Hydrogen Bond
	GLY-58	van der Waals
	TYR-435	π-σ
	CYS-397	π-Sulfur
	TYR-435	π-π stacked

Ligand	Interacting residue	Interaction
	GLY-57	Amide-π stacked
	PHE-343	π-π T-shaped
	VAL-294, CYS-397, LYS-296	Alkyl
	TRP-388, PHE-343	π-Alkyl
**II**	GLY-58	van der Waals
	GLY-434	Carbon Hydrogen Bond
	CYS-397	π-Sulfur
	TYR-398	π-π T-shaped
	GLY-57	Amide-π stacked
	ARG-42	Alkyl
	LYS-296	π-alkyl
**III**	TYR-435	π-σ
	LEU-171	Alkyl
	LEU-171, PHE-343, TYR-398	π-alkyl
**V**	TYR-60, GLN-206, LEU-171	Conventional Hydrogen Bond
	TYR-435	Carbon Hydrogen Bond
	TYR-435	π-π stacked
	TYR-398	π-π T-shaped
**VI**	GLY-58	van der Waals
	TYR-60	Carbon Hydrogen Bond
	CYS-397	π-Sulfur
	TYR-435	π-σ
	GLY-57	Amide-π stacked
	TYR-60, TYR-435	π-alkyl
**VII**	GLY-57	Conventional Hydrogen Bond
	TYR-398	π-π stacked
	VAL-294, CYS-397, LYS-296	Alkyl
	TRP-388, TYR-398, PHE-343	π-alkyl
**VIII**	GLN-206, TYR-435, CYS-172	Conventional Hydrogen Bond
	CYS-172	Carbon Hydrogen Bond
	TYR-398	Unfavorable Acceptor-Acceptor
	CYS-172	π-Sulfur
	LEU-171, ILE-199	π-σ

Ligand	Interacting residue	Interaction
	PRO-104, LEU-164	Alkyl
	TRP-119, LEU-171, ILE-198, ILE-316	π-alkyl

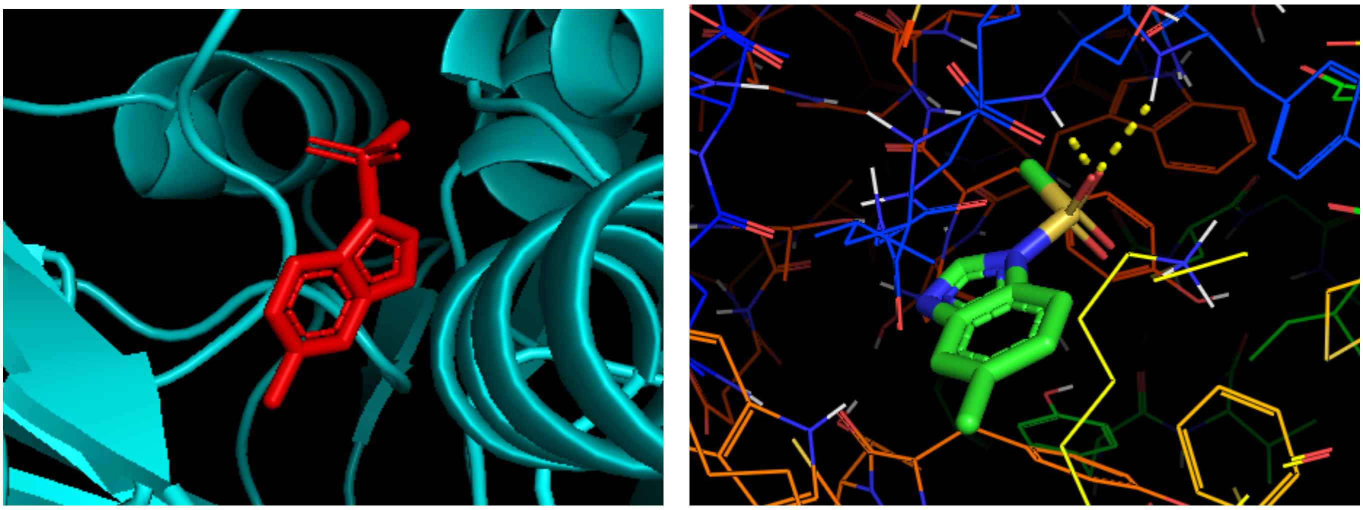

1ag

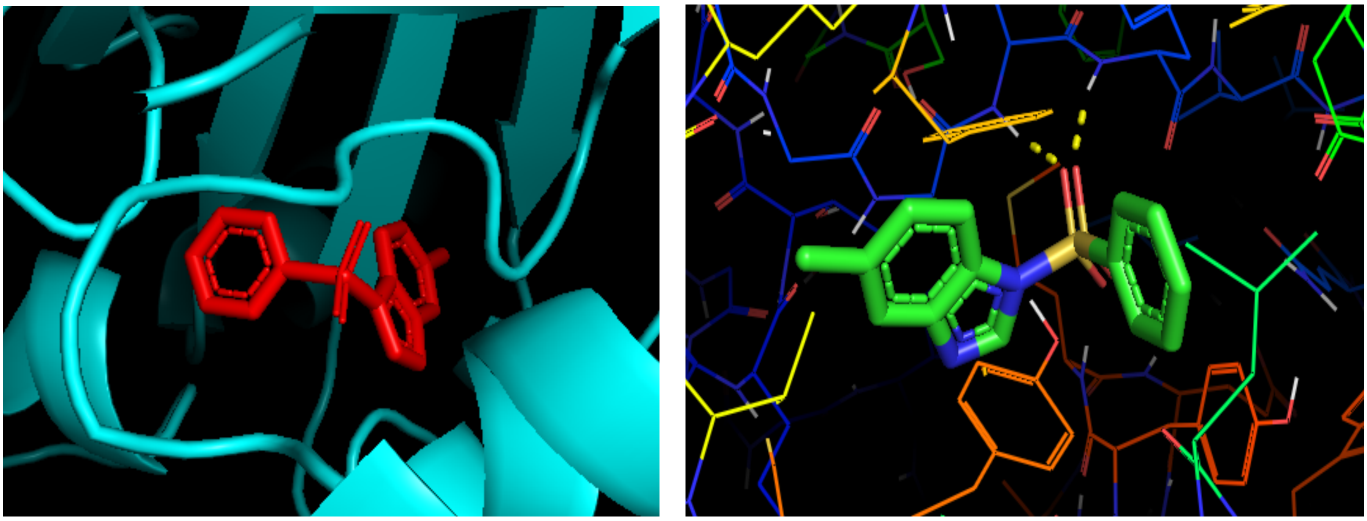

1ah

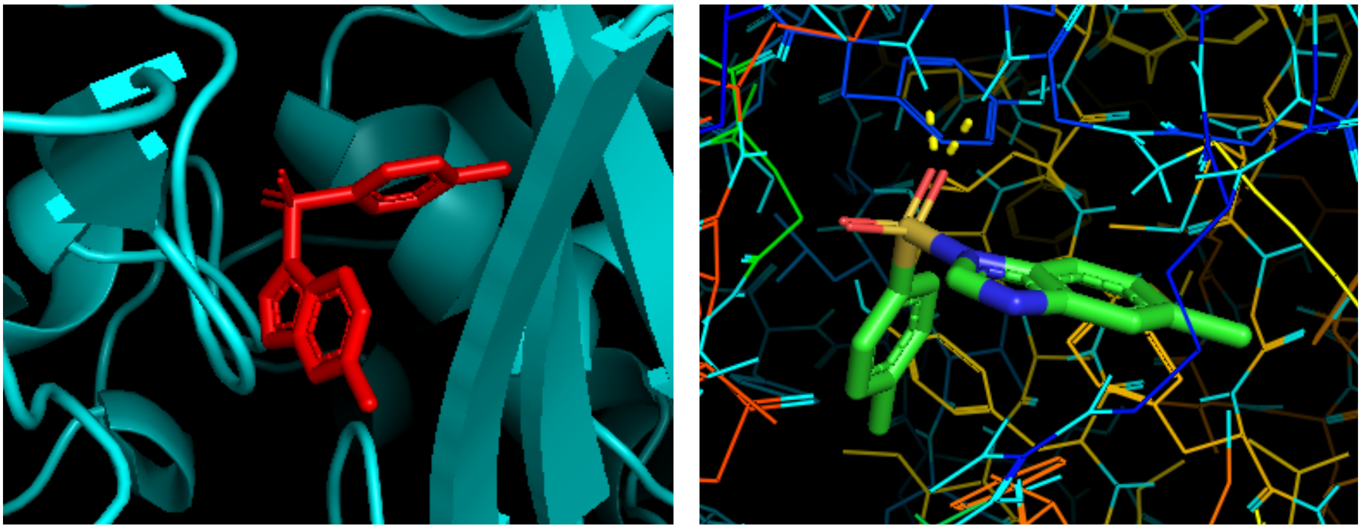

1ai

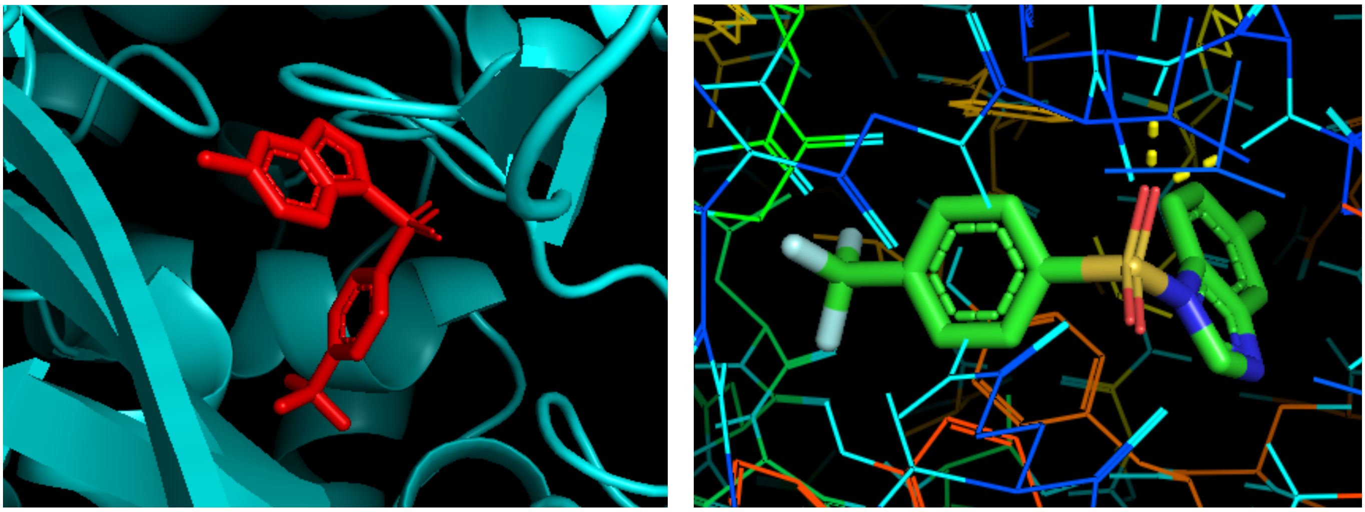

1aj

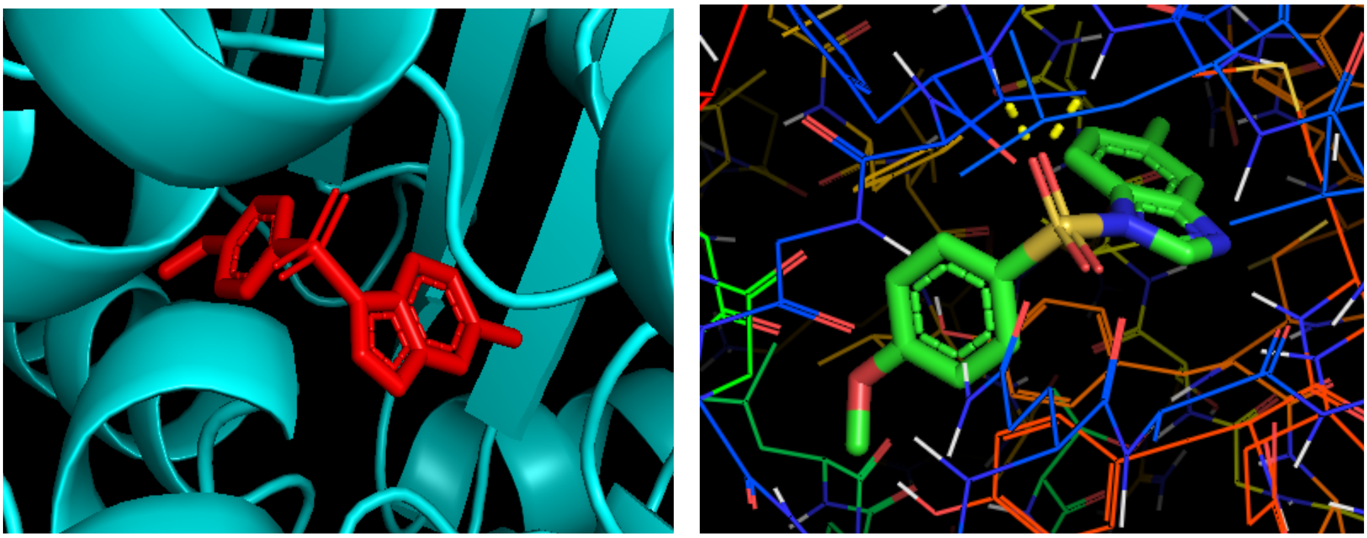

1ak

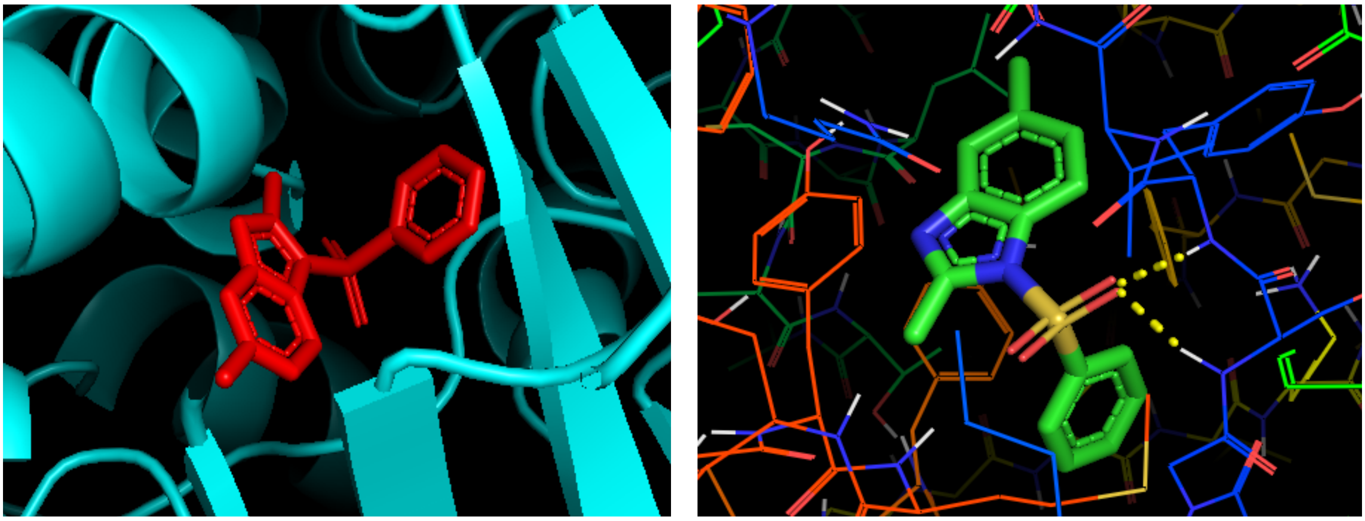

1bh

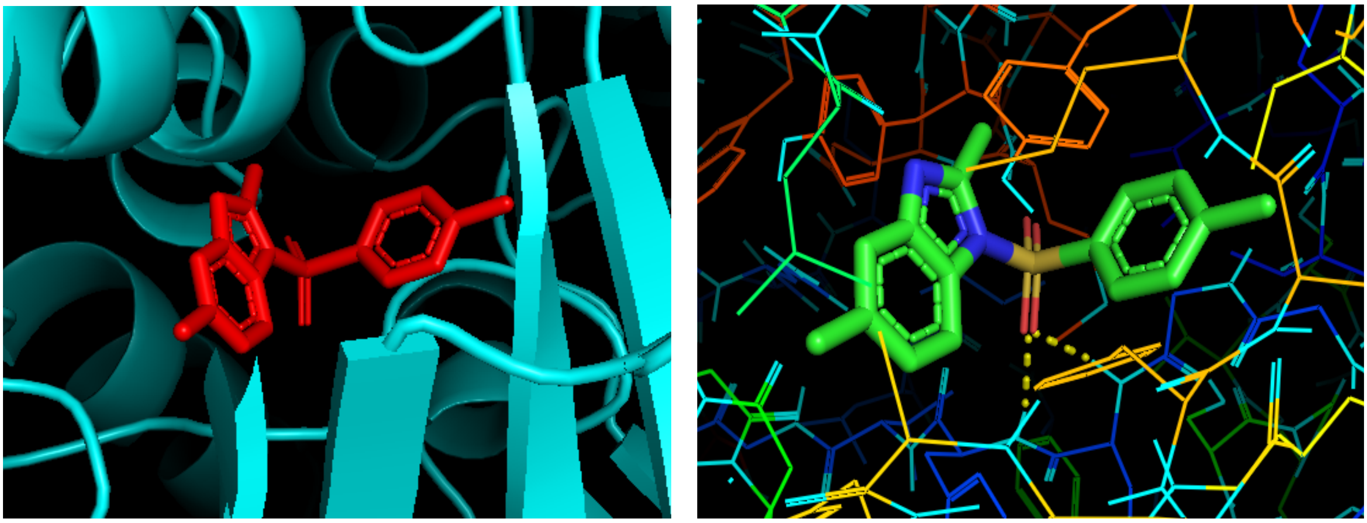

1bi

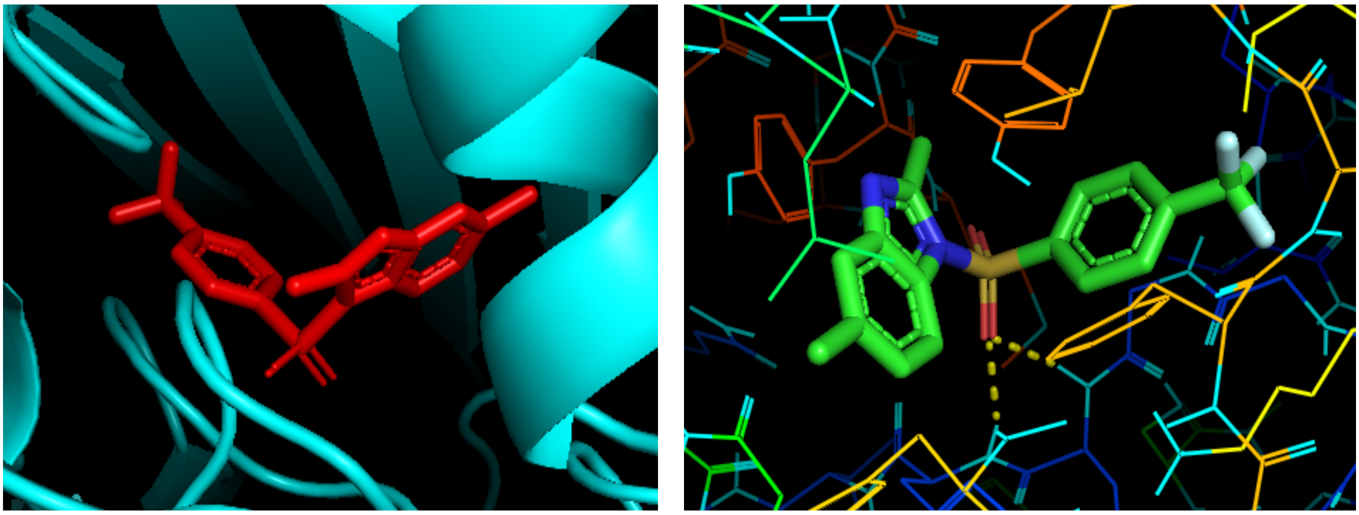

1bj

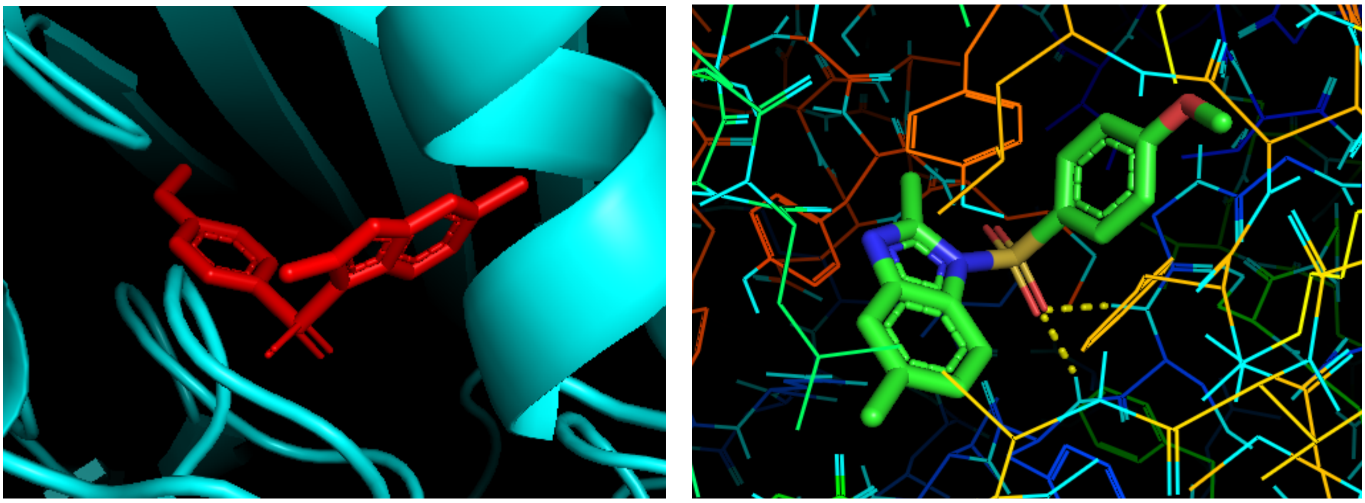

1bk

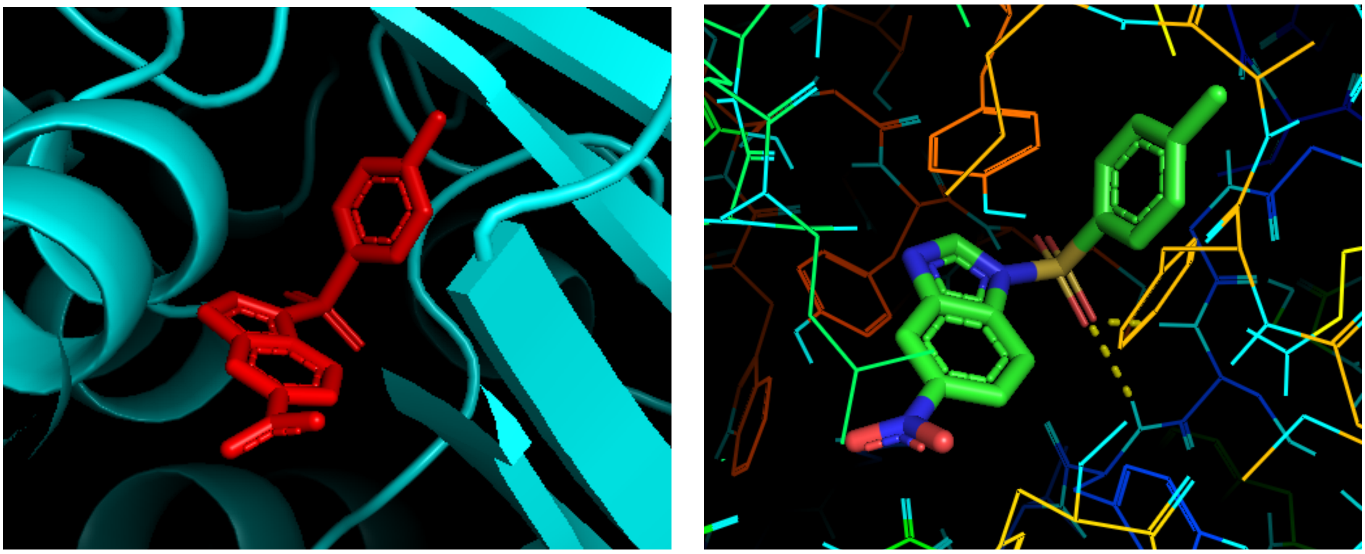

2ai

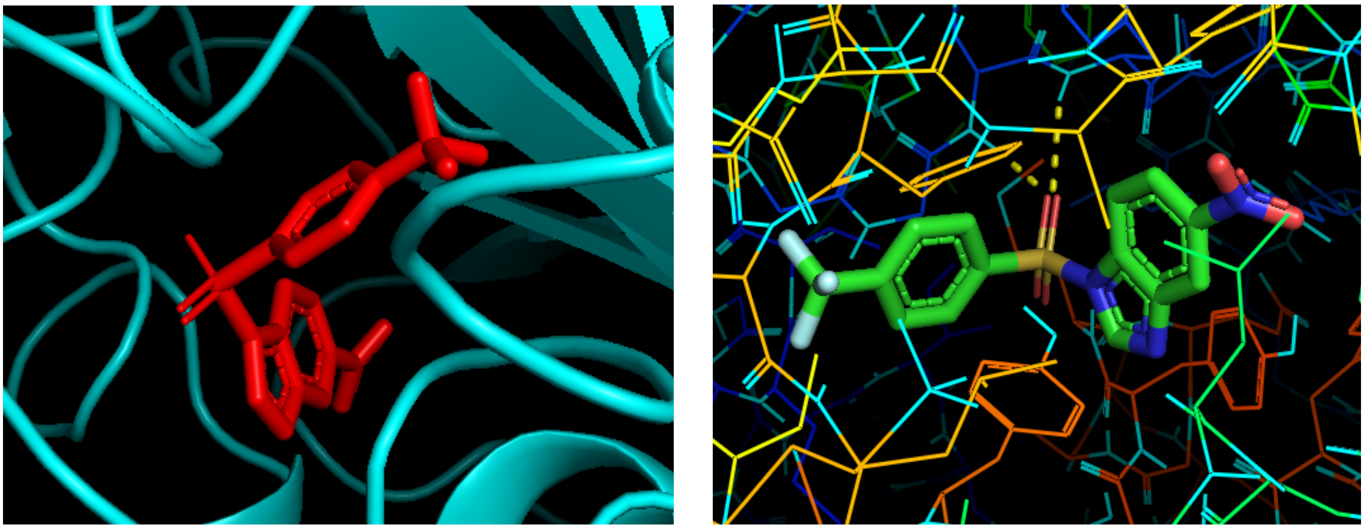

2aj

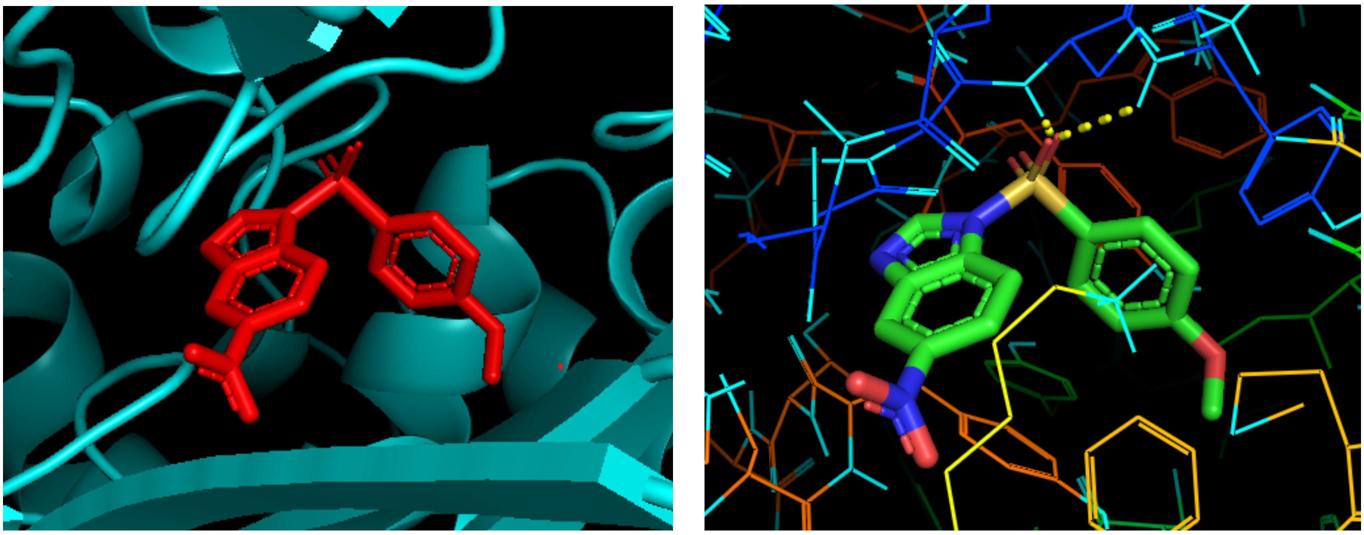

2ak

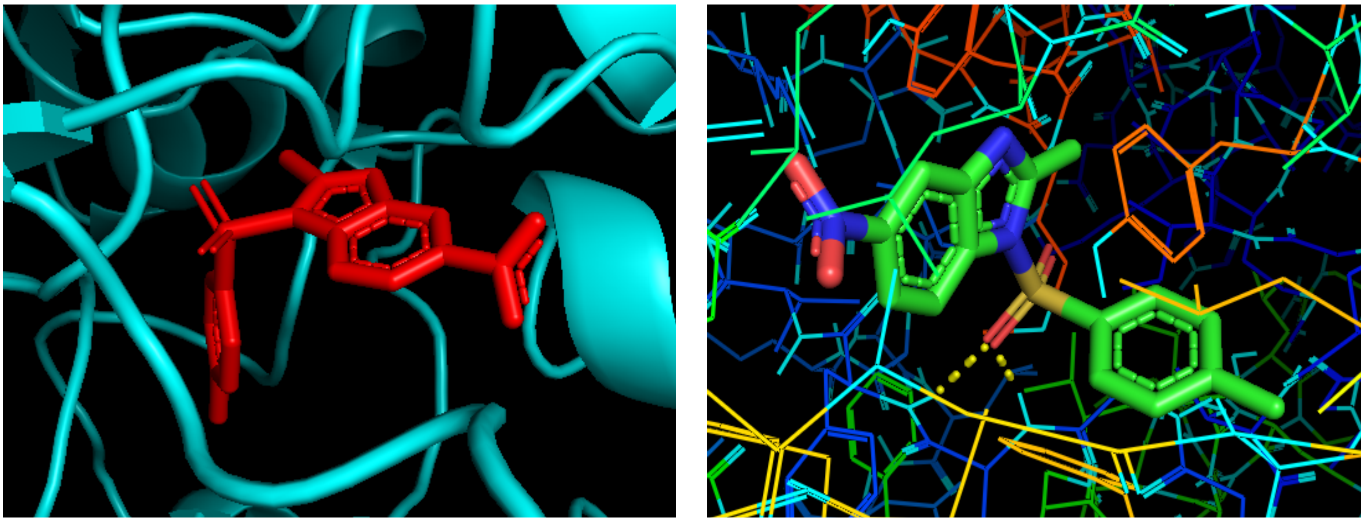

2bi

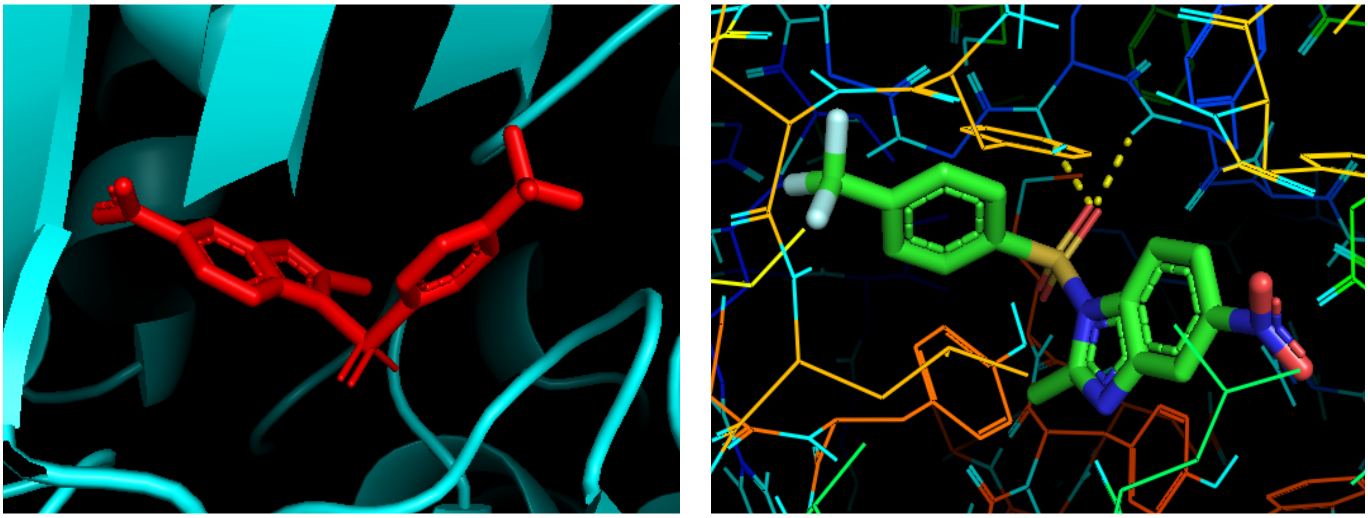

2bj

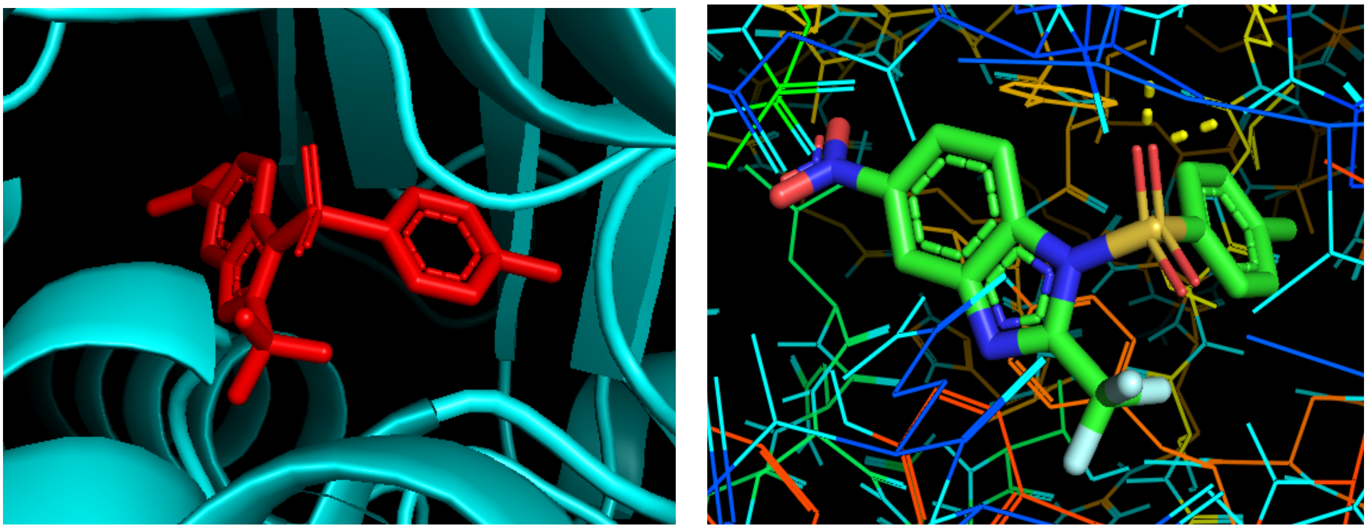

2ci

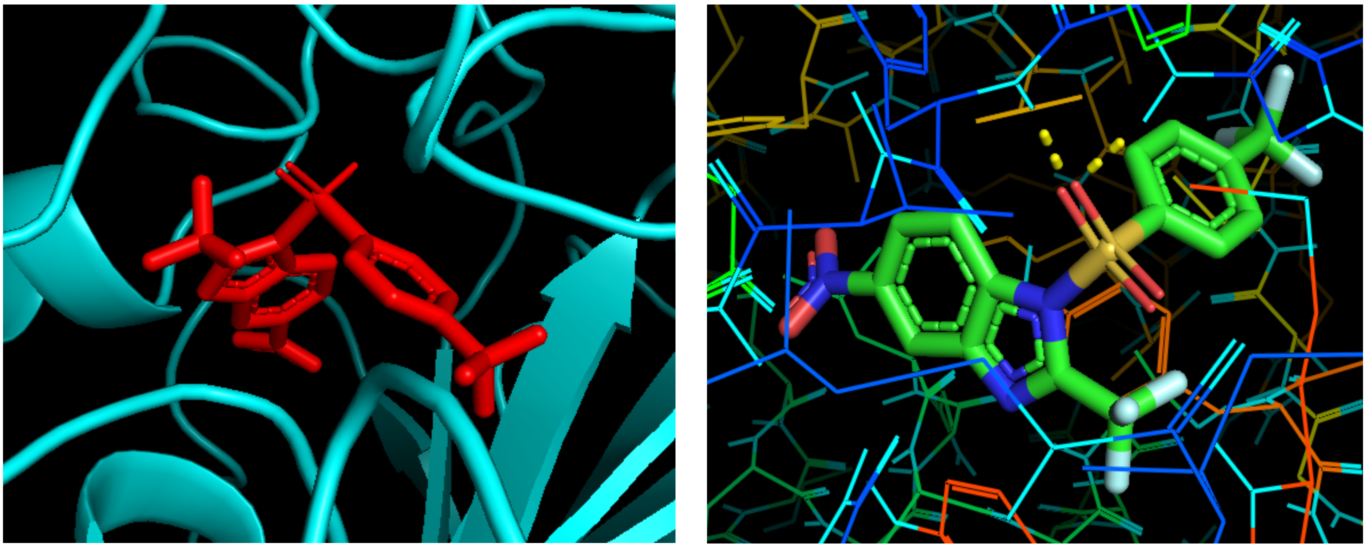

2cj

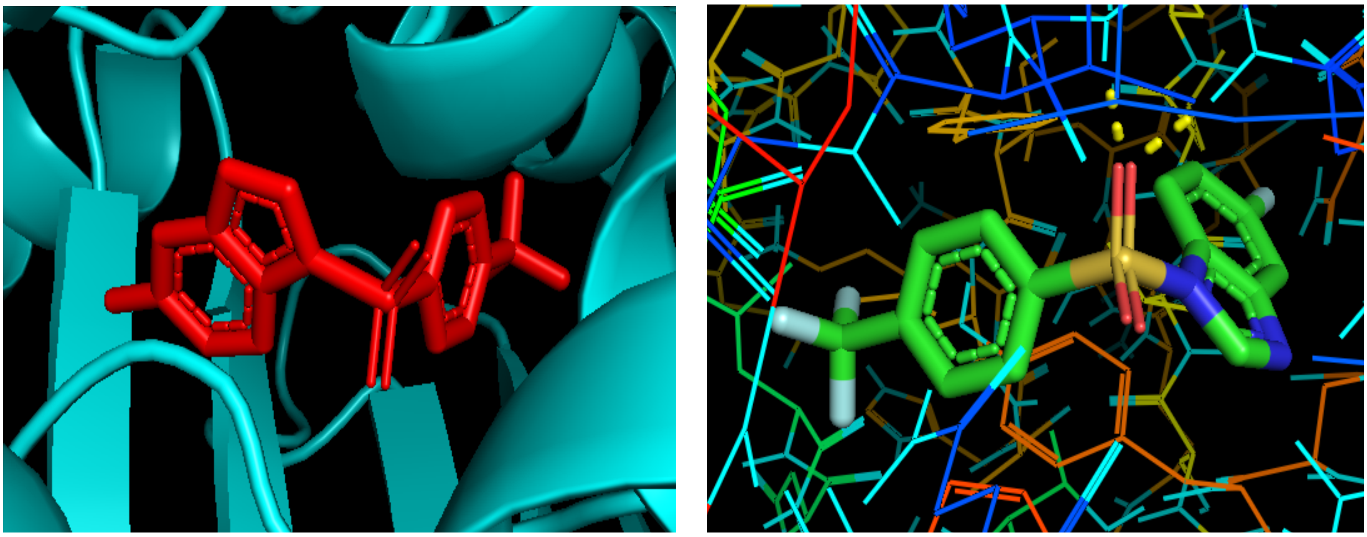

3aj

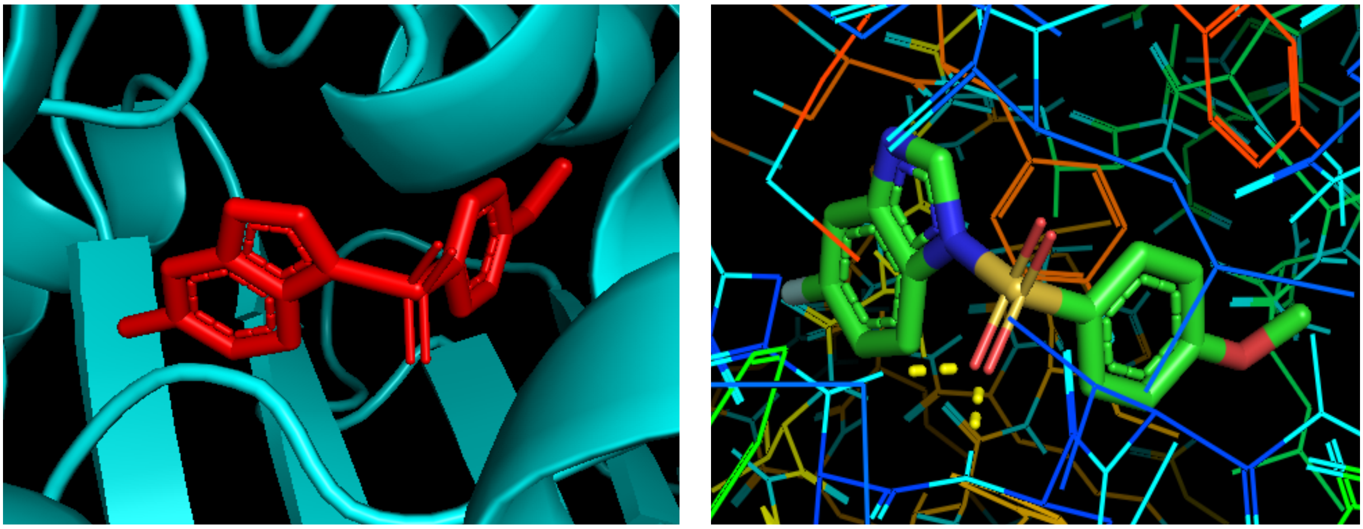

3ak

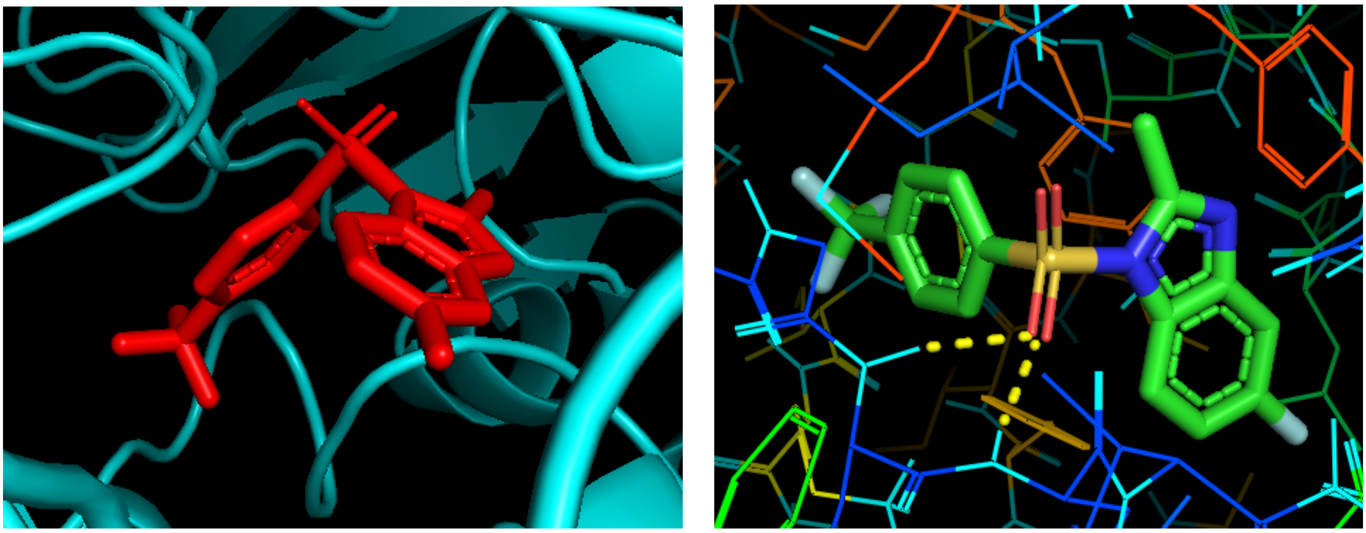

3bj

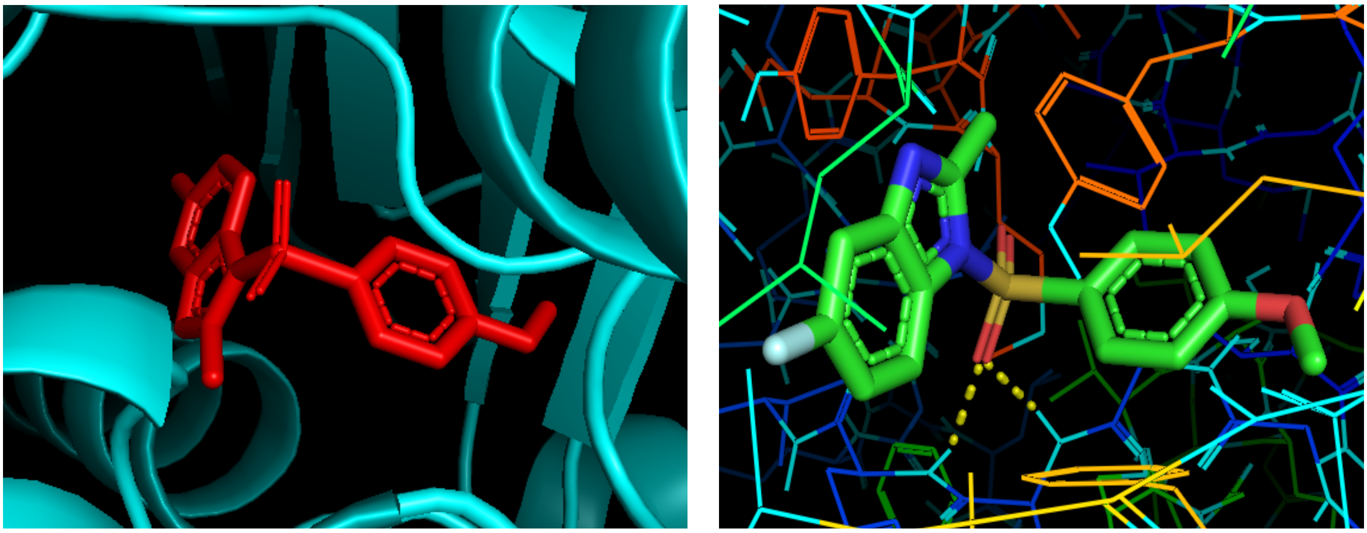

3bk

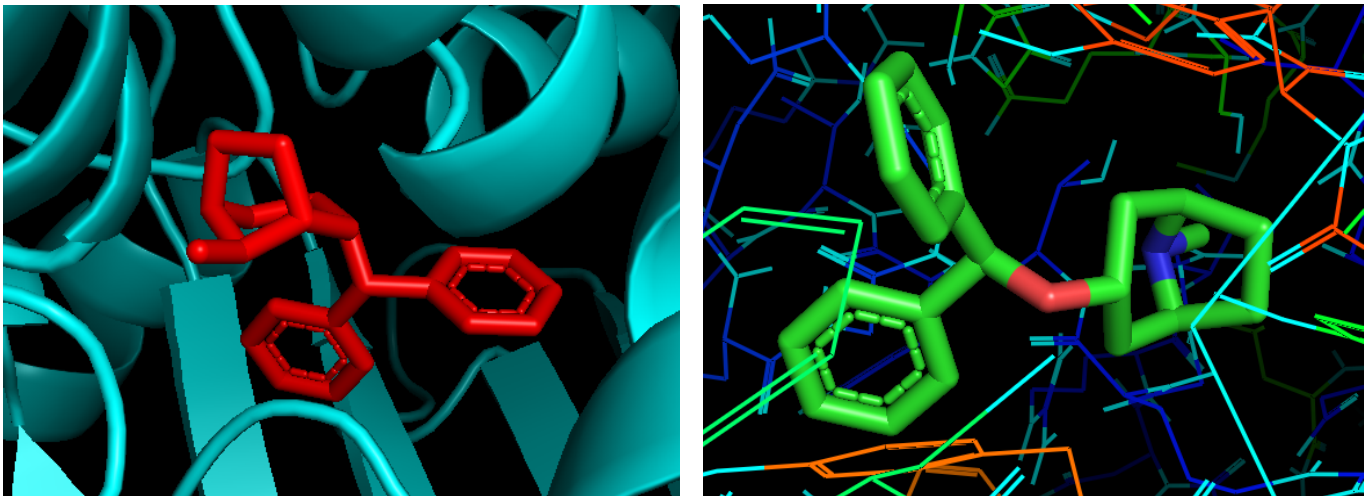

Benztropine (II)

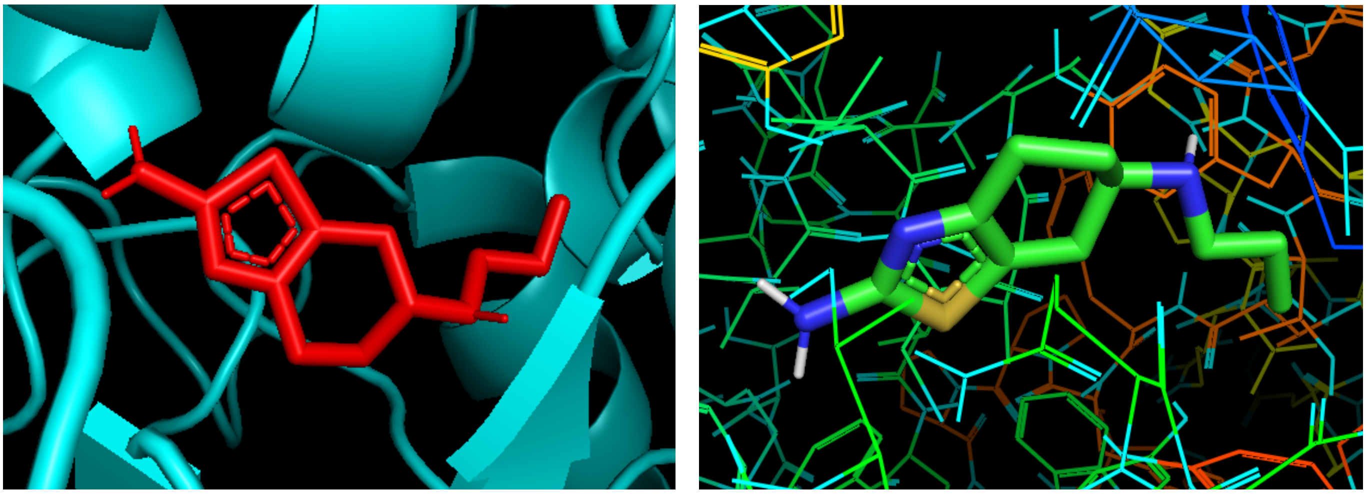

Pramipexole (III)

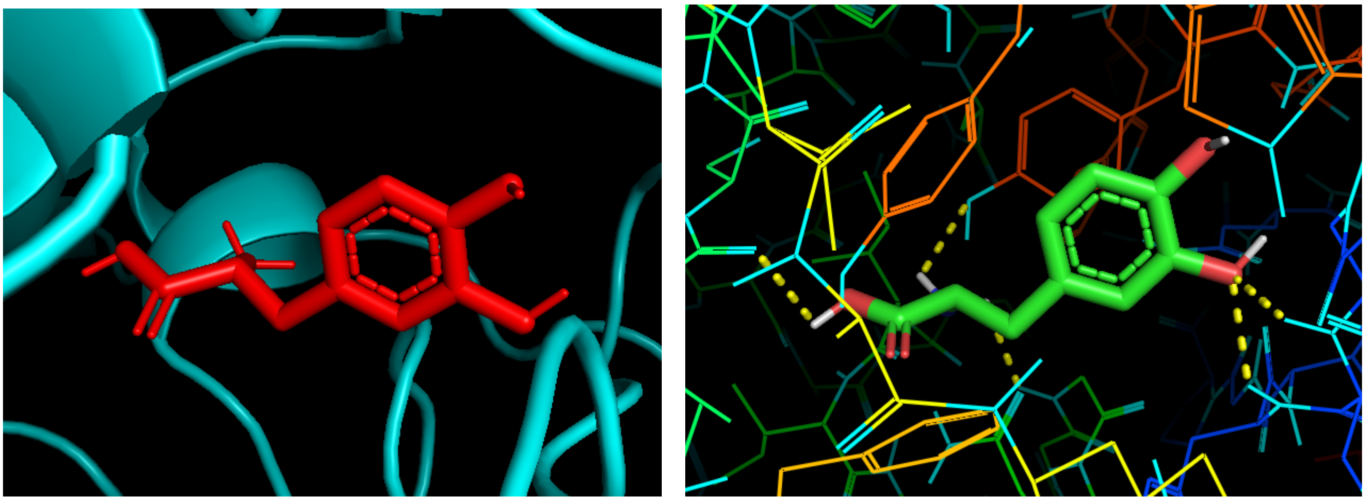

Levodopa (V)

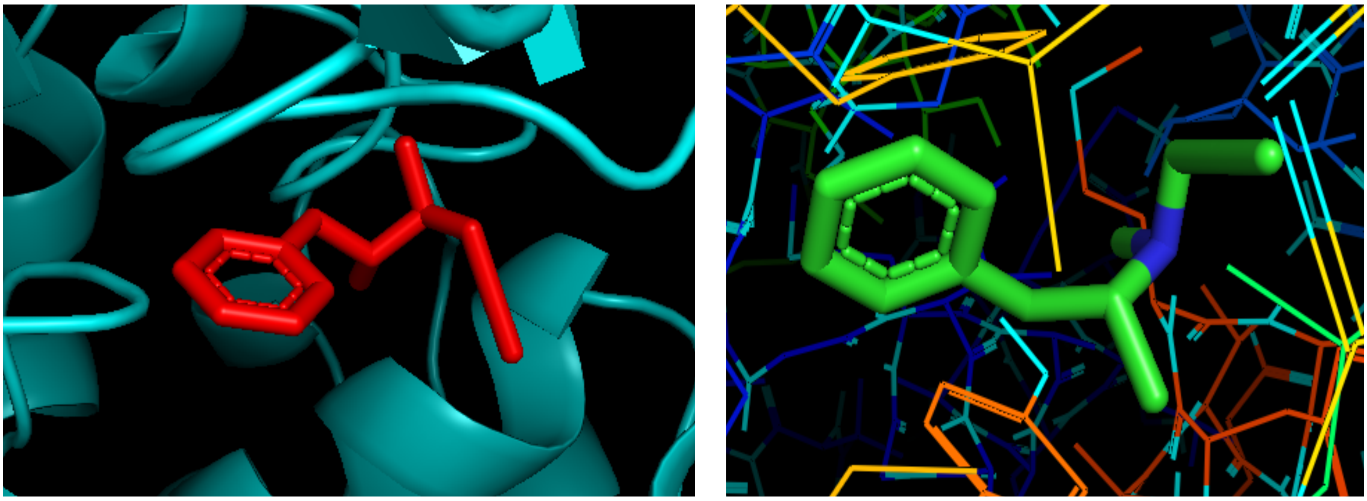

Selegiline (VI)

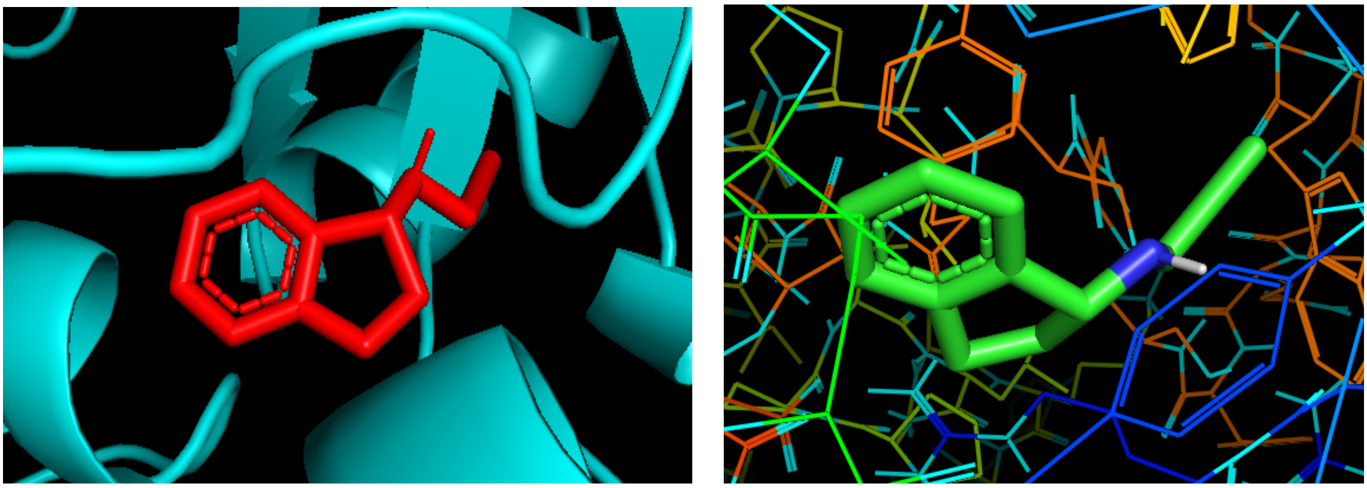

Rasagiline (VII)

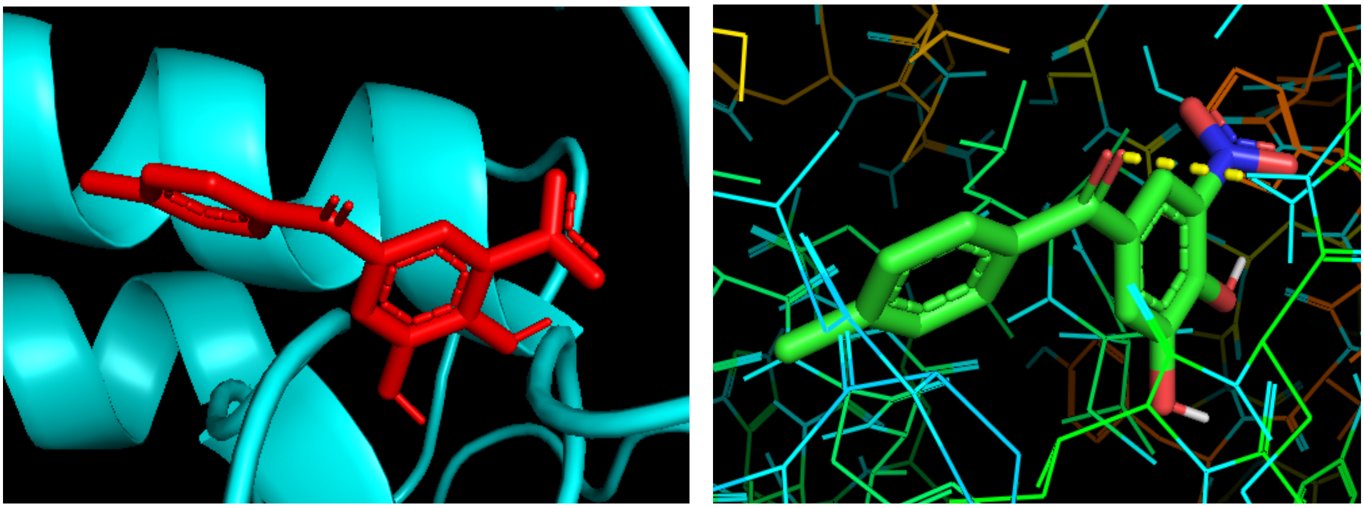

Tolcapone (VIII)

Figure S60: 3D Binding poses of selected hits 1ag-3bk and standard inhibitors with enlarged binding site (predicted by ADV)

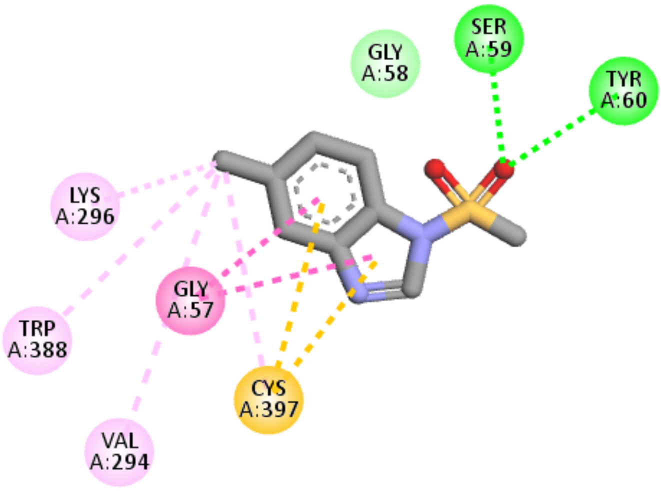

1ag

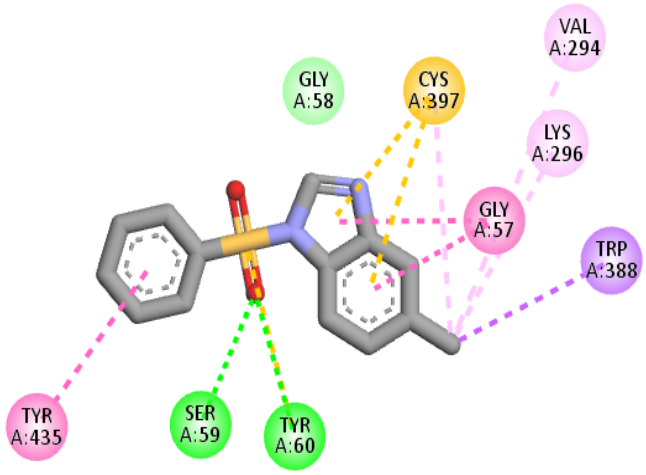

1ah

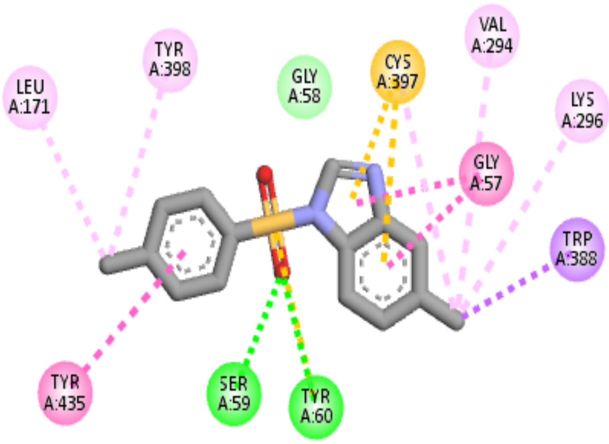

1ai

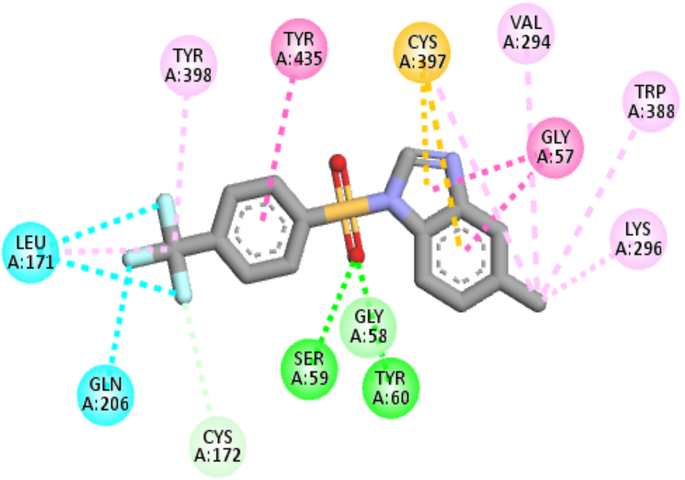

1aj

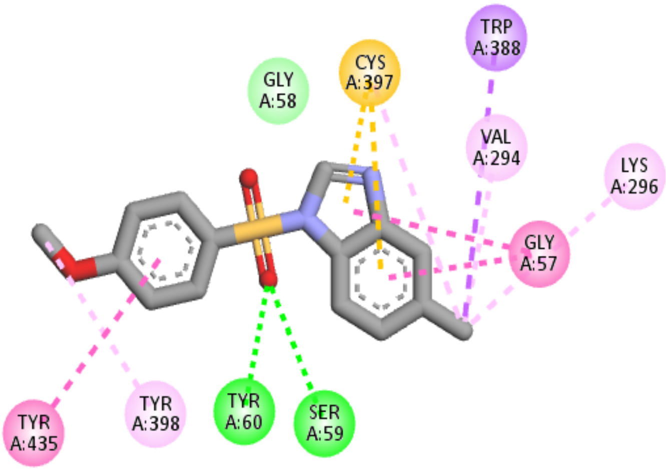

1ak

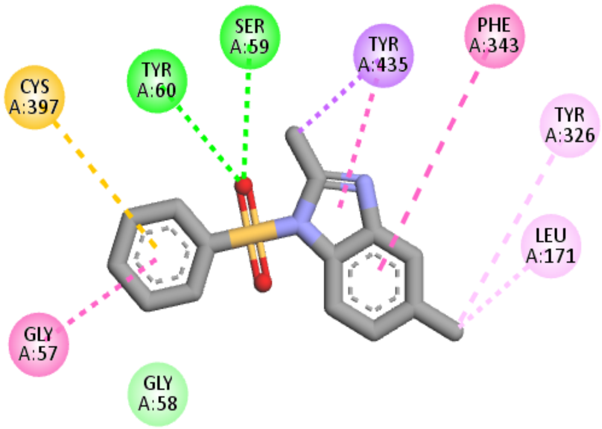

1bh

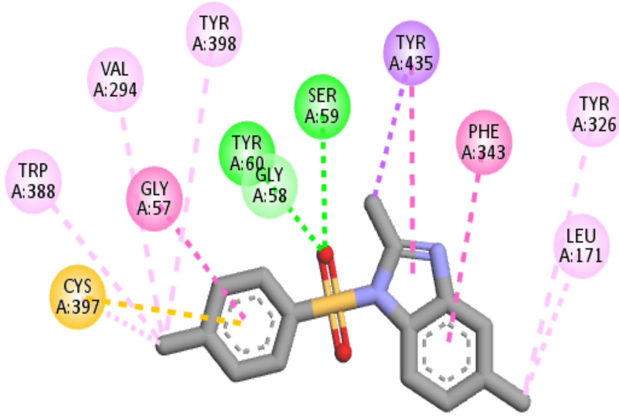

1bi

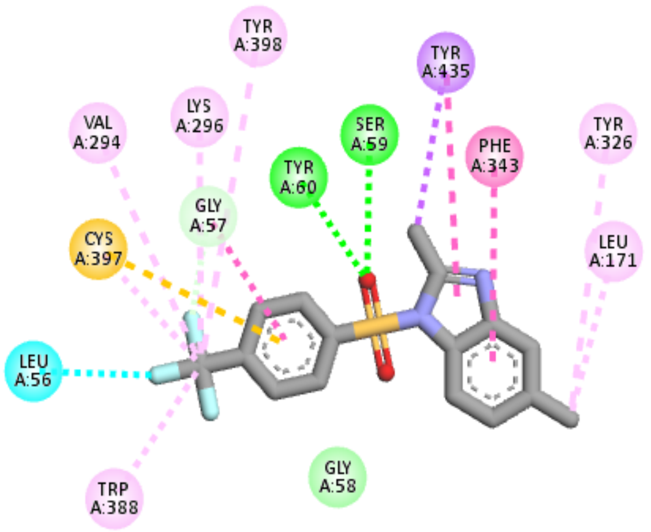

1bj

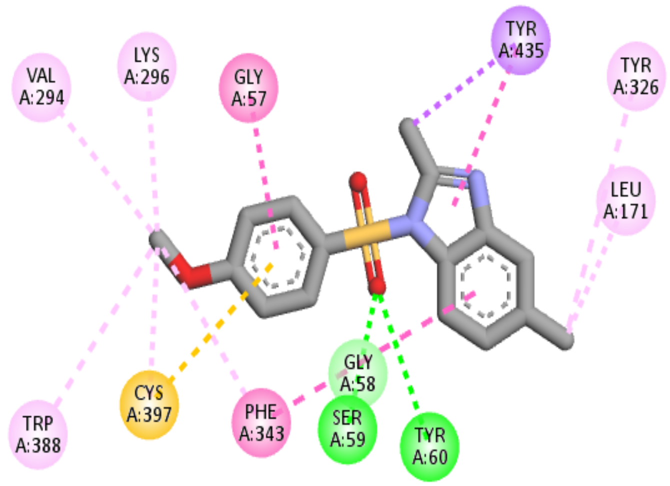

1bk

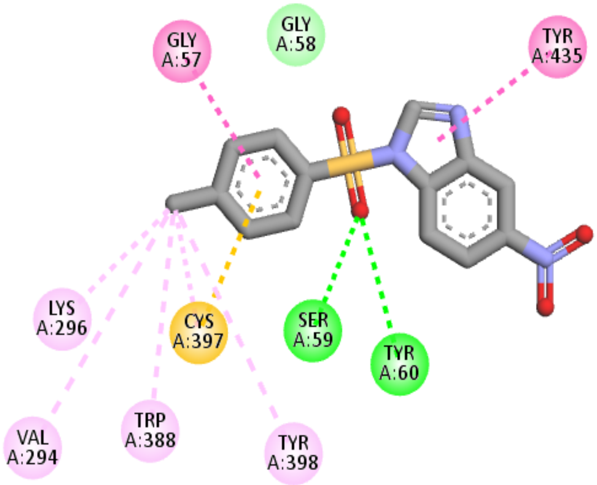

2ai

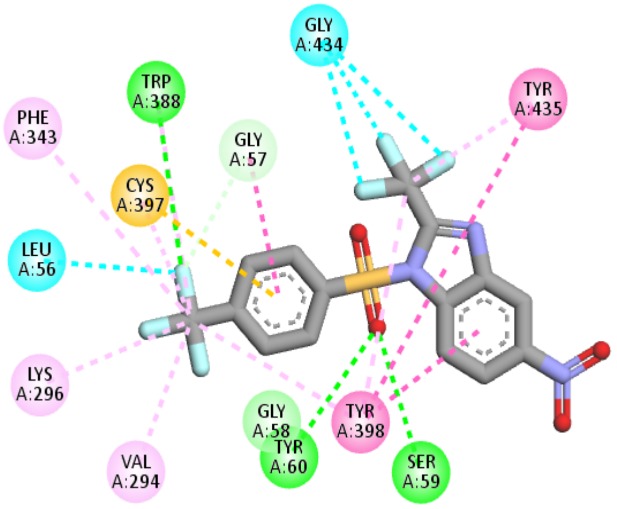

2aj

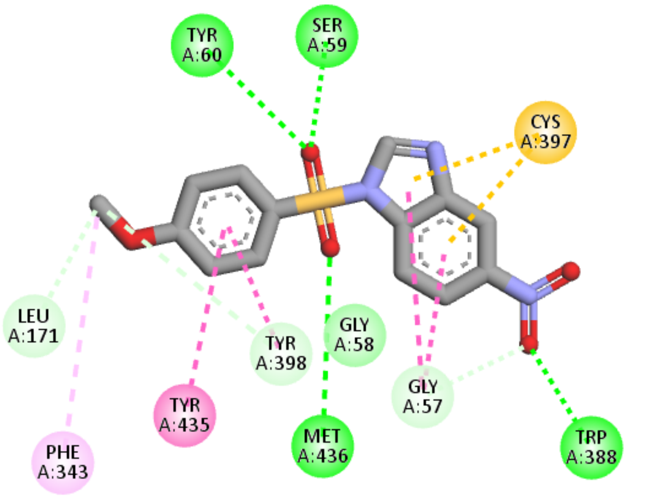

2ak

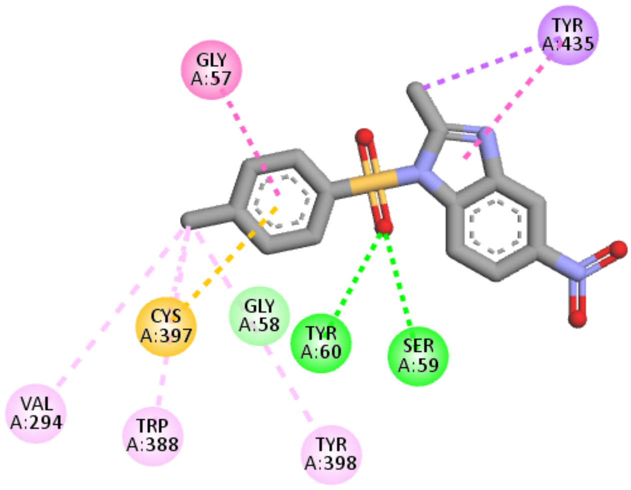

2bi

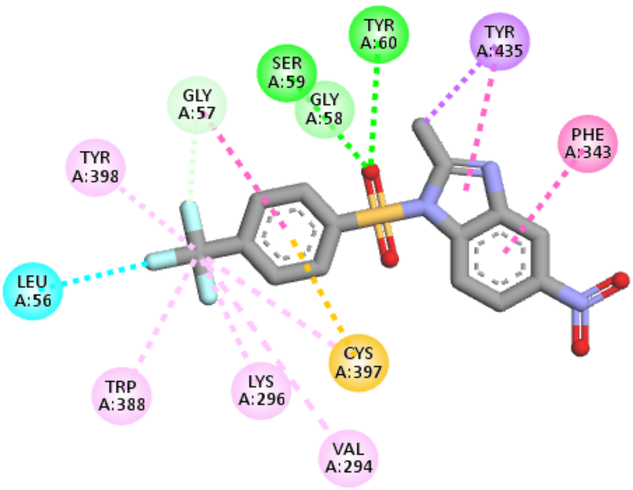

2bj

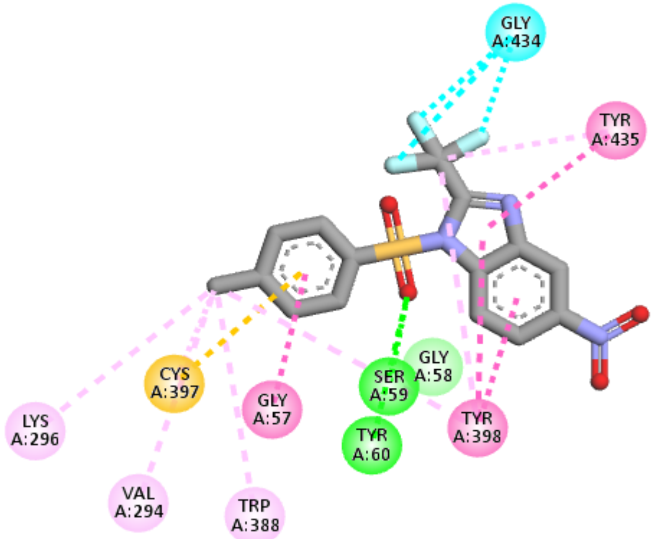

2ci

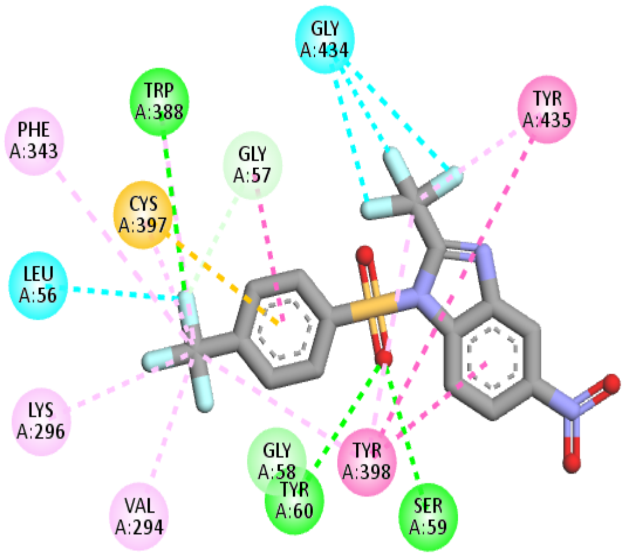

2cj

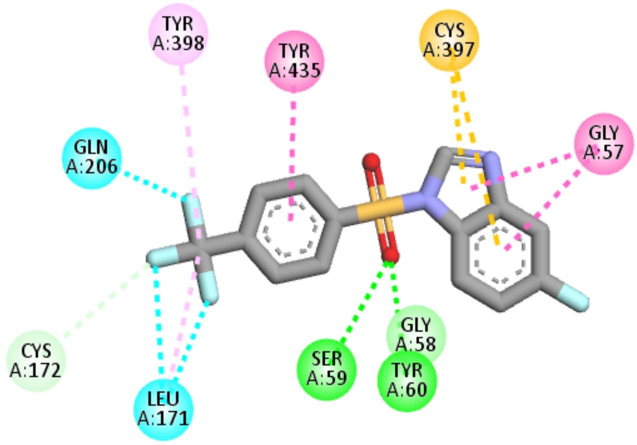

3aj

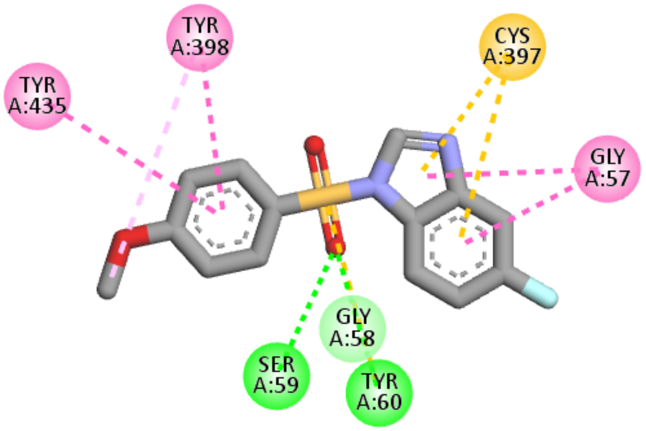

3ak

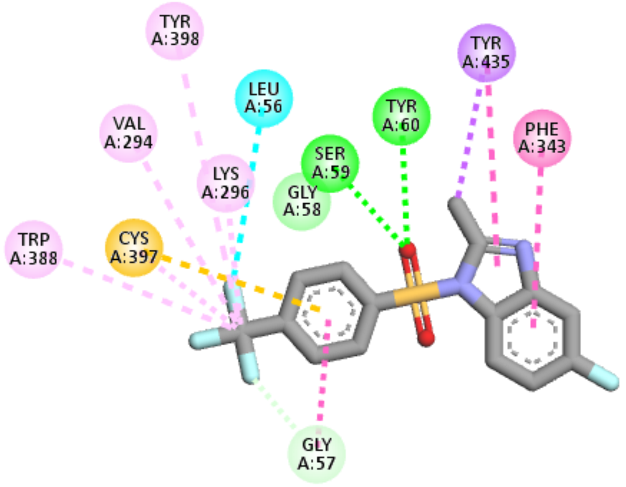

3bj

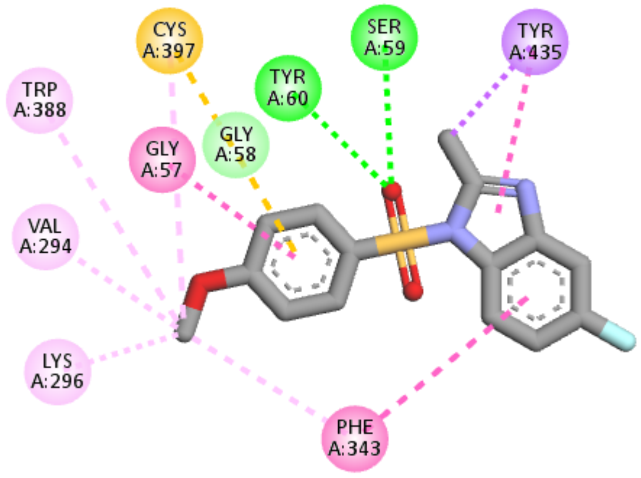

3bk

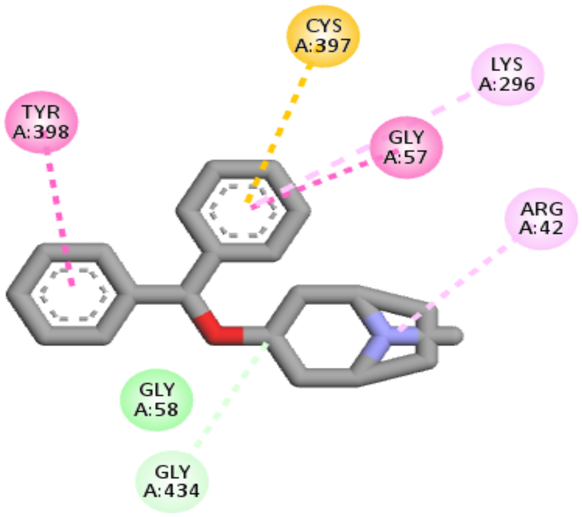

Benztropine (II)

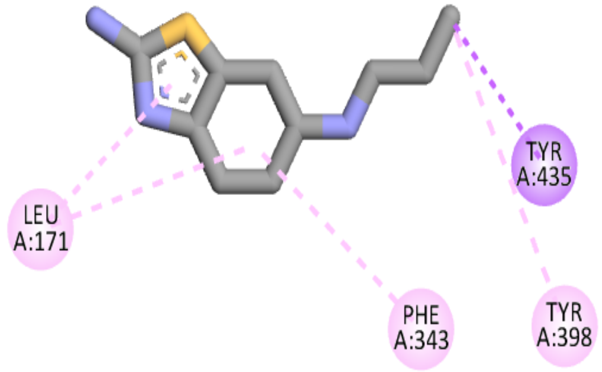

Pramipexole (III)

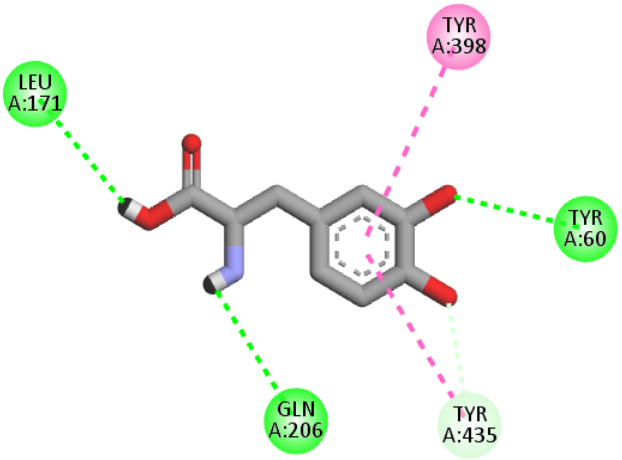

Levodopa (V)

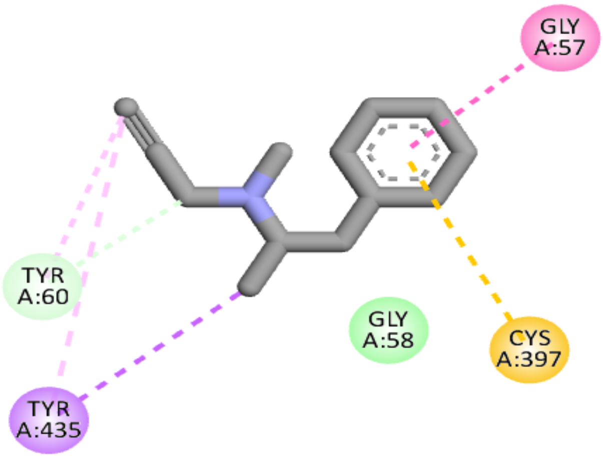

Selegiline (VI)

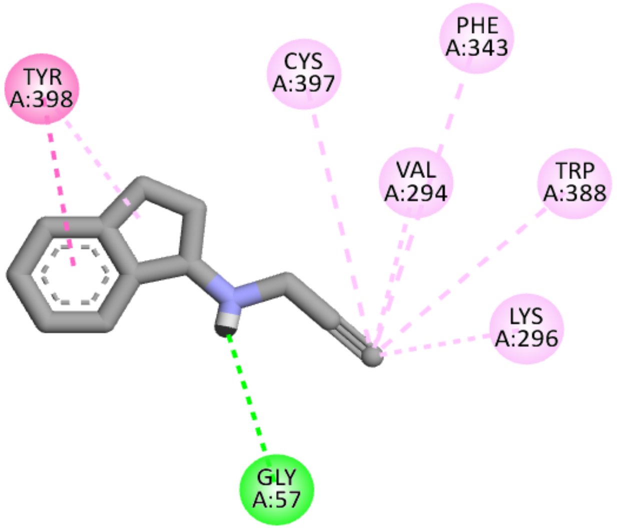

Rasagiline (VII)

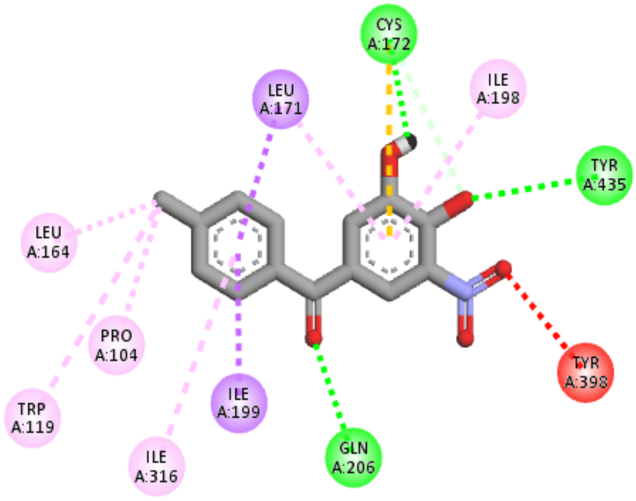

Tolecapone (VIII)

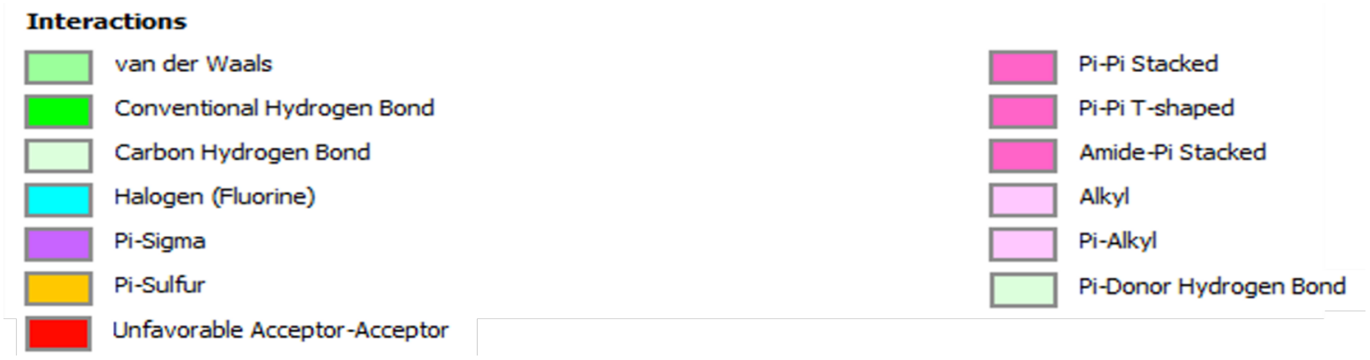

Figure S61: 2D interaction diagram of the derivatives (1ag-3bk) and standard inhibitors against 2C65 (Predicted by ADV)

Analysis of MD Simulation of MAO-B-2cj complex:

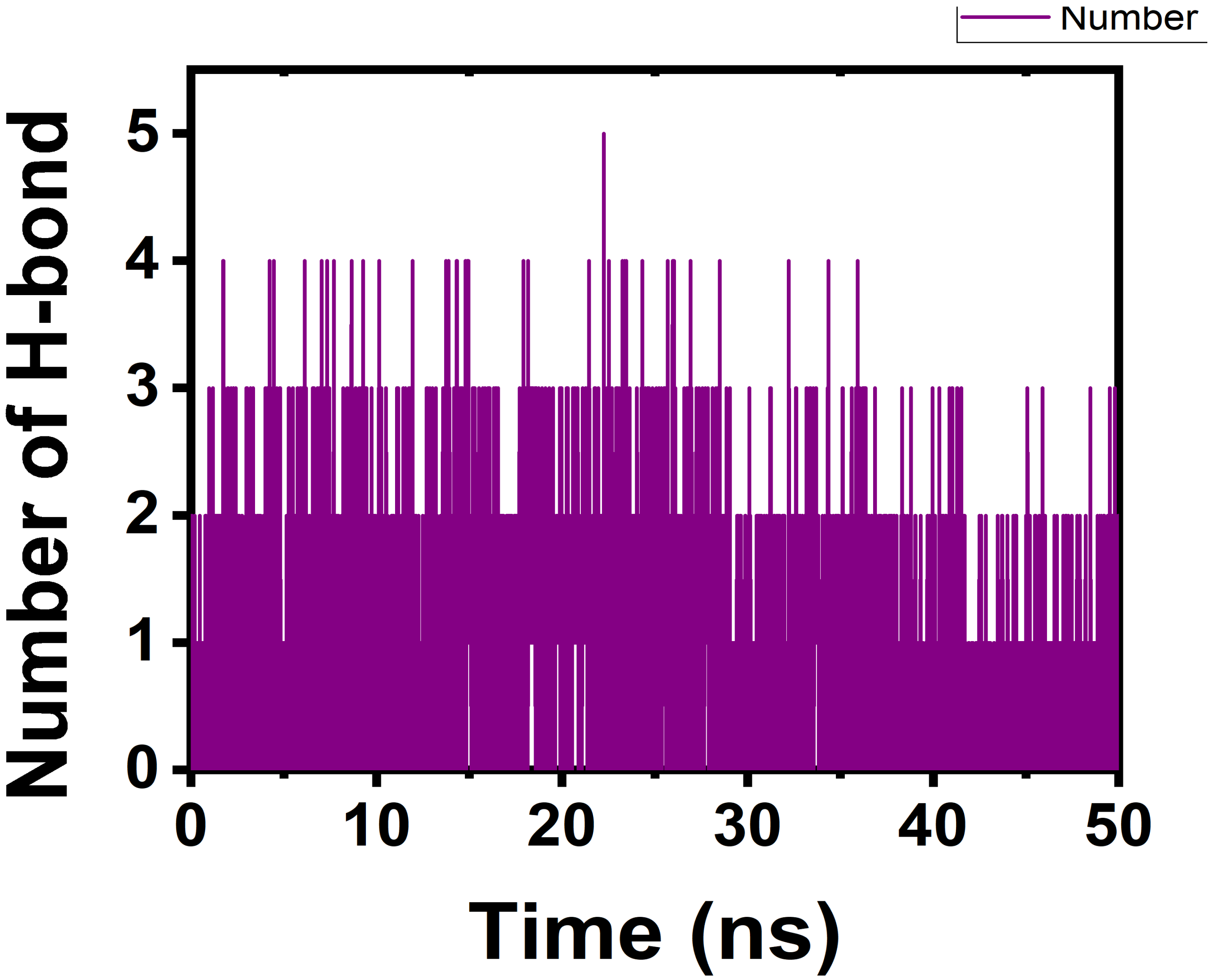

Figure S62: Changing the number of hydrogen bonds between MAO-B-2cj complex during 50 ns MD simulation.

Analysis of Physicochemical properties and Drug-likeness:

Table S2: The physicochemical properties of the benzimidazole derivatives and the two reference drugs

Compounds	Physicochemical Property										Drug-likeness | vLRoF = 0
	MW (g/mol)	NHBAs	NHBDs	TPSA ($Å^2$)	% Abs	MR	logS	$logD_{7.4}$	logP	Water Solubility (mg/mL)	
1ai	286.35	3	0	60.34	88.18	78.74	-3.863	2.407	2.89	0.0189	
1aj	340.32	6	0	60.34	88.18	78.77	-4.876	2.461	3.601	0.00673	
1bi	300.38	3	0	60.34	88.18	83.70	-4.112	2.622	3.199	0.00966	
1bj	354.35	6	0	60.34	88.18	83.74	-5.028	2.469	3.909	0.00336	
2ai	317.32	5	0	106.16	72.37	82.59	-3.692	1.785	2.49	0.0385	
2aj	371.23	8	0	106.16	72.37	82.63	-4.342	2.334	3.2	0.0131	
2bi	331.35	5	0	106.16	72.37	87.56	-4.014	2.068	2.798	0.0194	
2bj	385.32	8	0	106.16	72.37	87.59	-4.797	2.34	3.509	0.00649	
2ci	385.32	8	0	106.16	72.37	87.59	-4.803	2.461	3.509	0.00649	
2cj	439.29	11	0	106.16	72.37	87.63	-5.204	2.363	4.219	0.00208	
3aj	344.28	7	0	60.34	88.18	73.76	-4.696	2.395	3.431	0.00951	
3bj	358.31	7	0	60.34	88.18	78.73	-5.108	2.43	3.74	0.00475	
VI	187.28	1	0	3.24	107.88	61.31	-2.333	2.047	2.183	0.246	
VII	171.24	1	1	12.03	104.85	54.45	-2.412	1.733	1.897	0.932	

MW: Molecular Weight; **NHBAs**: Number of H-bond acceptors; **NHBDs**: Number of H-bond donors; **MR**: Molar Refractivity; **TPSA**: Topological Polar Surface Area; **logS**: Logarithm of the aqueous solubility (log mol/L); **logP**: Logarithm of the octanol/water partition coefficient; **$logD_{7.4}$**: Logarithm of the octanol/water partition coefficient at physiological pH 7.4; **vLRoF**: Violation of Lipinski's Rule of Five.

Pharmacokinetic properties Analysis:

Table S3: Absorption, distribution and excretion parameters of the benzimidazoles and the two reference drugs predicted using ADMETlab

Compounds	Absorption						Distribution			Excretion	
	Caco-2	P-gp inh	P-gp sub	HIA	F20%	F30%	PPB (%)	BBB	VD (L/kg)	$T_{1/2}$	CL
**1ai**	-4.32	0	0	1	1	1	83.991	1	-0.199	2.063	0.069
**1aj**	-4.453	0	0	1	1	1	88.326	1	-0.634	1.992	0.344
**1bi**	-4.349	0	0	1	1	1	87.492	1	-0.099	2.124	0.313
**1bj**	-4.455	0	0	1	1	1	89.232	1	-0.616	1.956	0.487
**2ai**	-4.427	0	0	1	1	1	81.928	1	-0.816	2.232	-0.112
**2aj**	-4.411	0	0	1	1	1	90.588	1	-0.974	2.094	0.05
**2bi**	-4.412	0	0	1	1	1	83.93	1	-0.786	2.068	-0.004
**2bj**	-4.455	0	0	1	1	1	90.969	1	-0.987	2.023	0.289
**2ci**	-4.453	0	0	1	1	1	91.78	1	-0.94	2.05	0.343
**2cj**	-4.472	0	0	1	1	1	90.032	1	-0.856	2.079	0.458
**3aj**	-4.421	0	0	1	1	1	88.536	1	-0.74	1.986	0.116
**3bj**	-4.459	0	0	1	1	1	89.131	1	-0.748	1.941	0.391
**VI**	-4.306	0	1	1	0	0	69.483	1	1.08	1.668	1.963
**VII**	-4.228	0	0	1	1	1	70.222	1	1.125	1.527	2.041

Caco-2: Caco-2 cell permeability; **P-gp inh**: P-glycoprotein Inhibitor; **P-gb sub**: P-glycoprotein Substrate; **HIA**: Human Intestinal Absorption; **F20%**: 20% Oral Bioavailability; **F30%**: 30% Oral Bioavailability; **PPB**: Plasma Protein Binding; **BBB**: Blood-Brain Barrier penetration probability; **VD**: Volume of Distribution; $\mathbf{T_{1/2}}$: Half-life (h); **CL**: Clearance of drug (mL/min/kg).

Table S4: Distribution and metabolism parameters of the benzimidazoles and the two reference drugs predicted using different webtools

Compounds	Distribution	Metabolism									
	Predicted by PreADMET	Predicted by SwissADME								Predicted by vNN	
	BBB prn	GI abn	P-gp sub	CYP 1A2 inhibitor	CYP 2C19 inhibitor	CYP 2C9 inhibitor	CYP 2D6 inhibitor	CYP 3A4 inhibitor	log Kp (cm/s)	HLM	MRTD (mg/day)
**1ai**	3.01085	High	No	Yes	Yes	Yes	No	No	-5.63	Yes	219
**1aj**	3.22997	High	No	Yes	Yes	Yes	No	No	-5.59	Yes	210
**1bi**	3.28107	High	No	Yes	Yes	Yes	No	No	-5.43	Yes	233
**1bj**	3.29595	High	No	Yes	Yes	Yes	No	No	-5.39	Yes	220
**2ai**	0.462548	High	No	No	Yes	Yes	No	No	-6.21	Yes	183
**2aj**	0.796664	High	No	Yes	Yes	Yes	No	No	-6.17	Yes	178
**2bi**	0.567217	High	No	No	Yes	Yes	No	No	-6.01	Yes	192
**2bj**	0.984906	Low	No	Yes	Yes	Yes	No	No	-5.97	Yes	191
**2ci**	1.35791	Low	No	Yes	Yes	Yes	No	No	-5.97	Yes	205
**2cj**	2.43872	Low	No	Yes	No	Yes	No	No	-5.93	Yes	205
**3aj**	3.30714	High	No	Yes	Yes	Yes	No	No	-5.81	Yes	183
**3bj**	3.55417	High	No	Yes	Yes	Yes	No	No	-5.61	Yes	198
**VI**	4.43519	High	No	Yes	No	No	Yes	No	-5.38	No	18
**VII**	6.341	High	No	No	No	No	Yes	No	-6.05	Yes	55

GI abn: Gastrointestinal tract Absorption; **BBB prn**: Blood-Brain-Barrier permeation; **P-gp sub**: P-glycoprotein Substrate; **log Kp**: Skin permeation; **CYP**: Cytochrome-P enzyme; **HLM**: Human Liver Microsomal stability; **MRTD**: Maximum Recommended Therapeutic Dose.

Toxicity profile/Toxicological endpoints Analysis:

Table S5: Pro Tox II predicted organ toxicity, toxicological endpoints and acute toxicity

Compound	Hepatotoxicity		Carcinogenicity		Immunotoxicity		Mutagenicity		Cytotoxicity		LD_{50} (mg/kg)	Toxicity Class
	Prediction	Probability	Prediction	Probability	Prediction	Probability	Prediction	Probability	Prediction	Probability		
**1ai**	Inact	0.66	Inact	0.54	Inact	0.99	Inact	0.69	Inact	0.74	69	3
**1aj**	Inact	0.59	Inact	0.61	Inact	0.99	Inact	0.71	Inact	0.78	21	2
**1bi**	Inact	0.65	Inact	0.55	Inact	0.99	Inact	0.66	Inact	0.75	69	3
**1bj**	Inact	0.59	Inact	0.57	Inact	0.98	Inact	0.70	Inact	0.83	21	2
**2ai**	Inact	0.51	Act	0.67	Inact	0.99	Act	0.59	Inact	0.81	1600	4
**2aj**	Act	0.53	Act	0.62	Inact	0.95	Act	0.56	Inact	0.85	21	2
**2bi**	Inact	0.50	Act	0.63	Inact	0.96	Act	0.61	Inact	0.81	21	2
**2bj**	Act	0.53	Act	0.57	Inact	0.81	Act	0.60	Inact	0.88	21	2
**2ci**	Act	0.53	Act	0.57	Inact	0.90	Act	0.60	Inact	0.88	21	2
**2cj**	Act	0.54	Act	0.55	Inact	0.83	Act	0.58	Inact	0.87	21	2
**3aj**	Inact	0.57	Inact	0.64	Inact	0.96	Inact	0.74	Inact	0.78	21	2
**3bj**	Inact	0.58	Inact	0.64	Inact	0.85	Inact	0.74	Inact	0.85	17	2
**VI**	Inact	0.98	Inact	0.66	Inact	0.97	Inact	0.88	Inact	0.79	385	4
**VII**	Inact	0.89	Inact	0.73	Inact	0.99	Inact	0.69	Inact	0.67	250	3

Act: Active; **Inact**: Inactive; **LD_{50}**: Median Lethal Dose.

Table S6: Toxicity profile of the benzimidazole derivatives and the two reference drugs predicted using different methods

Compounds	Toxicity						
	Predicted by ADMETlab				Predicted by vNN		
	hERG	H-HT	AMES	SkinSen	DILI	Cytotoxicity	MMP
**1ai**	0	1	0	0	Yes	No	No
**1aj**	0	1	0	0	Yes	No	No
**1bi**	1	1	0	0	Yes	No	No
**1bj**	1	1	0	0	Yes	No	No
**2ai**	0	1	0	0	Yes	No	No
**2aj**	0	1	0	0	Yes	No	No
**2bi**	0	1	0	0	Yes	No	No
**2bj**	0	1	0	0	Yes	No	No
**2ci**	0	1	0	0	Yes	No	No
**2cj**	0	1	0	0	Yes	No	No
**3aj**	0	0	0	0	Yes	No	No
**3bj**	0	1	0	0	Yes	No	No
**VI**	0	1	0	1	No	No	No
**VII**	0	1	0	1	No	No	No

hERG: hERG Potassium Channel Inhibition (Cardiotoxicity); **H-HT**: Human Hepatotoxicity; **AMES**: AMES Mutagenicity; **SkinSen**: Skin Sensitization; **DILI**: Drug-induced Liver Injury; **MMP**: Mitochondrial Membrane Potential toxicity.
